# ADRML: anticancer drug response prediction using manifold learning

**DOI:** 10.1038/s41598-020-71257-7

**Published:** 2020-08-28

**Authors:** Fatemeh Ahmadi Moughari, Changiz Eslahchi

**Affiliations:** 1 Department of Computer and Data Sciences, Faculty of Mathematical Sciences, Shahid Beheshti University, Tehran, Iran; 2 School of Biological Sciences, Institute for Research in Fundamental Sciences (IPM), Tehran, Iran

**Keywords:** Computational biology and bioinformatics, Cancer therapy, Drug discovery

## Abstract

One of the prominent challenges in precision medicine is to select the most appropriate treatment strategy for each patient based on the personalized information. The availability of massive data about drugs and cell lines facilitates the possibility of proposing efficient computational models for predicting anticancer drug response. In this study, we propose ADRML, a model for Anticancer Drug Response Prediction using Manifold Learning to systematically integrate the cell line information with the drug information to make accurate predictions about drug therapeutic. The proposed model maps the drug response matrix into the lower-rank spaces that lead to obtaining new perspectives about cell lines and drugs. The drug response for a new cell line-drug pair is computed using the low-rank features. The evaluation of ADRML performance on various types of cell lines and drug information, in addition to the comparisons with previously proposed methods, shows that ADRML provides accurate and robust predictions. Further investigations about the association between drug response and pathway activity scores reveal that the predicted drug responses can shed light on the underlying drug mechanism. Also, the case studies suggest that the predictions of ADRML about novel cell line-drug pairs are validated by reliable pieces of evidence from the literature. Consequently, the evaluations verify that ADRML can be used in accurately predicting and imputing the anticancer drug response.

## Introduction

Precision medicine aims to finely select treatments for cancer based on the genetic information of individual patients^1^. One of the highly critical problems in precision medicine is predicting anticancer drug response for each patient^2–4^. Due to the heterogeneity of tumors, the patients with the same type of cancer may show various therapeutic responses toward similar drugs^5^. Therefore, providing computational methods to discover the relationship between genomic information and drug sensitivity is of high importance and can be beneficial in precision medicine^3,6^.

Genomics of Drug Sensitivity in Cancer (GDSC)^7^ and Cancer Cell Line Encyclopedia (CCLE)^8^ are two projects that have provided molecular profiles and drug response values for hundreds of cancer cell lines against several anticancer drugs. These large datasets facilitate the development of computational methods for anticancer drug sensitivity prediction. Numerous computational methods have been proposed to predict drug response using gene expression profile, or other molecular information of cell lines. Some of the computational methods have considered drug information such as chemical substructure of drugs, besides made use of cell line information. In the proposed computational methods, various machine learning methods have been utilized such as sparse linear regression^4,9–11^, random forest^2,12,13^, kernel-based methods^4,14–17^, matrix factorization^1,18–20^, neural networks and deep learning^21–24^.

Wang et al. have proposed a Similarity Regularized Matrix Factorization (SRMF) method, which utilizes the similarity of cell lines based on gene expression profiles and chemical substructure similarity of drugs to predict anticancer drug sensitivity^1^. They also conducted drug-repurposing and suggested new potential treatments for cell lines with Non-small Cell Lung Cancer (NSCL). It is suggested that patients who have similar genomic properties reveal similar responses to similar drugs^1^. Based on the SRMF study, Suphavilai et al. have proposed a recommender system called “CaDRReS” that can predict drug response for unseen cell lines^19^. Furthermore, they showed that latent space features are correlated with associated pathways of drugs. They did not consider any features of drugs for predicting the drug response values. Afterwards, Chang et al. have devised “CDRscan”, an ensemble model containing five Convolutional Neural Networks (CNNs)^21^. They made use of mutational profiles of cell lines and chemical substructure of drugs as the input features to these CNNs. The drug response values were measured by averaging the output of five CNNs. Moreover, they have repurposed multiple non-oncology drugs as the potential therapeutic agents for cancer cell lines. Recently, Wei et al. have suggested a simple cell line-drug complex network called “CDCN”^25^. They constructed a complex network from various information, including cell line similarities, drug similarities, and drug responses, and inferred unknown drug response from the network. They also proposed a generalized version that can predict the drug response for new drugs and new cell lines. Despite its simple structure, CDCN had satisfying results in imputing missing drug responses.

Nevertheless, the proposed methods had moderate performance and do not analyze several types of features for cell lines and drugs. Thus, investigating the influence of various features for cell lines and drugs in predicting therapeutic response is still in need and challenging. We investigate three types of cell line features, namely gene expression, mutation profile, and copy number variation, in addition to three types of drug features, including chemical substructure, target proteins, and associated KEGG pathways. In this work, we propose ADRML, Anticancer Drug Response Prediction, by using Manifold Learning. ADRML constructs a bipartite graph between drug and cell lines, and then decompose its adjacency matrix using similarity-constrained manifold learning into two lower-dimensional latent matrices. The proposed method is capable of predicting therapeutic response for new cell lines and new drugs. The similarity-constrained manifold learning previously has been used in the context of drug-disease association prediction^26^ and drug–drug interaction prediction^27^, which yielded accurate performance.

The performance of ADRML is measured using various types of cell line similarities and drug similarities and is compared to the recently proposed methods on both GDSC and CCLE datasets. Moreover, the rationality of ADRML predictions is confirmed by analyzing the association between the predicted drug response values and activity scores of Biocarta pathways. Finally, conducting case studies on the predictions of ADRML for unknown drug response in literature and reliable databases verifies its capability in predicting unknown drug response and admits that ADRML obtains accurate results for new pairs of cell line-drug.

## Results

### Benchmark datasets and collected features

In this work, we used two pharmacogenomic datasets, namely the Genomics of Drug Sensitivity in Cancer (GDSC)^7^ and Cancer Cell Line Encyclopedia (CCLE)^8^. Among several types of data in these datasets, various information including the half-maximal inhibitory concentration (*IC*50), the gene expression profile, copy number variation, and mutation profile was downloaded by using *PharmacoGx* R package^28^. The collected genes were accessible in the COSMIC database^29^, and the collected drugs were restricted to the drugs with a Compound ID (CID) in the PubChem database^30^.

Some values of IC50, copy number variation, and mutation profiles in both datasets were missing. A pre-processing procedure was applied, according to Lu et al.^2^ to impute the missing values, which is fully described in “Pre-processing to impute the missing data”. After applying the pre-processing steps, the GDSC dataset contained 98 drugs and 555 cell lines from 19 cancer types, as defined by The Cancer Genome Atlas (TCGA)^31^, and the CCLE dataset contained 24 drugs and 363 cell lines from 22 cancer types as defined by TCGA. Furthermore, several types of information about drugs were obtained from the following databases:

- The fingerprints of canonical simplified molecular-input line-entry (SMILES) were obtained from PubChem^30^.
- The target proteins were collected from GDSC, DrugBank^32^, and literature.
- The KEGG-pathways related to the drugs were downloaded from the STiTCH database^33^.

A brief description of the collected data is presented in Table 1.

**Table 1:** The number of collected samples and features.

Dataset	Cell line	Drug	Tissue types	Expression profile	Mutation profile	Copy number variation	Drug fingerprint	Target protein	KEGG pathway
CCLE	363	24	22	19,389	1,667	24,960	881	76	124
GDSC	555	98	19	11,712	54	24,959	881	–	–

The cell line features such as gene expression profile, mutation profile, copy number variation, tissue types, drug names, and drug response values were downloaded from PharmacoGx package, Drug fingerprints were obtained from pubchem, target proteins were gathered mainly from GDSC and DrugBank, and KEGG pathways were obtained from STiTCH database.

### Hyper-parameter tuning

**Figure 1:**
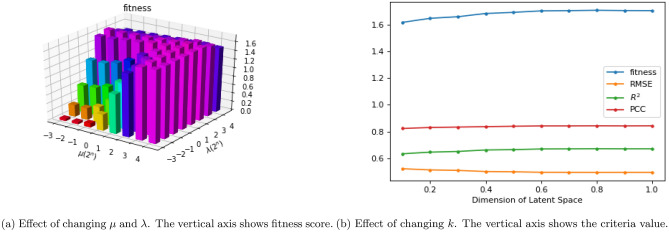
The effect of choosing different values of hyper-parameters on ADRML performance.

ADRML model is fully described in “Methods” which has three hyperparameters: “*k*” is the dimension of latent space, “$\mu$” is the regularization coefficient, and “$\lambda$” is the similarity conservation coefficient. In order to map the response matrix into lower dimensional space, “*k*” value was considered to be less than the number of cell lines and drugs. For simplicity, we considered $k = k'\%\ of min(number\ of\ cell\ lines, \ number\ of \ drugs)$. We tuned the hyper-parameter values using grid search. We executed ADRML with fivefold cross-validation on all pairs of cell line and drug for all combinations of $k\in \{10\%,20\%,...,90\%\}$, $\lambda \ and\ \mu \in \{2^{-3},2^{-2},2^{-1},2^{0},2^{1},2^{2}, 2^{3}\}$. The hyper-parameters were tuned on CCLE dataset, using gene expression similarity of cell lines and chemical similarity of drugs by maximizing a fitness score (briefly mentioned as *fitness* in the following).

1 $$\begin{aligned} fitness=R^{2}+PCC-RMSE \end{aligned}$$

where the evaluation criteria including Coefficient of Determination ($R^2$), Pearson Correlation Coefficient (*PCC*), and Root Mean Square Error (*RMSE*) are completely explained in “Evaluation criteria”. The definition of fitness score is logical since the best model is the one with the highest values of $R^2$ and *PCC*, and the lowest value of *RMSE*. ADRML achieved the best results when $k=70\%,\ \mu =2^{3}$ and $\lambda =2^2$. We considered the same hyper-parameter values for all types of similarities in CCLE and GDSC. In order to illustrate the impact of $\mu$ and $\lambda$ on the fitness score, we fixed the latent dimension to $k=70\%$ and depicted the fitness function in a 3D-histogram of Fig. 1a. It is evident that when $\lambda$ is small, the fiteness function is increasing with regard to $\mu$. Conversely, when $\mu$ is small, the larger $\lambda$ values leads to higher fitness score.

Moreover, the values of $\mu =2^3$ and $\lambda =2^2$ were fixed and the influence of latent space dimension was examined. Figure 1b demonstrates that the greater dimensions of latent space leads to higher fitness score. Moreover, *PCC*, and $R^2$ improves by increasing *k*, while *RMSE* declines as *k* grows larger. However, the criteria value do not change or have subtle changes after $k=70\%$.

### Performance of ADRML prediction

We investigated the effects of using different similarity constraints on ADRML performance. Several cell line similarities based on gene-expression, mutation, and copy number variation, and multiple drug similarities based on chemical substructure, target proteins, and KEGG pathways were considered as the constraints of manifold learning.

Table 2 summarizes the performance of ADRML for every combination of cell line and drug similarity. Each pair of cell line and drug similarity is shown in one row and the columns show the computed criteria. Clearly, ADRML yields both accurate and robust performance in each scenario, because the results of all conditions are quite high and close to each other. However, it achieves the best results using similarity of cell lines based on gene expression and similarity of drugs based on target proteins, which yields $RMSE=0.487, \ R^2=0.682, \ PCC=0.846$ . We used these two similarities for further evaluations.

**Table 2:** Performance of ADRML on various types of similarities.

Cell line similarity	Drug similarity	RMSE	$$ {R^2 }$$	PCC	Fitness
Gene expression	Chemical	0.4927	0.675	0.8454	1.0277
Mutation	Chemical	0.4935	0.6739	0.8446	1.025
Copy number variation	Chemical	0.4964	0.6701	0.8368	1.0105
Gene expression	Target protein	**0**.**487**	**0**.**682**	**0**.**846**	1.**041**
Mutation	Target protein	0.4894	0.6794	0.844	1.034
Copy number variation	Target protein	0.4992	0.6664	0.8319	0.9991
Gene expression	KEGG pathways	0.5003	0.6651	0.8453	0.999
Mutation	KEGG pathways	0.5004	0.665	0.8452	0.9993
Copy number variation	KEGG pathways	0.5045	0.6595	0.8385	0.9982

The performance of each model is evaluated using fivefold cross-validation on cell line-drug pairs and using $k=70\%$, $\mu =2^3$, and $lambda=2^2$. Each row shows the performance of ADRML on a pair of cell line and drug similarity. The best results of each criteria is shown in bold face.

In order to investigate ADRML performance on each drug, we depicted the drug-wise correlation plots. Figures 2 and 3 illustrated the pearson correlation between the observed and the predicted $\log IC50$ for four drugs in CCLE and GDSC datasets, respectively. The figures show high drug-wise *PCC* and validate that ADRML can predict drug responses with high correlation to the real responses. The majority of data in these scatter plots are centered near the fitted line. It is notable that the outliers are natural due to the technical noises in gene expression data, or inconsistency of drug responses in CCLE and GDSC^5,25,34,35^. Further plots for drug-wise *PCC* of GDSC are shown in Supplementary Figs. S1–S98 and the drug-wise *PCC* of CCLE are shown in Supplementary Figs. S99–S122.

**Figure 2:**
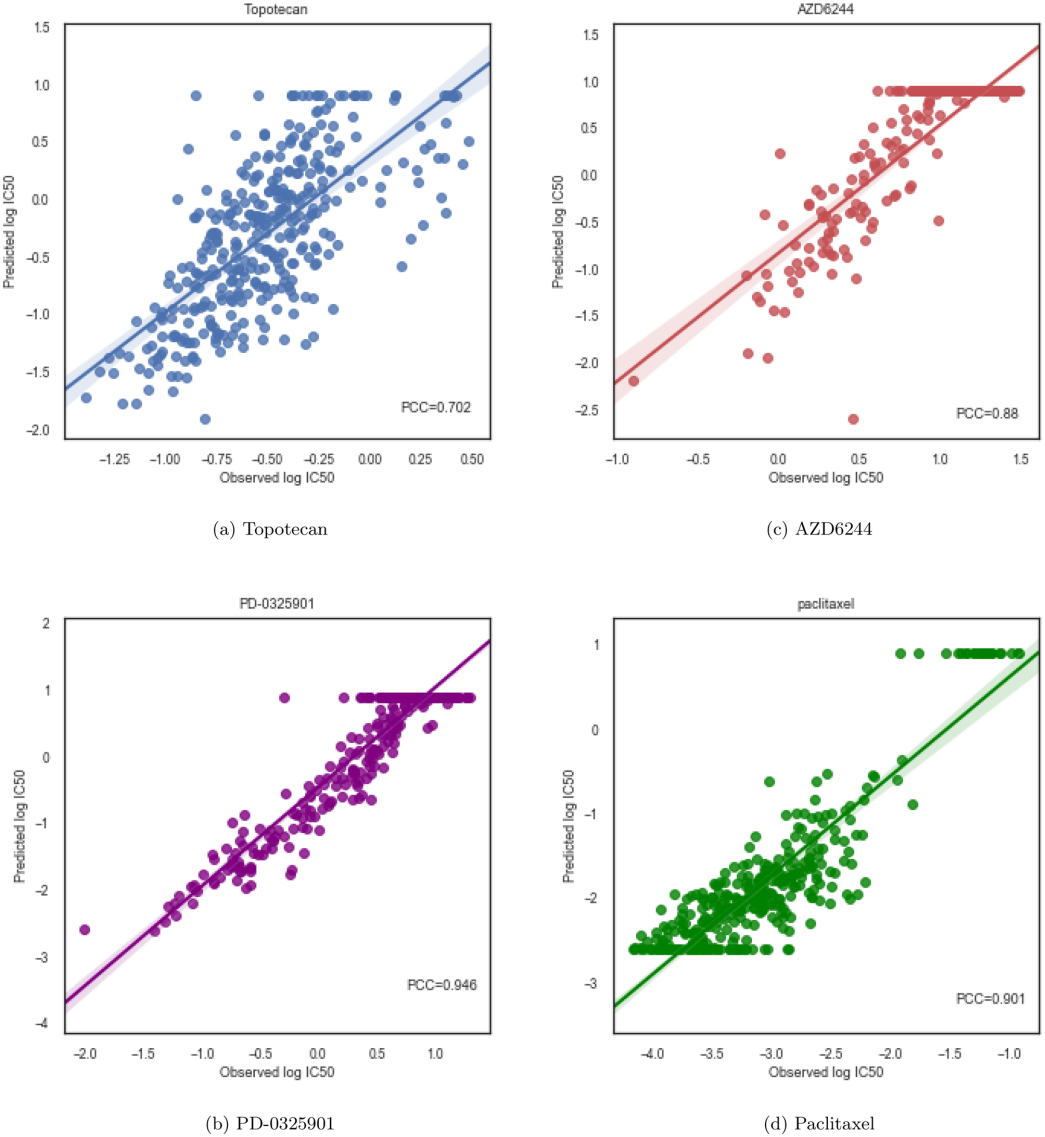
Drug-wise *PCC* for 4 drugs in CCLE. The computed *PCC* is illustrated in lower right corner of each plot.

**Figure 3:**
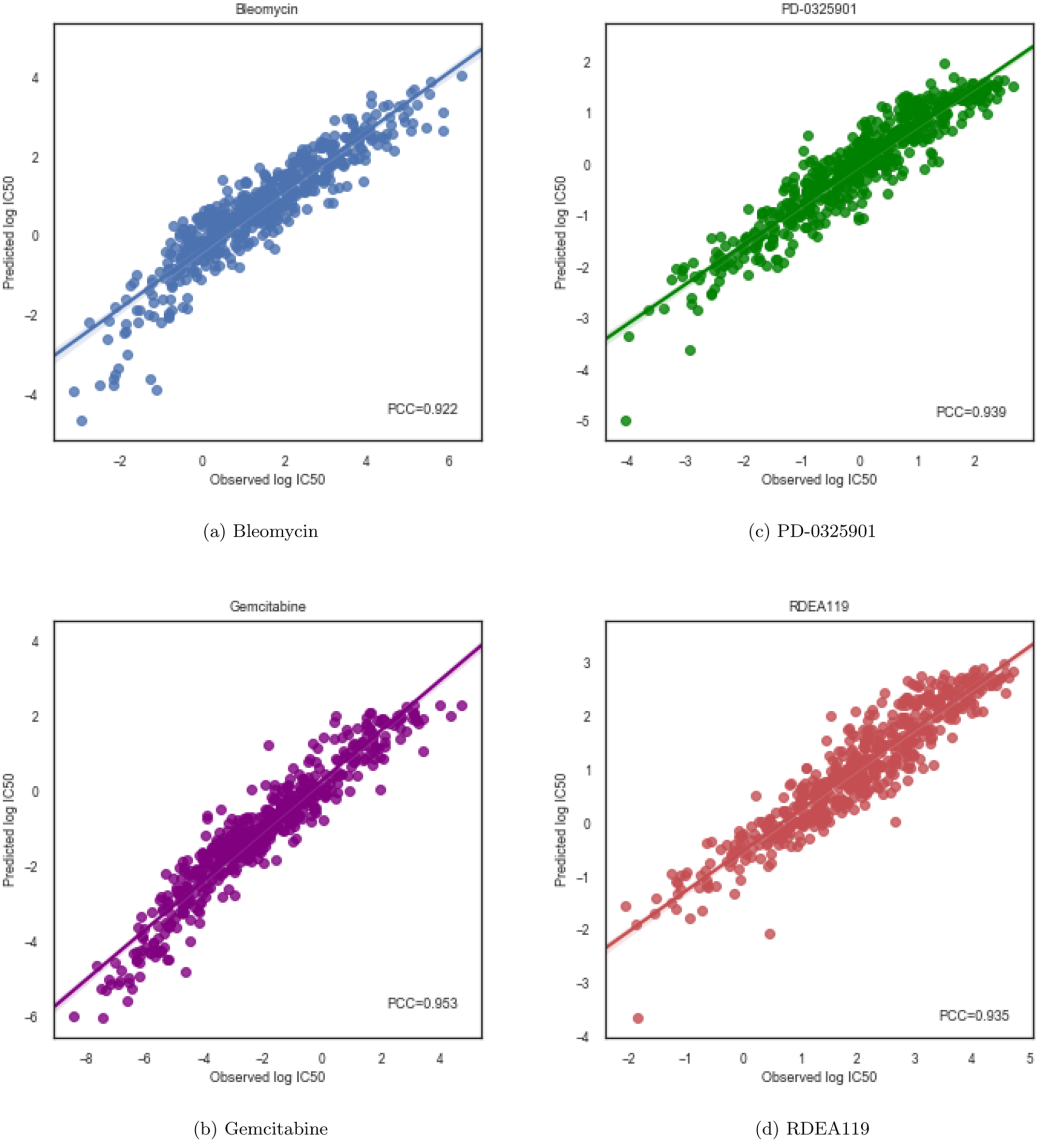
Drug-wise *PCC* for 4 drugs in GDSC. The computed *PCC* is illustrated in lower right corner of each plot.

### Comparison of prediction performance with state-of-the-art methods

For comprehensive evaluation of ADRML’s performance, we compared it to other recent state-of-the-art methods, namely, CDRscan^21^, CDCN^25^, SRMF^1^, and CaDRReS^19^. The implementations of all methods were obtained from the available codes referred to in their papers. In order to have a fair comparison, we conducted all evaluations in the same setting and using the same datasets. The comparison was made on the average performance of the models over 30 repetitions of fivefold cross-validation with tuned hyper-parameters.

It should be noted that the hyper-parameters of CaDRReS cannot be fully tuned, due to its high time complexity. The hyper-parameters for CaDRReS is assumed according to its paper and authors’ suggestion.

The features used for cell lines and drugs are different in these methods. For each method, the required features, as mentioned in their paper, are provided from the benchmark datasets described in "Benchmark datasets and collected features".

In addition to the mentioned methods, K-nearest neighbor (KNN) with $K=1$ was considered as a baseline method and compared to the results of other methods. KNN is implemented using the Scikit-learn module in Python^36^. For executing KNN, the input feature vector for each pair of cell line $c_i$ and drug $d_j$ was considered as the concatenated vector of *i*th row of *simC* and *j*th column of *simD*. All types of cell line similarities and drug similarities were considered as *simC* and *simD*, respectively. The complete report of KNN performance on various types of similarities are provided in Supplementary Table S1. KNN obtained the best performance on gene expression similarity of cell lines and chemical substructure similarity of drugs.

Tables 3 and 4 present the performance of the mentioned methods on CCLE and GDSC, respectively. Additionally, the scatter-plots with fitted lines for the predictions of the mentioned methods on CCLE are presented in Supplementary Figs. S123–S128.

**Table 3:** Comparison of methods’ performance on CCLE dataset.

Method	Cell line similarity	Drug similarity	RMSE	$$ {R^2 }$$	PCC
ADRML	Gene expression	Target protein	0.49	**0**.**68**	**0**.**85**
CDRscan	Mutation	Chemical	0.76	0.67	0.83
CDCN	Gene expression	Chemical	0.48	0.67	0.83
SRMF	Gene expression	Chemical	**0**.**25**	0.40	0.80
CaDRReS	Gene expression	–	0.53	0.31	0.52
KNN	Gene expression	Chemical	0.56	0.57	0.78

The methods were evaluated by averaging over 30 repetitions of fivefold cross-validation on cell line-drug pair. The best results of each criterion are shown in boldface.

The results of baseline method (KNN) in both datasets were not too far from the state-of-the-art methods, which means that improving the results is challenging. In CCLE dataset, SRMF achieved the best *RMSE* and favorable *PCC*; however, it achieved $R^2$ lower than the baseline, i.e., the variance of predicted $\log IC50$ did not explain the variance of real drug responses perfectly. CaDRReS yielded reasonable results but its $R^2$ and *PCC* were less than the baseline. CDRscan obtained the favorable $R^2$ and *PCC* but it had the highest *RMSE*. Therefore, its prediction values have a high correlation and far distance to the real responses, simultaneously. CDCN revealed a satisfying performance but with lower $R^2$ and *PCC*, and higher *RMSE* than the results of ADRML. Therefore ADRML outperformed other methods.

**Table 4:** Comparison of methods’ performance on the GDSC dataset.

Method	Cell line similarity	Drug similarity	RMSE	$$ {R^2 }$$	PCC
ADRML	Gene expression	Chemical	0.73	**0**.**75**	**0**.**88**
CDRscan	Mutation	Chemical	0.76	0.72	0.83
CDCN	Gene expression	Chemical	0.77	0.72	0.85
SRMF	Gene expression	Chemical	**0**.**20**	0.62	0.85
CaDRReS	Gene expression	–	0.49	0.34	0.57
KNN	Gene expression	Chemical	0.99	0.55	0.78

The methods were evaluated by averaging over 30 repetitions of using fivefold cross-validation on cell line-drug pair. The best result of each criterion is shown in boldface.

In the case of the GDSC dataset, SRMF obtained the best *RMSE* and moderate $$ R^2$$, and *PCC*. The performance of CaDRRS was satisfying, but $$ R^2 $$ and *PCC* were worse than the baseline. CDRscan showed good performance but with high *RMSE*, similar to its performance on the CCLE dataset. Moreover, CDCN’s performance was satisfying; however, its $$ R^2 $$ and *PCC* were lower than ADRML, and its *RMSE* was higher than ADRML. Consequently, ADRML outperformed other methods with regard to $$ R^2 $$, and *PCC*.

In addition to the mentioned analysis, we investigated whether using other types of cell line similarities and dug similarities would aid in improving the results of other methods. To this aim, we executed CDCN, SRMF, CaDRReS, and KNN on all types of similarities. It is worth mentioning that CDRscan receives binary feature matrices as the input and the dimension of binary feature vectors of drugs in CCLE and GDSC datasets were not appropriate for the designed CNNs in CDRscan; therefore, it is not applicable to perform CDRscan on other types of similarities. Other methods (CDCN, SRMF, CaDRReS, and KNN) receive the similarity matrices as the input. Moreover, CaDRReS gets only the cell line similarity, and it does not obtain any drug similarity matrix from the input.

The entire report of the performance criteria measured for the performance of the mentioned methods is presented in Supplementary Table S1. It can be seen that the performance of other methods almost does not improve using other similarities in comparison to their proposed similarities. Often, with respect to a particular pair of cell line similarities and drug similarities, SRMF obtains the best *RMSE*. At the same time, ADRML achieves best $R^2$ and best *PCC*.

All in sum, ADRML performed better than other state-of-the-art methods on both CCLE and GDSC in terms of $R^2$ and *PCC*. These achievements further substantiate ADRML performance.

**Figure 4:**
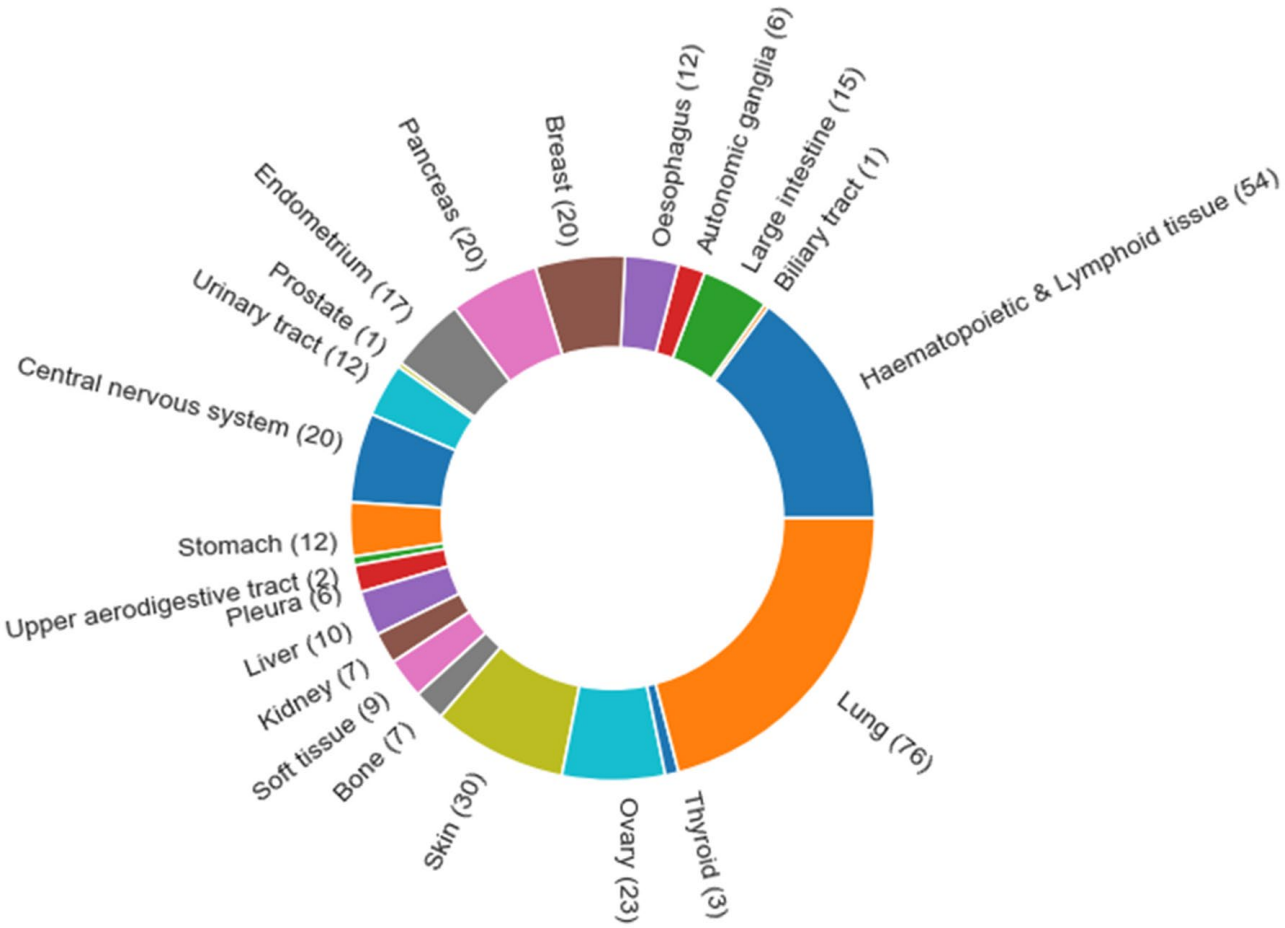
Tissue types in CCLE. The number of cell lines in each tissue type is shown in parenthesis.

### Removing redundant cell lines from CCLE and GDSC

CCLE dataset contains 363 cell lines from 22 different tissue types. The number of cell lines in each tissue type is shown in Fig. 4. The least frequent tissue types (Biliary tract and prostate) contain one cell line, and the most frequent tissue type (Lung) comprises 76 cell lines. Since the cell lines from the same tissue may have high similarity, this may lead to redundancy. Thus, it is better to eliminate the redundancy within each tissue type and based on the number of cell lines from that tissue. In order to remove the redundancy in each tissue type, we filtered out the cell lines that are very similar to other cell lines. In this way, we excluded the cell lines with high similarity to other cell lines in the same tissue type.

The detailed procedure of removing redundant cell lines is described in “Finding the most redundant cell lines”. This procedure led to eliminating 64 cell lines and the remaining 299 cell lines from CCLE. The remaining cell lines comprise the purified CCLE dataset without redundancy. The list of remaining and excluding cell lines are reported in Supplementary Table S2. To analyze the performance of ADRML and other state-of-the-art methods on the new dataset, we executed these methods using 30 repetitions of fivefold cross-validation. Table 5 demonstrates the performance of methods on the new dataset. It can be seen that ADRML outperforms other methods with respect to $R^2$ and *PCC*.

**Table 5:** Comparison of methods’ performance on the purified CCLE dataset.

Method	Cell line similarity	Drug similarity	RMSE	$$ {R^2 }$$	PCC
ADRML	Gene expression	Target protein	0.60	**0**.**56**	**0**.**83**
CDRscan	Mutation	Chemical	0.89	0.54	0.79
CDCN	Gene expression	Chemical	0.65	0.43	**0**.**83**
SRMF	Gene expression	Chemical	**0**.**28**	0.26	0.74
CaDRReS	Gene expression	–	0.52	0.32	0.51
KNN	Gene expression	Chemical	0.57	0.55	0.75

The methods were evaluated by averaging over 30 repetitions of fivefold cross-validation on cell line-drug pair. The best result of each criterion is shown in boldface.

**Figure 5:**
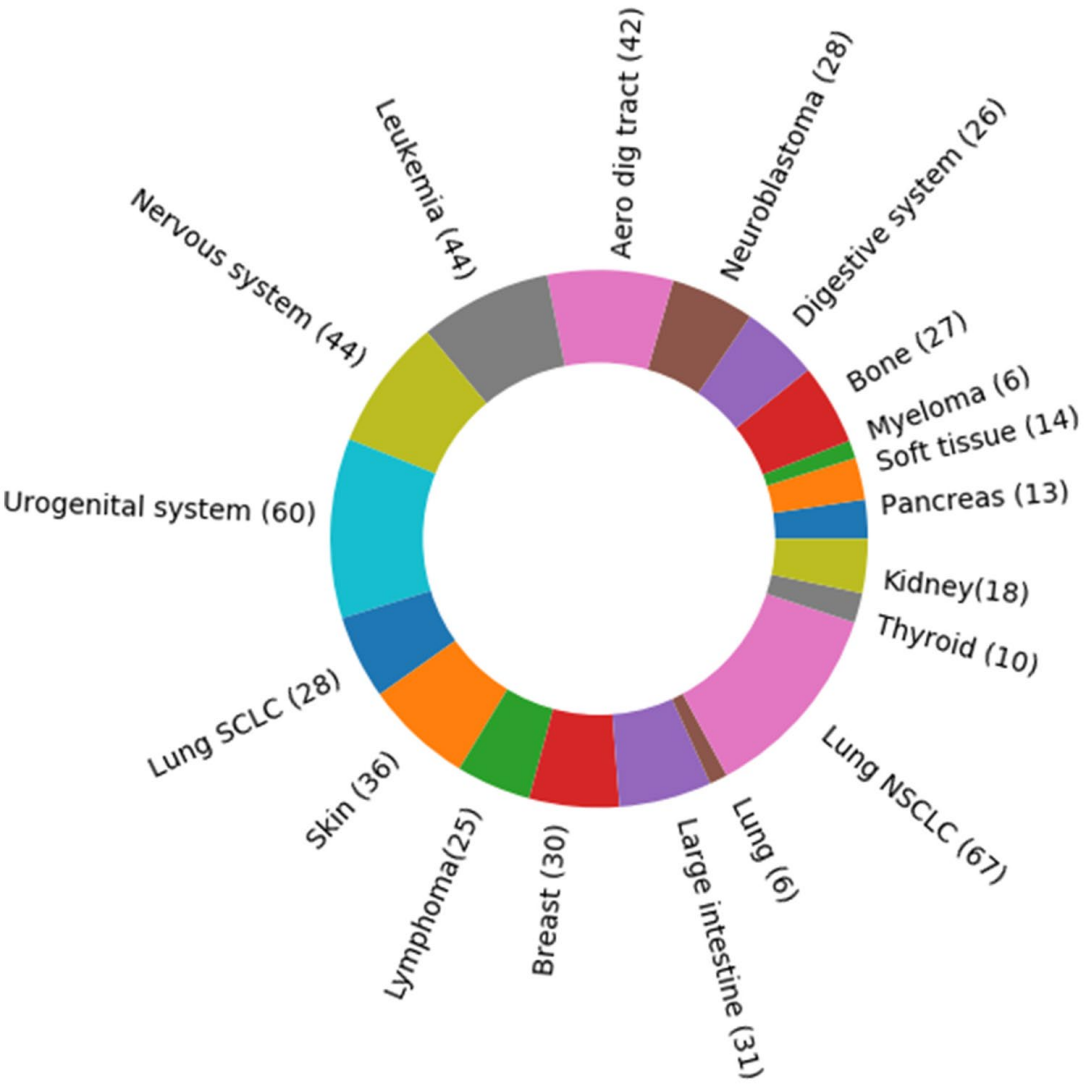
Tissue types in GDSC. The number of cell lines in each tissue type is shown in parenthesis.

Moreover, the GDSC dataset comprises 555 cell lines from 19 tissue types. Various tissue types have different numbers of cell lines which are shown in Fig. 5 To remove the redundant cell lines from GDSC, the procedure described in “Finding the most redundant cell lines” was applied on the GDSC, resulting in eliminating 103 cell lines and preserving 452 cell lines. The remaining cell lines form the purified GDSC dataset with lower redundancy. The list of remaining and excluding cell lines are reported in Supplementary Table S2. The performance of methods on the new GDSC dataset using 30 repetitions of fivefold cross-validation is represented in Table 6. It can be seen that SRMF obtained the best *RMSE*, CDCN achieved the best $R^2$ and ADRML yield the best *PCC*.

**Table 6:** Comparison of methods’ performance on the purified GDSC dataset.

Method	Cell line similarity	Drug similarity	RMSE	$$ {R^2 }$$	PCC
ADRML	Gene expression	Target protein	1.3	0.21	**0**.**85**
CDRscan	Mutation	Chemical	0.92	0.51	0.76
CDCN	Gene expression	Chemical	0.98	**0.55**	0.82
SRMF	Gene expression	Chemical	**0**.**54**	-33.11	0.70
CaDRReS	Gene expression	–	0.95	0.29	0.52
KNN	Gene expression	Chemical	1.00	0.54	0.77

The methods were evaluated by averaging over 30 repetitions of fivefold cross-validation on cell line-drug pair. The best result of each criterion is shown in boldface.

It can be inferred from the comparison results in Tables 3 and 4 with the results in the Tables 5 and 6 that the performance of models declines a bit when the redundant cell lines were removed. This issue may be due to the reduction in sample size or the existence of bias before removing redundancy of cell lines.

Moreover, we applied the redundancy removal procedure with different thresholds $(\theta )$ to investigate the performance of ADRML on different levels of redundancy removal. Furthermore, this procedure is repeated based on gene expression similarities of cell lines. Table 7 represents the number of remaining cell lines according to the various values of threshold.

**Table 7:** The number of remaining cell lines in CCLE and GDSC after applying redundancy removal procedure with different levels of strictness (threshold) based on copy number variation and gene expression.

Threshold	Dataset	Remained cell lines based on copy number variation	Remained cell lines based on gene expression
0.01	CCLE	72	111
0.05	CCLE	116	161
0.1	CCLE	194	209
0.15	CCLE	245	245
0.2	CCLE	299	277
0.25	CCLE	325	303
0.3	CCLE	339	322
0.35	CCLE	351	345
0.01	GDSC	78	107
0.05	GDSC	161	209
0.1	GDSC	266	293
0.15	GDSC	363	362
0.2	GDSC	452	435
0.25	GDSC	501	480
0.3	GDSC	529	509
0.35	GDSC	547	532

**Figure 6:**
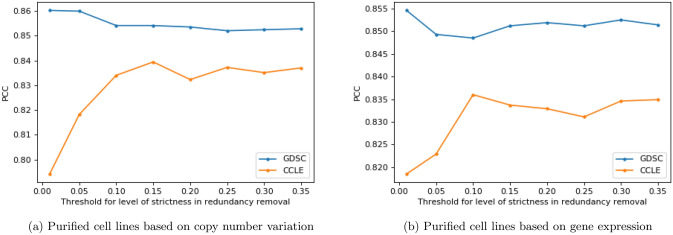
Performance of ADRML on purified datasets with different levels of strictness. The purified datasets were obtained after redundancy removal with certain thresholds. Each panel shows the *PCC* of ADRML assessed using 5-fold cross-validation on the purified datasets.

ADRML performance was evaluated on each of the resulting datasets after redundancy removal based on various levels of strictness. Figure 6a,b illustrate the *PCC* values of ADRML assessed using 5-fold cross-validation on the purified datasets. These figures verify that the trend of ADRML performance is almost the same on purified datasets based on copy number variation and gene expression. ADRML achieves the best *PCC* on the strictest threshold which removes a lot of cell lines and adding other cell lines declines its *PCC*. Moreover, the ADRML’s *PCC* on purified CCLE datasets first increased sharply and then decreased by lowering the level of strictness in redundancy removal.

### Analysis of association between drugs and signaling pathways

**Figure 7:**
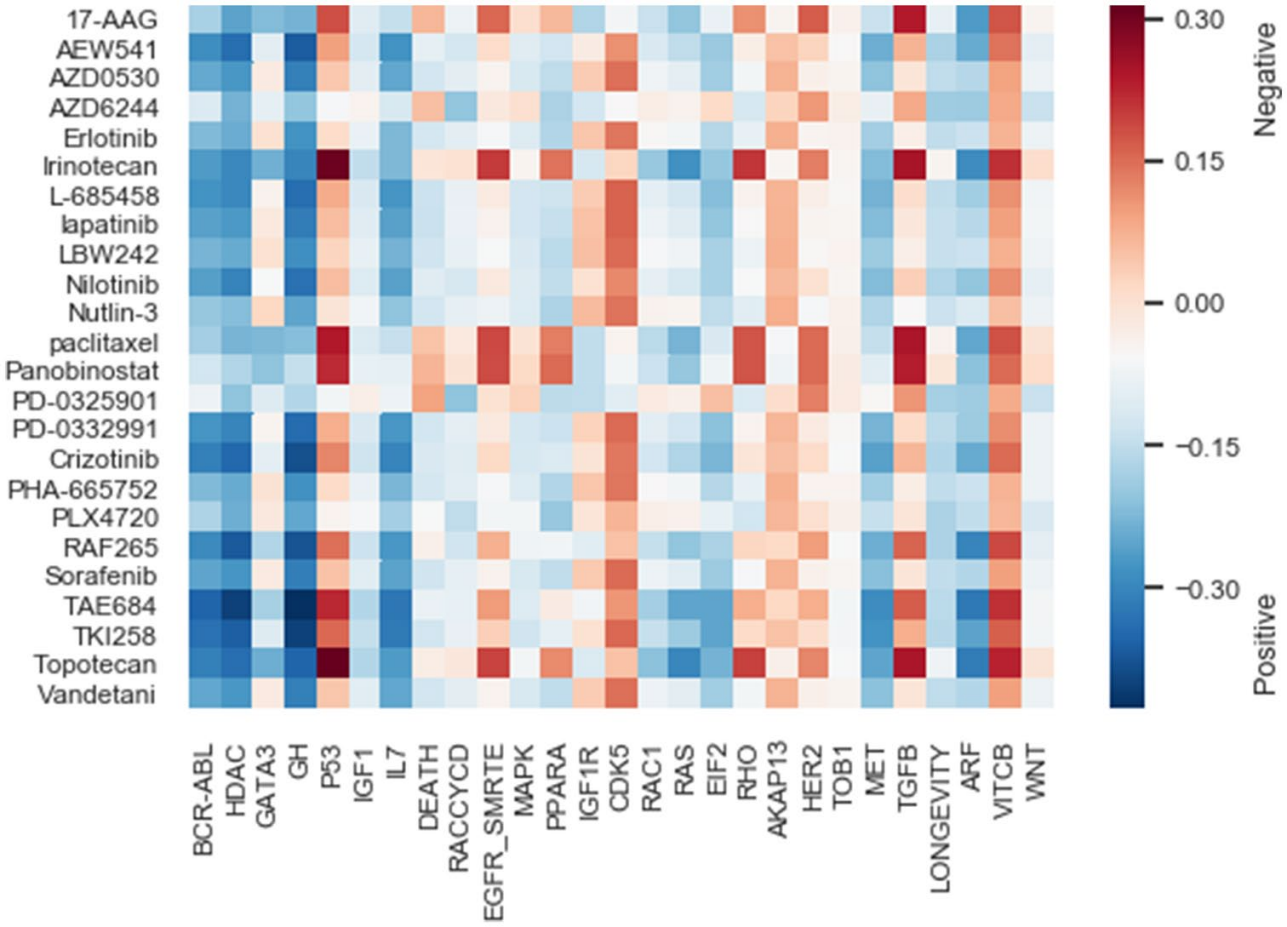
Correlation of pathway activity scores and drug responses. The drugs are shown in rows, and pathways are shown in columns. The positive correlations are represented in red and negative correlations are represented in blue. The intensity of the color indicates the extent of correlation.

To demonstrate that the prediction of ADRML is meaningful and rational, we investigated the correlation between the predicted drug responses and pathway activity scores for several Biocarta Pathways from MsigDB^37^. The detailed procedure is described in “Computing association of drugs and signalling pathways”. Figure 7 visualizes the association between drugs and signaling pathways for 24 drugs in the CCLE dataset and 25 Biocarta pathways. The entire association values are provided in Supplementary Table S3. There are numerous pieces of evidence in the literature for these correlations, some of which are provided here.

*Paclitaxel* drug and $TGF\beta$ signaling pathway exhibited a highly positive correlation. *Paclitaxel* is one of the agents that have been frequently reported for the activation of $TGF\beta$ pathway^38–41^. Thus, the higher consumption of *Paclitaxel* leads to more activation of $TGF\beta$, which verifies the high positive correlation between *Paclitaxel* and $TGF\beta$. Moreover, *Paclitaxel* positively associated with *P*53 pathway. It has been verified that *Paclitaxel* activates *P*53 signaling pathway^42^ and the cell lines with disrupted *P*53 are resistant to *Paclitaxel*^43^. Furthermore, *HSP*90 inhibitor $17-AAG$ was positively correlated with *P*53 pathway. It has been suggested in the previous studies that $17-AAG$ has an anti-tumor activity via activation and stabilization of *P*53^44^, that admits the positive association of $17-AAG$ and *P*53 pathway.

*Irinotecan* response has a very significant positive correlation with the activity score of *P*53. *Irinotecan* is a topoisomerase I inhibitor, which is frequently used for anticancer therapy. The previous study on human hepatocellular carcinoma (HCC) cell lines for the investigation of the apoptotic mechanisms of *Irinotecan* has revealed that it significantly activates *P*53^45^. Additionally, the positive correlation of *Irinotecan* response and *EGFR* pathway is supported by several pieces of research. They have shown the resistance to *Irinotecan* is connected with the increased expression of *EGFR*^46^ and have admitted that *Irinotecan* upregulates the *EGFR* pathway^47^. Also, *Panobbinostat* which is a potent inhibitor of deacetylases and HSP90^48^, revealed a high significant positive correlation with $TGF\beta$ pathway. Previous study have shown that using *Panobinostat* increased the level of $TGF\beta$^48^.

**Table 8:** The Literature evidence for some of sensitive predictions of ADRML about novel cell line-drug pairs.

Cell line	Drug	Cell line cancer type	Drug indication	Evidence
HOP-92	Parthenolide	Non-Small Cell Lung Cancer	Pan-cancer	Janganati et al.^49^
MDA-MB-468	Parthenolide	Breast cancer	Pan-cancer	Janganati et al.^49^
HCT-116	Parthenolide	Colon cancer	Pan-cancer	Janganati et al.^49^
MKN45	Roscovitine	Gastric adenocarcinoma	Pan-cancer	Trenti et al.^50^
AGS	Roscovitine	Gastric adenocarcinoma	Pan-cancer	Trenti et al.^50^

These cell line-drug pairs has unknown IC50 in the training dataset and ADRML predicted them as the sensitive prediction. The evidence papers for these predictions are represented in the last column.

**Table 9:** ADRML sensitive predictions for novel cell line-drug pairs verified by the latest release of GDSC.

Cell line	Drug	Cell line cancer type	Drug indication
SK-MEL-24	NSC-87877	Melanoma	Melanoma
SK-MEL-3	NSC-87877	Melanoma	Melanoma
TK10	Erlotinib	Renal cell carcinoma	Non-small cell lung cancer (NSCLC) and pancreatic cancer
SW684	WH-4-023	Fibrosarcoma	Pan-cancer
SW982	BMS-509744	Synovial sarcoma	Pan-cancer
SK-LMS-1	CMK	Vulvar leiomyosarcoma	Pan-cancer
SW982	A-770041	Synovial sarcoma	Sarcoma

These pairs had unknown IC50 in the training dataset and were predicted as a sensitive pair by ADRML. The latest release of GDSC reported these pairs as the sensitive pairs.

### Case studies

We conducted case studies on GDSC cell-line-drug pairs with unknown IC50 values. To do this, we did not impute the missing values in the *IC*50 matrix and trained ADRML with all known drug responses. For each drug, the predictions of ADRML on unknown pairs were partitioned into four quantiles, and the cell lines in the first and last quantiles were considered as the sensitive and resistant cell lines for that drug, respectively. The complete list of sensitive and resistant predicted associations are provided in Supplementary Tables S4 and S5, respectively. The sensitive associations were inquired into both the literature and the latest release of GDSC (released Feb. 2020). Table 8 represents the supportive pieces of evidence for ADRML predictions in Literature. Table 9 incorporates some of the cell line-drug pairs that had unknown *IC*50 values in the previous data extracted from GDSC, and now the drug response value for these pairs are available in the latest release of GDSC.

## Discussion

In this study, we proposed a computational model for predicting anticancer drug response, using manifold learning, called ADRML. The model combines three sets of information, including known drug responses, cell line similarity, and drug similarity, to infer the novel predictions. The main contribution of this paper is evaluating the influence of various types of cell line similarities and drug similarities on the prediction performance. We collected various features for cell lines and drugs from CCLE, GDSC, STiTCH, PubChem, and Drugbank. Here, we investigated nine different scenarios using three cell line similarities based on gene expression, mutation, and copy number variation, and three drug similarities based on the chemical substructure, target proteins, and KEGG pathways. The performance of ADRML was investigated using fivefold cross-validation on cell line-drug pairs. The best performance was obtained using gene expression data about cell lines and target protein data about drugs, which was more accurate than the previously proposed methods. We also investigated the performance of other state-of-the-art methods and KNN (with k = 1) as the baseline method on various types of similarities and showed that their best performance was achieved using the similarities that were suggested in their papers.

Another contribution of this paper was the purification of CCLE and GDSC benchmarks via removing redundant cell lines. The purified benchmarks were also used for assessing the methods’ performance. The results showed that excluding redundant cell lines declines the methods’ performance, which may be due to the reduction of sample size or removing bias from the database.

It was interesting that KNN with k = 1 as a simple baseline method shows favorable results and outperforms some more complicated methods, especially on the purified datasets. However, it should be noted that sophisticated methods’ performance declines when the data size is not sufficient. A complicated method needs a massive amount of data to train well and gets a good grasp of predicting outputs from inputs. For example, Chang et al.^21^ have provided CDRscan with more cell lines and drugs than used in this paper and have trained CDRscan with 95% of its data (despite 80% of data in this paper). Therefore, the reported $R^2$ in^21^ is better than the results reported in this paper. One can conclude that providing more informative data may enrich the training data and lead to better training the complex models. It is noteworthy that due to the challenging inherent of the problem, little improvements in results is welcome and useful.

The proposed method in this study outperformed other methods in terms of two criteria $R^2$ and *PCC* in most comparison scenarios. The predicted drug response values revealed high correlations with observed drug responses and suggested meaningful clues about drug mechanisms in activation/inhibition of pathways. Moreover, the reliable literature evidence supports the predictions of ADRML about novel cell line-drug pairs. As a consequence, the promising results of ADRML verified its efficiency in predicting anticancer drug prediction and imputation.

## Method

The proposed method includes five steps:

- Pre-processing to impute missing data
- Calculating various types of similarity matrices for cell lines and drugs
- Normalizing the similarity matrices
- Similarity-constrained manifold learning to factorize the IC50 matrix into low-rank latent matrices
- Estimating Unknown IC50 values using the latent matrices

**Figure 8:**
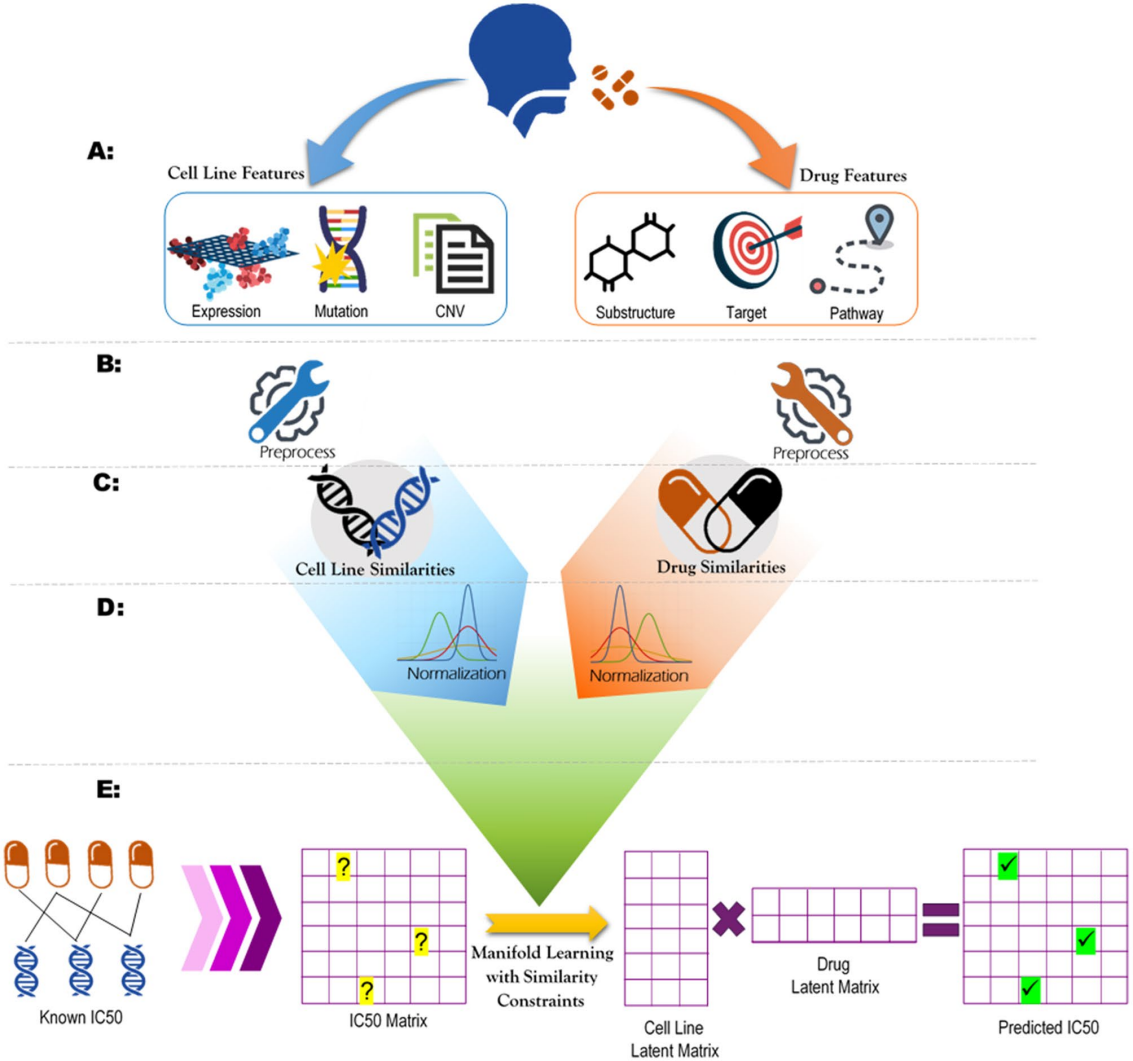
The overall workflow of ADRML. (**A**) Collecting various types of cell lines and drugs features. Further steps can be executed for each pair of cell line feature types and drug feature types. (**B**) Pre-processing the collected feature by removing the features with missing data for more than half samples and then imputing the remaining missing values. (**C**) Computing various types of cell line similarities and drug similarities using similarity functions. (**D**) Normalizing the similarity matrices using symmetric normalized Laplacian. (**E**) The IC50 matrix constructed from known IC50 values is factorized into two low-rank latent matrices with constraints of the similarity matrices. The unknown IC50 values can be predicted by multiplying the latent matrices.

The overall workflow of ADRML is illustrated in Fig. 8.

For the convenience, define $$ EXPR_{c_i} $$, $$ CNV_{c_i} $$, and $$ MUT_{c_i} $$ as the expression of all genes, copy number variation $$ c_i $$, respectively. More precisely, $$ CNV_{c,g} $$ and $$ MUT_{c,g}$$ denote the copy number variation and mutation status of gene *g* in cell line *c*. Furthermore, $$ CHEM_{d_i} $$, $$ TRGT_{d_i} $$, and $$ KEGG_{d_i} $$ stand for chemical features, target status (equals 1 for the proteins that are the target of the drug, 0 otherwise) for all proteins, and pathway status (equals 1 for the pathways that are the associated with the drug, 0 otherwise) for drug $$ d_i $$, respectively. Finally, $$ IC50_{c_i,d_j} $$ is defined as the $$\log IC50 $$ value for cell line $$ c_i $$ treated with drug $$ d_j $$.

### Pre-processing to impute the missing data

Several steps were done to impute the missing data. First, the features that were missed in the majority of cell lines are removed. Second, the cell lines that contain missing values for more than half of the features were excluded. The other missing values were imputed using a k-nearest neighbor approach. To this aim, the distance measure between cell lines was defined as the Euclidean distance of their expression profiles because there is no missing in expression features of the cell line; thus, the distance can be calculated for each pair of cell lines. The distance between $c_1$, $c_2$ is $D(c_1,c_2)=||EXPR_{c_1} - EXPR_{c_2} ||_{2}^{2}$. Then, the mean feature value among 10-nearest cell lines was used to impute the missing *IC*50 value of drug *d* or *CNV* value of gene *g* in cell line *c*.

2 $$\begin{aligned} IC50_{c,d}&=\sum _{c_i \in NN_c}\alpha _i IC50_{c_i,d} \end{aligned}$$

3 $$\begin{aligned} CNV_{c,g}&=\sum _{c_i \in NN_c}\alpha _i CNV_{c_i,g} \end{aligned}$$

where $NN_c$ is the set of 10 cell lines with the minimum distance from cell line *c*, and $\alpha _i=\dfrac{D(c,c_i)}{\sum _{c_j \in NN_c}D(c,c_j)}$. Moreover, to impute the mutation status (“1” for mutated and “0” for wild type) of gene *g* in cell line *c*, the majority vote of 10 nearest cell lines is used, i.e. $MUT_{c,g}$ is 1, if and only if $\sum _{c_i \in NN_c}MUT_{c_i,g}>\sum _{c_i \in NN_c}(1-MUT_{c_i,g})$.

### Similarity matrices construction and normalization

For computing the similarity score of two cell lines (or drugs), the *PCC* and Jaccard-index (JI) were regarded as the similarity function, which are elaborated in the following.

4 $$\begin{aligned} PCC(x,y)&=\dfrac{\sum _i (x_i-{\bar{x}})(y_i-{\bar{y}})}{\sqrt{\sum _i (x_i-{\bar{x}})^2}\sqrt{\sum _i (y_i-{\bar{y}})^2}} \end{aligned}$$

5 $$\begin{aligned} JI(x,y)&=\dfrac{\sum _i (x_i y_i)}{\sum _i (x_i+y_i) -\sum _i (x_i y_i)} \end{aligned}$$

where $$ x,\ y $$ are two feature vectors, $$ x_i$$ and $$ y_i $$ denote the *i*th element of these vectors, and $$ {\bar{x}},\ {\bar{y}} $$ are the mean value of them. Basically, the *PCC* is used to calculate the similarity of two continuous vectors, while *JI* is appropriate to measure the similarity of two discrete vectors. Therefore, we considered this rationality in the calculation of similarity matrices. The dimensions of cell line, and drug similarity matrices are $$ n\times n $$ and $$ m\times m $$, respectively, where *n* denotes the number of cell lines and *m* denotes the number of drugs. Consequently, we constructed the three types of similarity matrices for cell lines, based on *EXPR*, *CNV*, and *MUT*. Since *EXPR* and *CNV* features are real-valued, *PCC* was used to measure their similarity, while *MUT* is binary-valued and *JI* was used to measure mutation similarity.

- $SimC_{EXPR}$ is the similarity matrix of cell lines based on their gene expression profiles.
  6 $$\begin{aligned} SimC_{EXPR}(c_i,c_j) =PCC(EXPR_{c_i},EXPR_{c_j}) \end{aligned}$$
- $SimC_{CNV}$ is the similarity matrix of cell lines based on their copy number variations.
  7 $$\begin{aligned} SimC_{CNV}(c_i,c_j) =PCC(CNV_{c_i},CNV_{c_j}) \end{aligned}$$
- $SimC_{MUT}$ is the similarity matrix of cell lines based on their mutation profiles.
  8 $$\begin{aligned} SimC_{MUT}(c_i,c_j) =JI(MUT_{c_i},MUT_{c_j}) \end{aligned}$$

Furthermore, three types of similarity matrices for drug based on Pubchem SMILES (*CHEM*), target proteins (*TRGT*), and KEGG pathways (*KEGG*) were calculated as follows. It is notable that all drug features are binary-valued; thus, *JI* was used for measuring the similarity of drugs based on each type of information.

- $SimD_{CHEM}$ is the similarity matrix of drugs according to their chemical substructure fingerprints.
  9 $$\begin{aligned} SimD_{CHEM}(d_i,d_j) =JI(CHEM_{d_i},CHEM_{d_j}) \end{aligned}$$
- $SimD_{TRGT}$ is the similarity matrix of drugs according to their target proteins.
  10 $$\begin{aligned} SimD_{TRGT}(d_i,d_j) =JI(TRGT_{d_i},TRGT_{d_j}) \end{aligned}$$
- $SimD_{KEGG}$ is the similarity matrix of drugs according to their KEGG pathways.
  11 $$\begin{aligned} SimD_{KEGG}(d_i,d_j) =JI(KEGG_{d_i},KEGG_{d_j}) \end{aligned}$$

Then all of the computed similarity matrices were normalized by computing the symmetric normalized Laplacian^51^. Let *S* be a similarity matrix, the normalized similarity matrix $S^{norm}$ was obtained as follows.

12 $$\begin{aligned} S^{norm}&=D^{-1/2}LD^{-1/2} \end{aligned}$$

13 $$\begin{aligned} L&=D-A \end{aligned}$$

where *D* is a diagonal matrix with diagonal elements equal to the summation of each row in *S*, i.e. $D_{i,i} =\sum _j S_{i,j}$. It is noteworthy that $D_{ii}\ne 0$.

### Manifold learning with similarity constraints

We constructed a bipartite graph with two parts: drugs and cell lines. The weight of edges between cell line $c_i$ and drug $d_j$ is $\log IC50$ value of drug $d_j$ on cell line $c_i$. Thus, the *IC*50 drug response matrix $R=[r_{i,j}]_{n\times m}$ is the adjacency matrix of this graph, where $n, \ m$ are the number of cell lines and drugs, respectively. We used the manifold learning to factorize the drug response matrix *R* in two latent matrices $P_{n\times k}$ and $Q_{m\times k}$ with lower rank. By using this factorization we could map the cell line and drug features into a latent space with dimension *k*, i.e. *P* and *Q* are the cell line latent matrix and drug latent matrix, respectively. The *i*th row of *P* (shown by $p_i$) is the latent vector of cell line $c_i$, and the *j*th row of *Q* (shown by $q_j$) indicates the latent vector of drug $d_j$.

The initial goal is to find matrices *P* and *Q*, such that each drug response value is obtained by inner product of corresponding latent vectors, i.e., $r_{i,j}=p_i\cdot q_j^T$; thus, the loss function can be formulated as:

14 $$\begin{aligned} L=\dfrac{1}{2} \sum _{i,j}(r_{i,j}-p_i\cdot q_j^T)^2+\dfrac{\mu }{2}\left( \sum _i ||p_i||^2+\sum _j ||q_j||^2\right) \end{aligned}$$

Two terms $\sum _i ||p_i||^2$ and $\sum _j ||q_j||^2$ are the regularization constraints of *P* and *Q* and $\mu$ is the regularization coefficient. The regularization terms prevent these matrices to grow dramatically; therefore, the over-fitting issue may not occur. These regularization terms help to reduce the variance and increase the stability and generalization capabilities of the model^52^.

Manifold learning studies^53,54^ have shown that the mapping of data to a lower dimensional space can conserve the topological structure of data. Since $$ p_i $$ is the feature vector of cell line $$ c_i $$, the distance of two cell lines $$ c_i$$ and $$ c_j $$ can be measured by $$ ||p_i-p_j ||^2$$. Similarly, $$ ||q_i-q_j||^2 $$ denotes the distance of drugs $$ d_i $$ and $$ d_j $$. We should consider some constraints to maintain the distance of cell lines and the distance of drugs while mapping them from the original features space to the lower dimensional latent space. Thus, the loss function is supplemented by two more terms.

15 $$\begin{aligned} L= & {} \dfrac{1}{2} \sum _{i,j}(r_{i,j}-p_i\cdot q_j^T)^2+\dfrac{\mu }{2} \left( \sum _i ||p_i||^2+\sum _j ||q_j||^2\right) \nonumber \\&+\dfrac{\lambda }{2} \left( \sum _{i,j}||p_i-p_j ||^2 SimC(i,j)+\sum _{i,j}||q_i-q_j||^2 SimD(i,j)\right) \end{aligned}$$

where $$ \lambda $$ is the coefficient of similarity consistency, $$ SimC\in \{SimC_{EXPR}$$, $$SimC_{CNV}$$, $$SimC_{MUT}\} $$, and $$ SimD\in \{SimD_{CHEM}$$, $$ SimD_{TRGT}$$, $$SimD_{KEGG}\} $$. Two last terms are minimized when the feature vectors of cell line (or drug) pairs with high similarity are mapped to not distant latent vectors. Therefore, the topological distance of cell lines (or drugs) is maintained while mapping to the lower dimensional space.

#### Iterative optimization rules

The latent matrices *P*, *Q* must be obtained by minimizing the loss function in 15. We used the iterative Newton’s method^55^ to update *P*, *Q* matrices:

16 $$\begin{aligned} p_i^{t+1}&=p_i^{t}-\nabla _{p_i} L(\nabla ^{2}_{p_i}L)^{-1} \end{aligned}$$

17 $$\begin{aligned} q_j^{t+1}&=q_j^{t}-\nabla _{q_j} L(\nabla ^{2}_{q_j}L)^{-1} \end{aligned}$$

where $p_i^{t}$ (or $q_j^{t}$) denotes the updated $p_i$ (or $q_j$) after t steps, for all $t>0$ and $p_i^{0}$, $q_j^{0}$ were initialized randomly. The first and second derivatives (gradient and Hessian) of loss function with respect to $p_i$ and $q_j$ are computed as the following:

18 $$\begin{aligned} \nabla _{p_i}L&=\sum _{i,j}(p_i\cdot q_j^T-r_{i,j})q_j+\mu p_i +\lambda \sum _j (p_i-p_j)SimC(i,j)-\lambda \sum _j(p_j-p_i)SimC(j,i) \end{aligned}$$

19 $$\begin{aligned} \nabla ^{2}_{p_i}L&=\sum _j q_j^{T}q_{j}+\mu I =\lambda \sum _j( SimC(i,j)+simC(j,i))I \end{aligned}$$

20 $$\begin{aligned} \nabla _{q_j}L&=\sum _{i,j}(q_j\cdot p_i^T-r_{i,j})p_i+\mu q_j +\lambda \sum _i (q_j-q_i)SimD(j,i)-\lambda \sum _i(q_i-q_j)SimD(i,j) \end{aligned}$$

21 $$\begin{aligned} \nabla ^{2}_{q_j}L&=\sum _i p_i^{T}p_i+\mu I =\lambda \sum _i (SimD(j,i)+simD(i,j))I \end{aligned}$$

Therefore, the latent matrices *P*, *Q* are updated alternatively according to Eqs. (22, 23) until convergence.

22 $$\begin{aligned} p_i^{t+1}&=\left[ \sum _j r_{i,j}q_{j}^{t}+\lambda \sum _j(SimC(i,j)+simC(j,i))p_i^t\right] \nonumber \\&\quad \left[ \sum _j q_j^{T}q_{j}+\mu I =\lambda \sum _j (SimC(i,j)+simC(j,i))I\right] ^{-1} \end{aligned}$$

23 $$\begin{aligned} q_j^{t+1}&=\left[ \sum _i r_{i,j}p_{i}^{t+1}+\lambda \sum _i(SimD(j,i)+simD(i,j))q_j^t\right] \nonumber \\&\quad \left[ \sum _j q_j^{T}q_{j}+\mu I =\lambda \sum _j (SimC(i,j)+simC(j,i))I\right] ^{-1} \end{aligned}$$

The convergence criterion is met when $||p^{t+1^{T}}Q^{t+1}-p^{t^T}Q^t||<\epsilon$. In this study, we considered $\epsilon =0.01$. The value of loss function declined in every iteration, due to the positive definite second derivatives. Therefore, the convergence criterion is definitely met after some steps^55^ (usually after 10–20 step). After convergence, an estimated matrix is obtained by $R_{pred}=Q*P^{T}$.

Moreover, the manifold learning was applied on the transpose of response matrix, i.e. all the above procedure was repeated for factorizing $$ R^T $$ to $$ P' $$ and $$ Q' $$ . In the second use of Manifold learning we initialized $$ P' $$ and $$ Q' $$ by the final computed *Q* and *P* in the first run, respectively. After the convergence, the second predicted matrix was constructed by $$ R'_{pred}= P'*Q'^{T}$$. Consequently, the predicted $$ \log IC50 $$ was computed by $$ {\hat{R}}=0.5(R_{pred}+R'_{pred}) $$.

### Evaluation criteria

**Figure 9:**
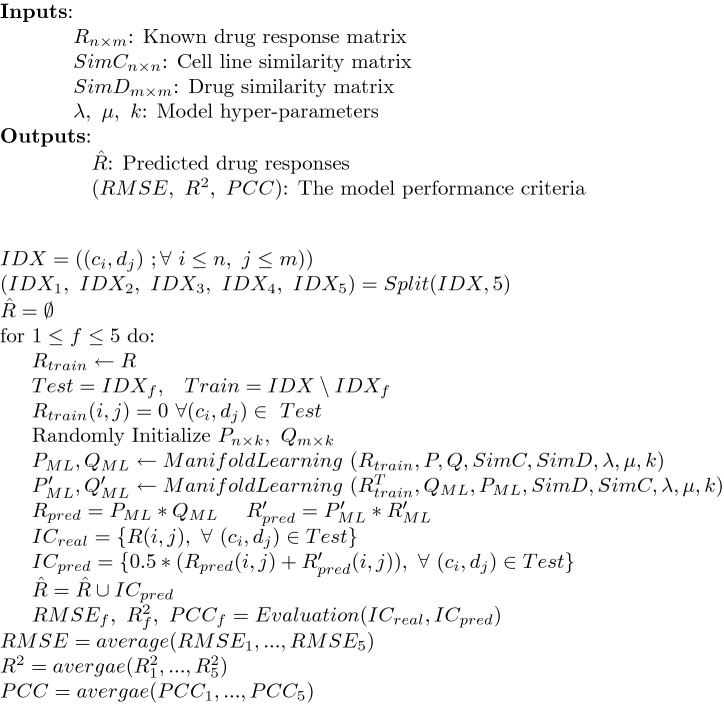
The pseudo-code for evaluation of ADRML performance.

We measured the performance ADRML using 5-fold cross-validation on cell line-drug pairs. To do this, each pair of $(c_i,d_j)$ was considered as a sample. Then, the set of all samples was partitioned randomly into five almost equally-sized subsets (fold). One fold was considered as the test data and the other folds were regarded as the training data. The evaluation was computed for the test data. This procedure was iterated until each fold was considered once as the test data. Finally, the average of evaluation criteria over these five iterations denoted the model performance. Evaluation of ADRML is summarized as pseudo-code and shown in Fig. 9.

To avoid randomness and reducing variance, the model performance was averaged over 30 randomly repetition of 5-fold cross-validation. The evaluation criteria include *RMSE*, $R^2$, and *PCC* as follows.

24 $$\begin{aligned} RMSE(IC_{real},IC_{pred})&=\sqrt{\dfrac{1}{|Test|}\sum _i \left( IC_{real}(i)-IC_{pred}(i)\right) ^2} \end{aligned}$$

25 $$\begin{aligned} R^2(IC_{real},IC_{pred})&=1-\dfrac{\sum _i\left( IC_{real}(i)-IC_{pred}(i)\right) ^2}{\sum _i\left( IC_{real}(i)-{\bar{IC}}_{real}\right) ^2} \end{aligned}$$

26 $$\begin{aligned} PCC(IC_{real},IC_{pred})&=\dfrac{\sum _i (IC_{real}(i)-{\bar{IC}}_{real})(IC_{pred}(i)-{\bar{IC}}_{pred})}{\sqrt{\sum _i (IC_{real}(i)-{\bar{IC}}_{real})^2}\sqrt{\sum _i (IC_{pred}(i)-{\bar{IC}}_{pred})^2}} \end{aligned}$$

where $$ IC_{real}$$ and $$IC_{pred} $$ are the vector of real and predicted drug response values for all samples in test set, respectively, $$ {\bar{IC}}_{real}, {\bar{IC}}_{pred} $$ are their mean values, and |*Test*| is the number of samples in the test set. Each criterion evaluates the model performance from a different point of view. Therefore, it is possible to obtain results which led to promising values of one criterion and unfavorable values for other criteria.

### Finding the most redundant cell lines

In order to eliminate the redundancy from the dataset, the cell lines in each tissue type that have high similarity to the majority of cell lines in that tissue type were considered as the most redundant cell lines and excluded from the dataset. To do this, the minimum (Q0), first quantile (Q1), second quantile (Q2), third quantile (Q3), and maximum (Q4) values for each type of cell line similarity in all tissue type were calculated, which are shown in Supplementary Tables S6 and S7. The diversity of cell lines was projected better concerning the values of copy number variation similarities, since there was a vast difference between the quantile values with respect to this similarity. Therefore, the third quantile of copy number variation similarities between the cell lines were computed in each tissue type *t* (denoted by *Q*3(*CNV*, *t*) ). The cell line *c* in tissue type *t* was excluded if it had the similarity higher than *Q*3(*CNV*, *t*) with more than $\theta =$20% of cell lines in tissue type *t*.

### Computing association of drugs and signalling pathways

The association between drug and pathway was computed by the *PCC* of drug response values and pathway activity scores. To do this, we considered all Biocarta signaling pathways and eliminated the pathways that the gene expression data of more than 10% of its genes were not provided. Therefore, we considered 107 Biocarta pathways for CCLE dataset. The pathway activity score for cell line $c_i$ and pathway $p_j$ was computed according to Emdadi *et. al.*^20^, by summing up the fold change of gene expressions for all genes $g_l$ in pathway $p_i$.

27 $$\begin{aligned} AS(c_i,p_j)=\sum _{g_l \in p_j} \log \dfrac{EXPR(c_i,g_l)}{median_c(EXPR(c,g_l))} \end{aligned}$$

where $median_c(EXPR(c,g_l))$ is the median of gene expression of gene $g_l$ in all cell lines. Thus, the score of a cell line in activating a pathway denotes the total amount of change in gene expression with respect to the median expression.

The correlation of drug $$ d_i $$ and pathway $$ p_j $$ was obtained by $$PCC(IC_{pred}(:,i),AS(:,j)) $$, where $$ IC_{pred}(:,i) $$ denotes the predicted drug response vector of drug $$ d_i $$ for all cell lines and *AS*( : , *j*) stands for the activity score vector of pathway $$p_j $$ for all cell lines.

## Supplementary information

Supplementary material 1

Supplementary material 2

## Data Availability

The data and implementation are accessible from (https://github.com/fahmadimoughari/ADRML).
