# Author Correction: ADRML: anticancer drug response prediction using manifold learning

**DOI:** 10.1038/s41598-020-77486-0

**Published:** 2020-12-15

**Authors:** Fatemeh Ahmadi Moughari, Changiz Eslahchi

**Affiliations:** 1 Department of Computer and Data Sciences, Faculty of Mathematical Sciences, Shahid Beheshti University, Tehran, Iran; 2 School of Biological Sciences, Institute for Research in Fundamental Sciences (IPM), Tehran, Iran

Correction to: *Scientific Reports* 10.1038/s41598-020-71257-7, published online 28 August 2020

In the original version of this Article, Fatemeh Ahmadi Moughari was incorrectly affiliated with “School of Biological Sciences, Institute for Research in Fundamental Sciences (IPM), Tehran, Iran”. The correct affiliation is listed below.

Department of Computer and Data Sciences, Faculty of Mathematical Sciences, Shahid Beheshti University, Tehran, Iran.

