# Supplementary material for: ADRML: anticancer drug response prediction using manifold learning
Source: Sci Rep. 2020 Aug 28;10:14245. doi: 10.1038/s41598-020-71257-7 (PMC7456328; doi:10.1038/s41598-020-71257-7)
Supplement: Supplementary file 1 — Supplementary material 1 [file 41598_2020_71257_MOESM1_ESM.pdf]

ADRML: Anticancer Drug Response Prediction Using Manifold Learning

Fatemeh Ahmadi Moughari, Changiz Eslahchi
Corresponding author: Changiz Eslahchi- Contact mail: ch-eslahchi@sbu.ac.ir

Supplementary Figures

List of Figures

1 *PCC* for Luminespib prediction 5
2 *PCC* for MK-2206 prediction 5
3 *PCC* for Methotrexate prediction 5
4 *PCC* for Midostaurin prediction 5
5 *PCC* for 17-AAG prediction 6
6 *PCC* for 14913-33-8 prediction 6
7 *PCC* for ABT-263 prediction 6
8 *PCC* for ABT-888 prediction 6
9 *PCC* for AP-24534 prediction 7
10 *PCC* for AS601245 prediction 7
11 *PCC* for ATRA prediction 7
12 *PCC* for Axitinib prediction 7
13 *PCC* for AZD-2281 prediction 8
14 *PCC* for AZD6244 prediction 8
15 *PCC* for AZD6482 prediction 8
16 *PCC* for AZD7762 prediction 8
17 *PCC* for AZD8055 prediction 9
18 *PCC* for BAY 61-3606 prediction 9
19 *PCC* for Bexarotene prediction 9
20 *PCC* for BIBW2992 prediction 9
21 *PCC* for Bicalutamide prediction 10
22 *PCC* for BIRB 0796 prediction 10
23 *PCC* for Bleomycin prediction 10
24 *PCC* for Bosutinib prediction 10
25 *PCC* for Bryostatin 1 prediction 11
26 *PCC* for CCT007093 prediction 11
27 *PCC* for CCT018159 prediction 11
28 *PCC* for CEP-701 prediction 11
29 *PCC* for CHIR-99021 prediction 12
30 *PCC* for Camptothecin prediction 12
31 *PCC* for Cytarabine prediction 12
32 *PCC* for DMOG prediction 12
33 *PCC* for Docetaxel prediction 13
34 *PCC* for Doxorubicin prediction 13
35 *PCC* for EHT 1864 prediction 13
36 *PCC* for Epothilone B prediction 13
37 *PCC* for FH535 prediction 14
38 *PCC* for FTI-277 prediction 14
39 *PCC* for GDC-0449 prediction 14
40 *PCC* for GDC-0449 prediction 14
41 *PCC* for GSK-1904529A prediction 15
42 *PCC* for GSK-650394 prediction 15
43 *PCC* for GW 441756 prediction 15
44 *PCC* for Gefitinib prediction 15
45 *PCC* for Gemcitabine prediction 16
46 *PCC* for IPA-3 prediction 16
47 *PCC* for JNJ-26854165 prediction 16
48 *PCC* for JNK Inhibitor VIII prediction 16
49 *PCC* for JNK-9L prediction 17
50 *PCC* for KU-55933 prediction 17
51 *PCC* for LAQ824 prediction 17
52 *PCC* for LFM-A13 prediction 17
53 *PCC* for Mitomycin-C prediction 18
54 *PCC* for NSC-87877 prediction 18
55 *PCC* for NU-7441 prediction 18
56 *PCC* for NVP-BEZ235 prediction 18
57 *PCC* for ACADESINE prediction 19
58 *PCC* for AG-014699 prediction 19

59 *PCC* for AKT inhibitor VIII prediction 19
60 *PCC* for AMG-706 prediction 19
61 *PCC* for Nilotinib prediction 20
62 *PCC* for Nutlin-3 prediction 20
63 *PCC* for OSI-906 prediction 20
64 *PCC* for OSU-03012 prediction 20
65 *PCC* for Obatoclax Mesylate prediction 21
66 *PCC* for PD-0325901 prediction 21
67 *PCC* for PD-0332991 prediction 21
68 *PCC* for PD173074 prediction 21
69 *PCC* for PD184352 prediction 22
70 *PCC* for PF-4708671 prediction 22
71 *PCC* for PF-562271 prediction 22
72 *PCC* for PLX4720 prediction 22
73 *PCC* for Pazopanib prediction 23
74 *PCC* for QS11 prediction 23
75 *PCC* for RDEA119 prediction 23
76 *PCC* for SB 216763 prediction 23
77 *PCC* for RO-3306 prediction 24
78 *PCC* for SB590885 prediction 24
79 *PCC* for SL 0101-1 prediction 24
80 *PCC* for TW 37 prediction 24
81 *PCC* for Tipifarnib prediction 25
82 *PCC* for VX-702 prediction 25
83 *PCC* for Vinblastine prediction 25
84 *PCC* for Vinorelbine prediction 25
85 *PCC* for Vorinostat prediction 26
86 *PCC* for ZINC46867 prediction 26
87 *PCC* for ZM-447439 prediction 26
88 *PCC* for embelin prediction 26
89 *PCC* for etoposide prediction 27
90 *PCC* for lenalidomide prediction 27
91 *PCC* for pac-1 prediction 27
92 *PCC* for shikonin prediction 27
93 *PCC* for temsirolimus prediction 28
94 *PCC* for thapsigargin prediction 28
95 *PCC* for BMS-708163 prediction 28
96 *PCC* for BMS-754807 prediction 28
97 *PCC* for BX-795 prediction 29
98 *PCC* for Elesclomol prediction 29
99 *PCC* for 17-AAG prediction 30
100 *PCC* for AEW541 prediction 30
101 *PCC* for AZD0530 prediction 30
102 *PCC* for AZD6244 prediction 30
103 *PCC* for Crizotinib prediction 31
104 *PCC* for Erlotinib prediction 31
105 *PCC* for Irinotecan prediction 31
106 *PCC* for L-685458 prediction 31
107 *PCC* for LBW242 prediction 32
108 *PCC* for Nilotinib prediction 32
109 *PCC* for Nutlin-3 prediction 32
110 *PCC* for PD-0325901 prediction 32
111 *PCC* for PD-0332991 prediction 33
112 *PCC* for PHA-665752 prediction 33
113 *PCC* for PLX4720 prediction 33
114 *PCC* for Panobinostat prediction 33
115 *PCC* for RAF265 prediction 34
116 *PCC* for Sorafenib prediction 34
117 *PCC* for TAE684 prediction 34
118 *PCC* for TKI258 prediction 34

119 *PCC* for Topotecan prediction . . . 35
120 *PCC* for Vandetani prediction . . . 35
121 *PCC* for lapatinib prediction . . . 35
122 *PCC* for paclitaxel prediction . . . 35
123 Scatterplot of ADRML's predictions. . . . 36
124 Scatterplot of CDCN's predictions. . . . 36
125 Scatterplot of SRMF's predictions. . . . 36
126 Scatterplot of CaDRReS' predictions. . . . 36
127 Scatter plot of CDRscan's predictions. . . . 37
128 Scatter plot of KNN's predictions. . . . 37

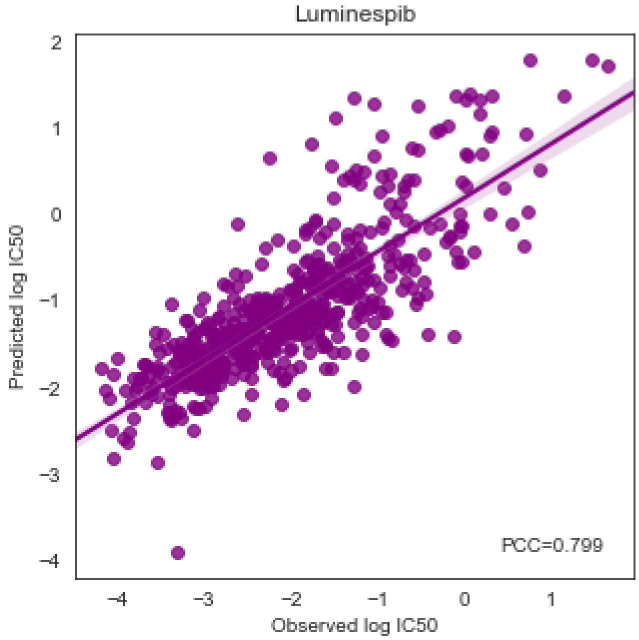

Supp. Fig. S 1: *PCC* for Luminespib prediction

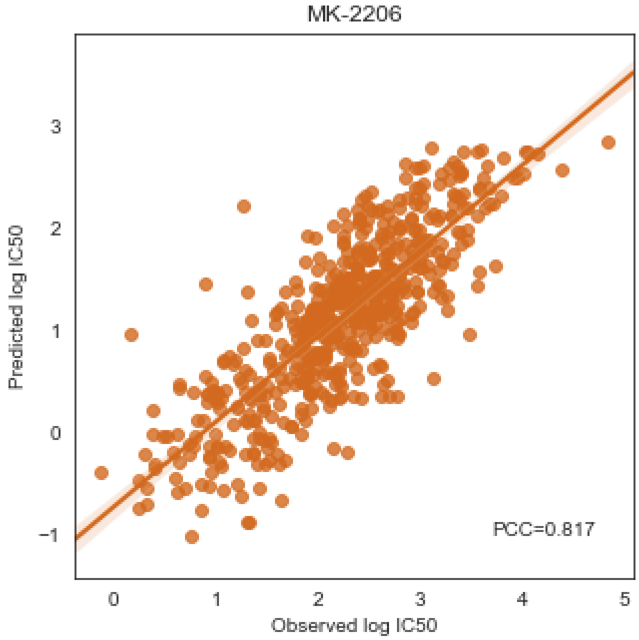

Supp. Fig. S 2: *PCC* for MK-2206 prediction

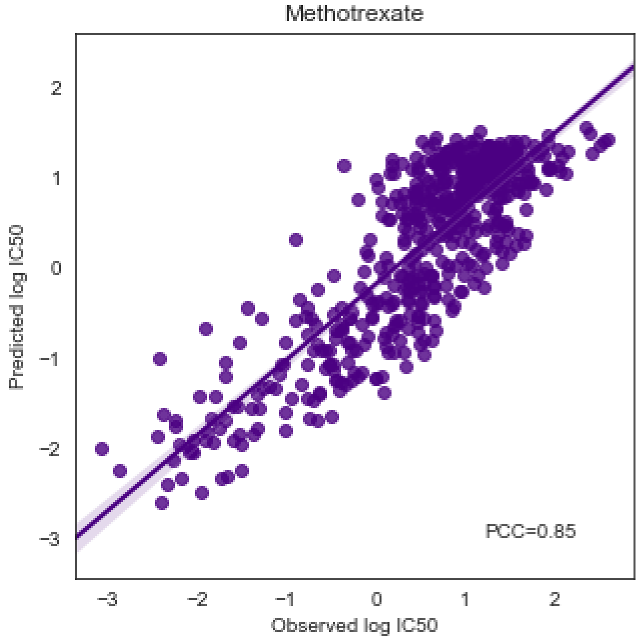

Supp. Fig. S 3: *PCC* for Methotrexate prediction

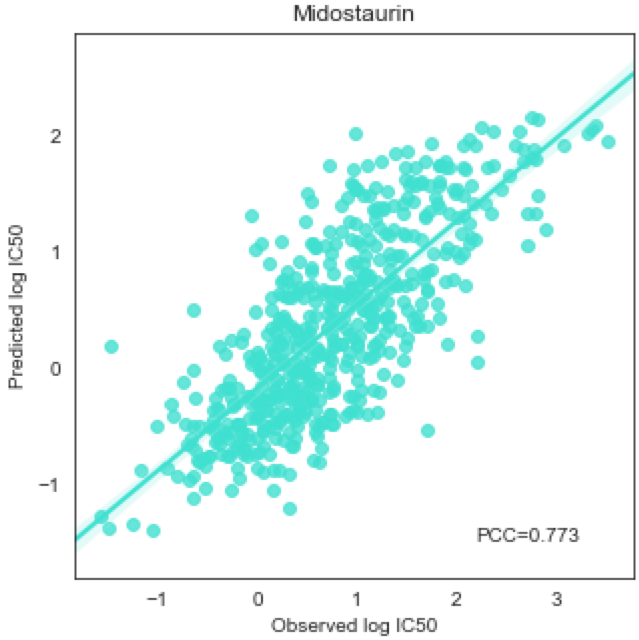

Supp. Fig. S 4: *PCC* for Midostaurin prediction

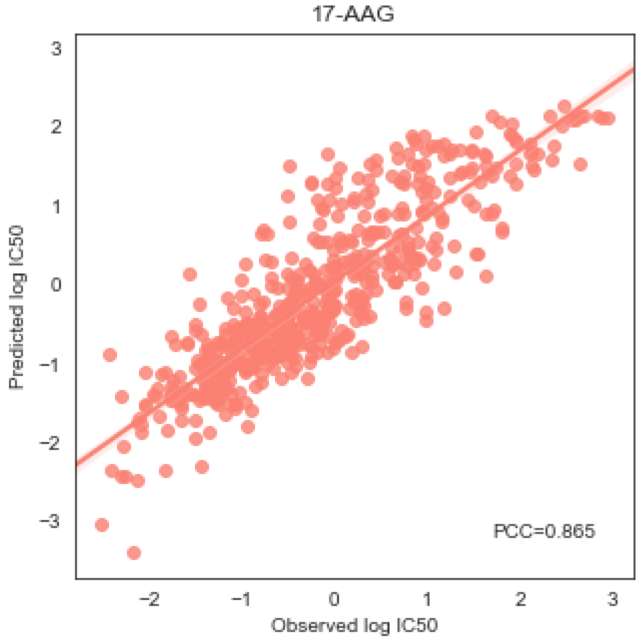

Supp. Fig. S 5: *PCC* for 17-AAG prediction

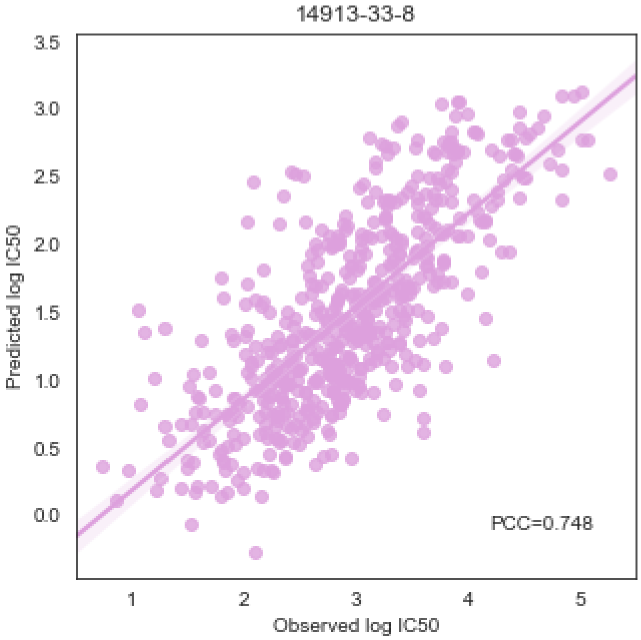

Supp. Fig. S 6: *PCC* for 14913-33-8 prediction

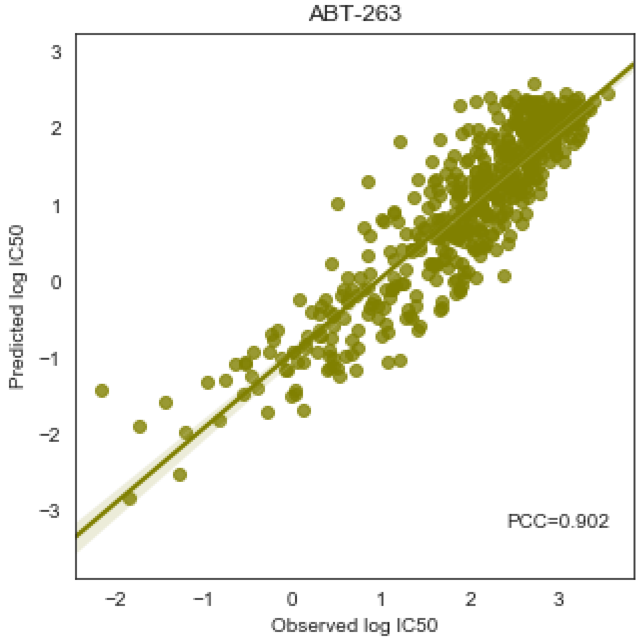

Supp. Fig. S 7: *PCC* for ABT-263 prediction

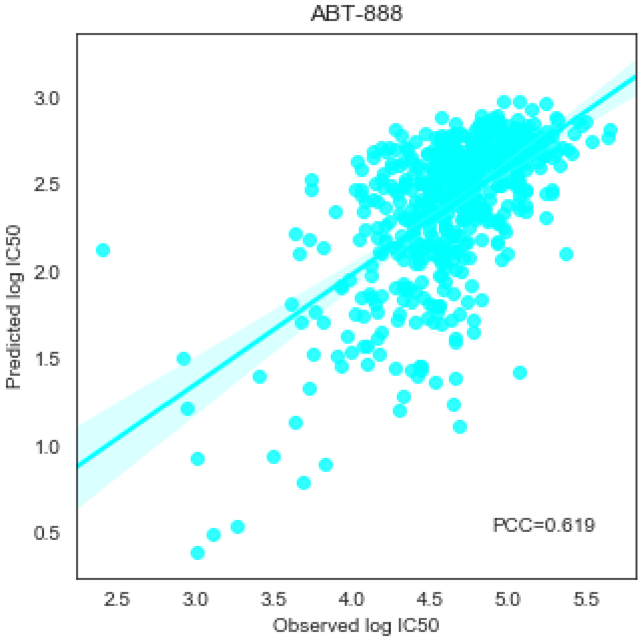

Supp. Fig. S 8: *PCC* for ABT-888 prediction

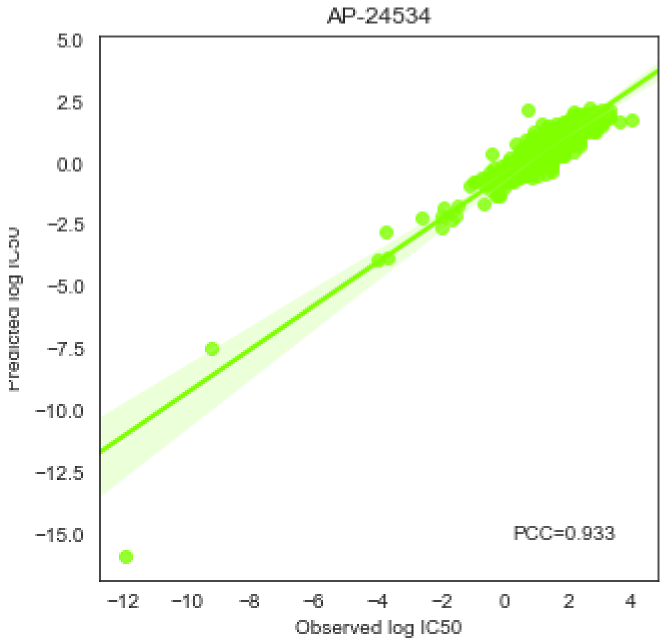

Supp. Fig. S 9: *PCC* for AP-24534 prediction

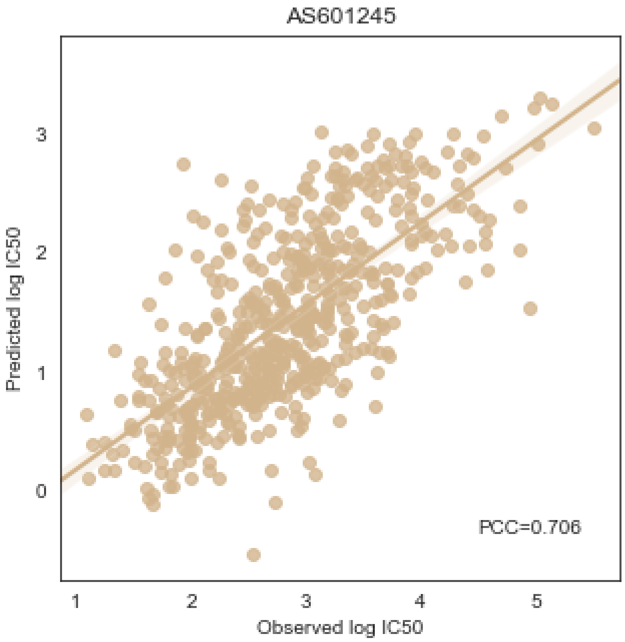

Supp. Fig. S 10: *PCC* for AS601245 prediction

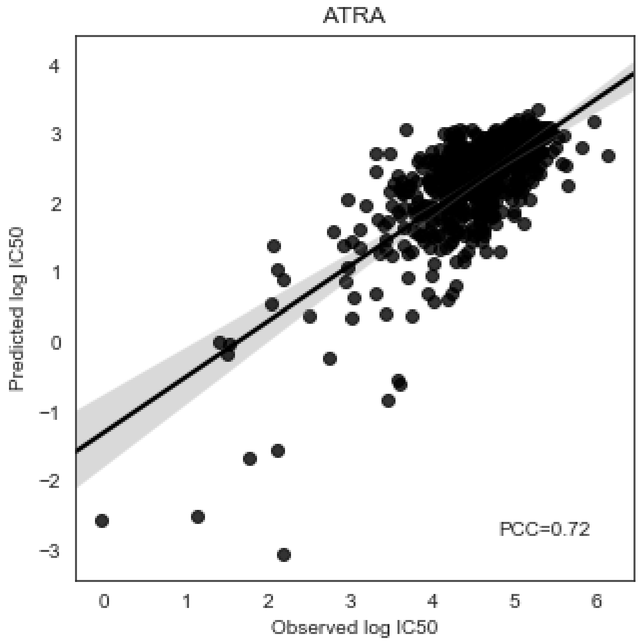

Supp. Fig. S 11: *PCC* for ATRA prediction

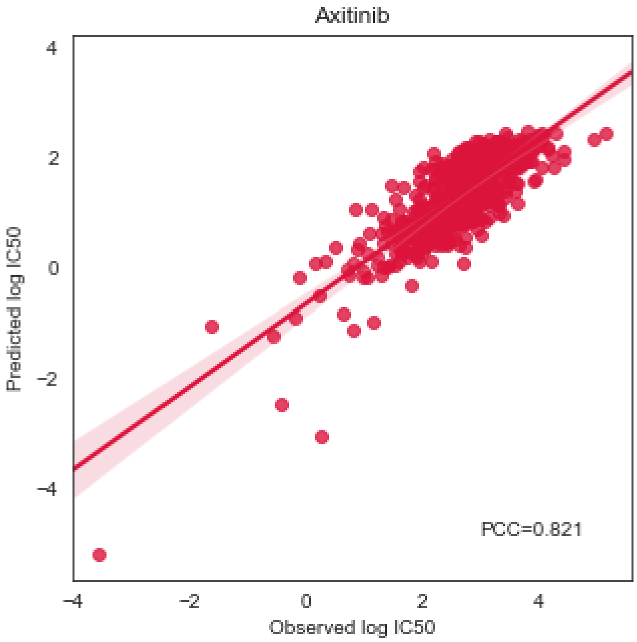

Supp. Fig. S 12: *PCC* for Axitinib prediction

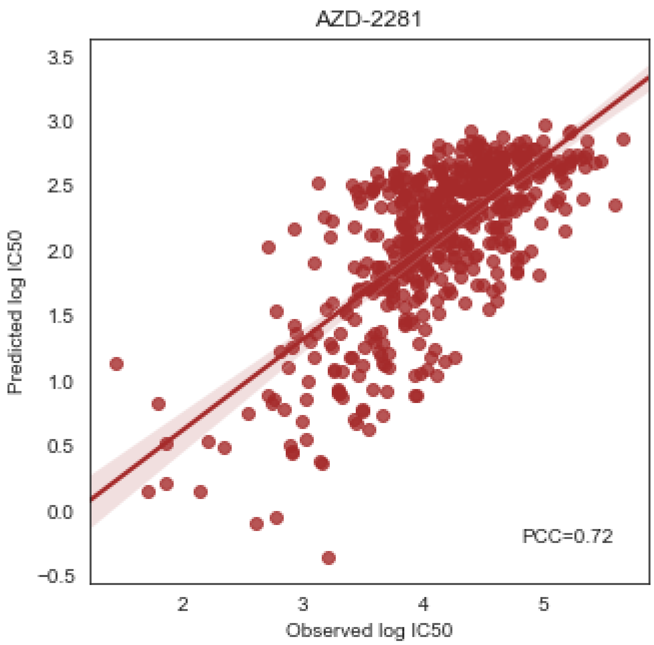

Supp. Fig. S 13: *PCC* for AZD-2281 prediction

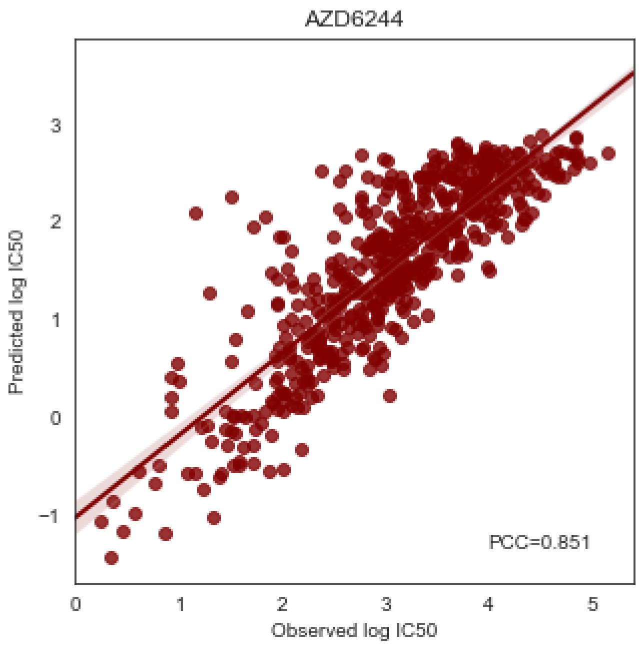

Supp. Fig. S 14: *PCC* for AZD6244 prediction

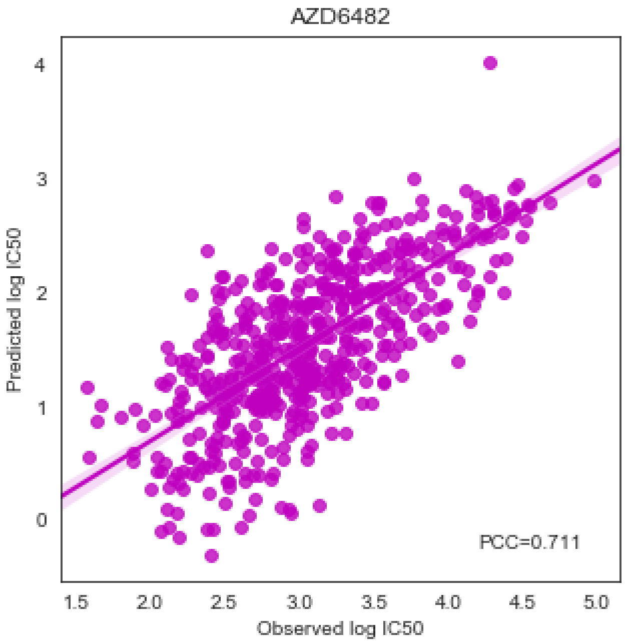

Supp. Fig. S 15: *PCC* for AZD6482 prediction

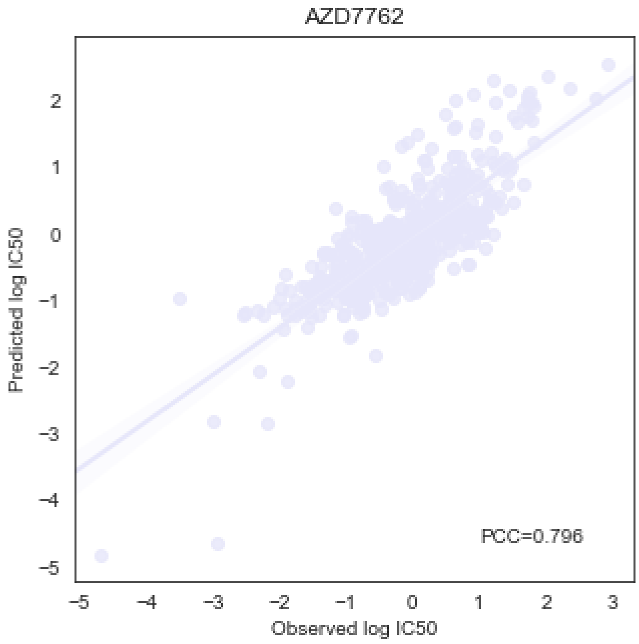

Supp. Fig. S 16: *PCC* for AZD7762 prediction

61-3606.png 61-3606.bb

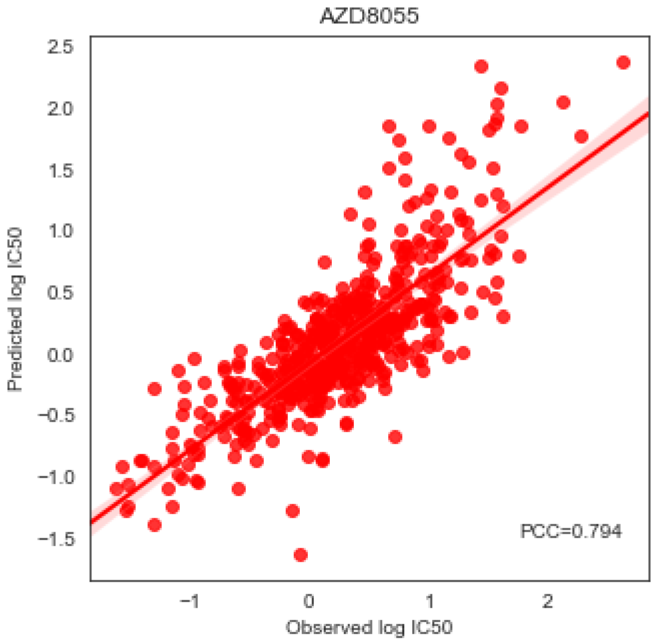

Supp. Fig. S 17: *PCC* for AZD8055 prediction

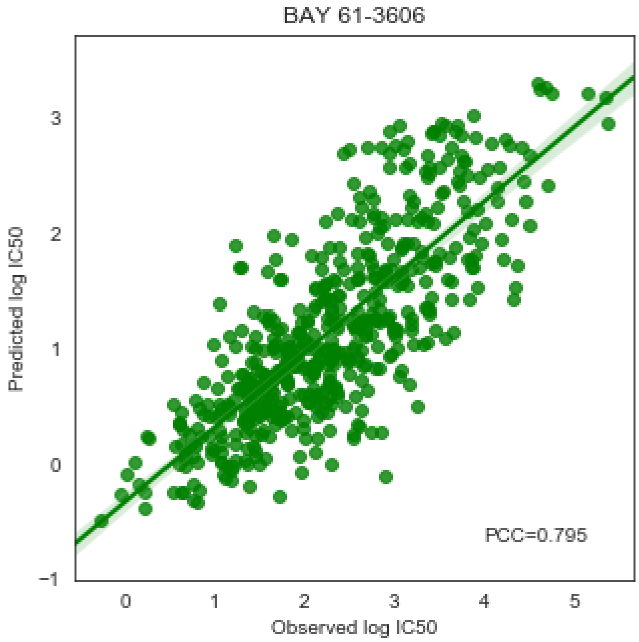

Supp. Fig. S 18: *PCC* for BAY 61-3606 prediction

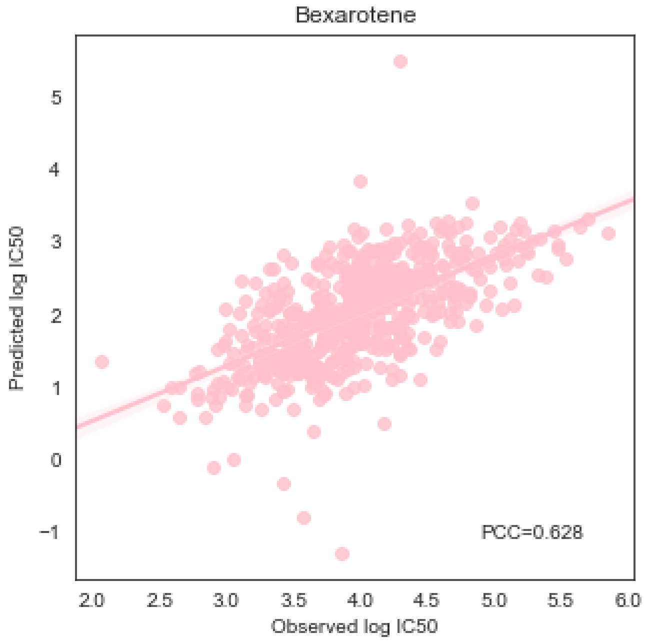

Supp. Fig. S 19: *PCC* for Bexarotene prediction

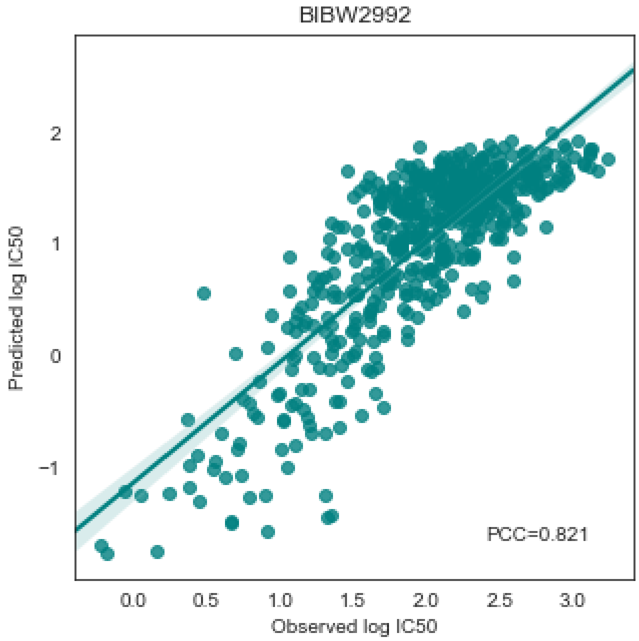

Supp. Fig. S 20: *PCC* for BIBW2992 prediction

0796.png 0796.bb

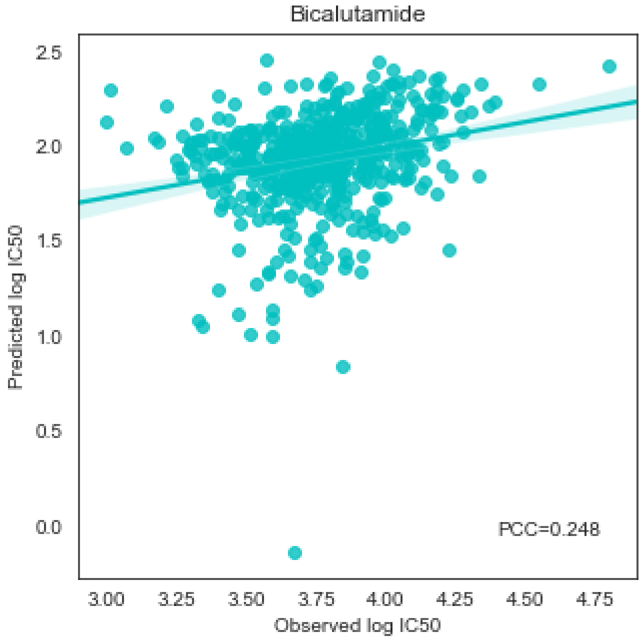

Supp. Fig. S 21: *PCC* for Bicalutamide prediction

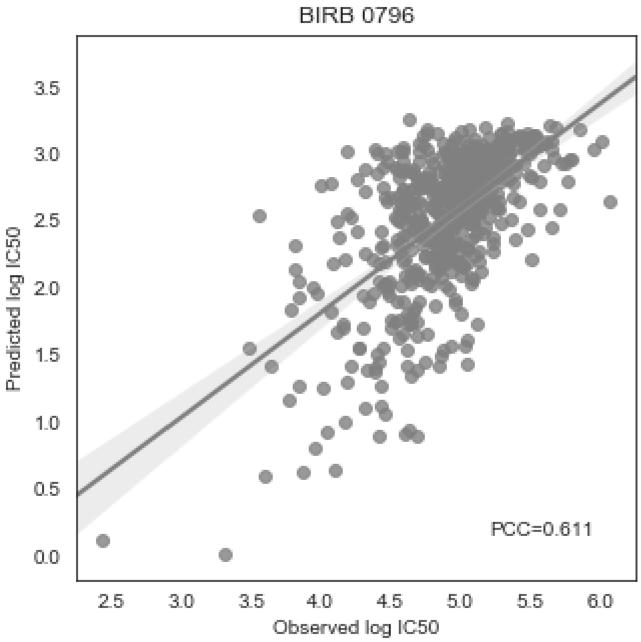

Supp. Fig. S 22: *PCC* for BIRB 0796 prediction

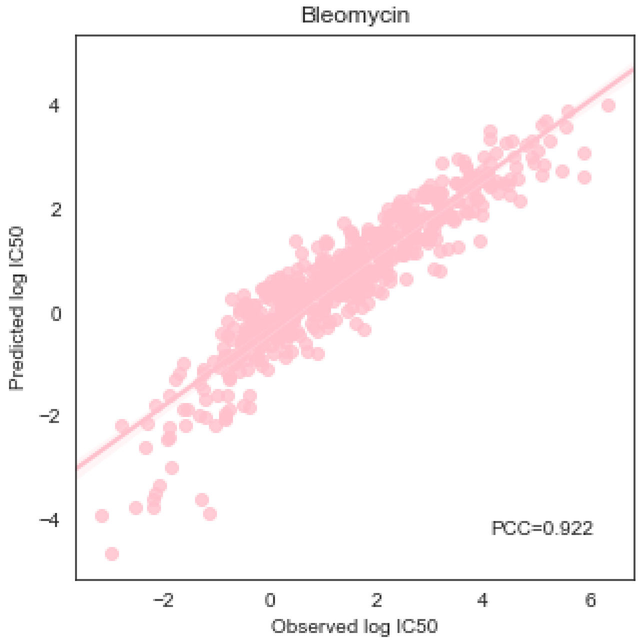

Supp. Fig. S 23: *PCC* for Bleomycin prediction

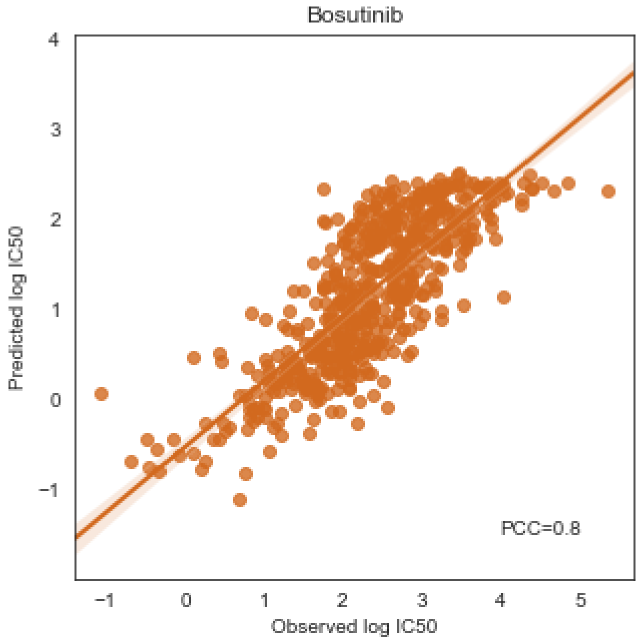

Supp. Fig. S 24: *PCC* for Bosutinib prediction

1.png 1.bb

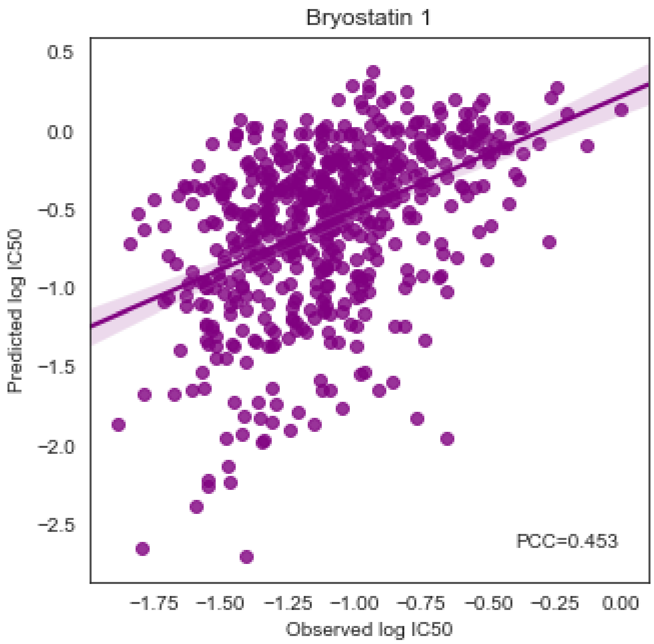

Supp. Fig. S 25: *PCC* for Bryostatin 1 prediction

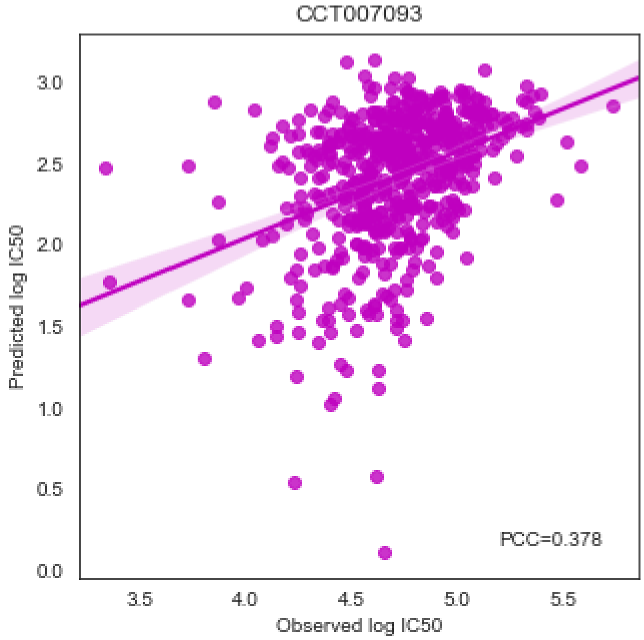

Supp. Fig. S 26: *PCC* for CCT007093 prediction

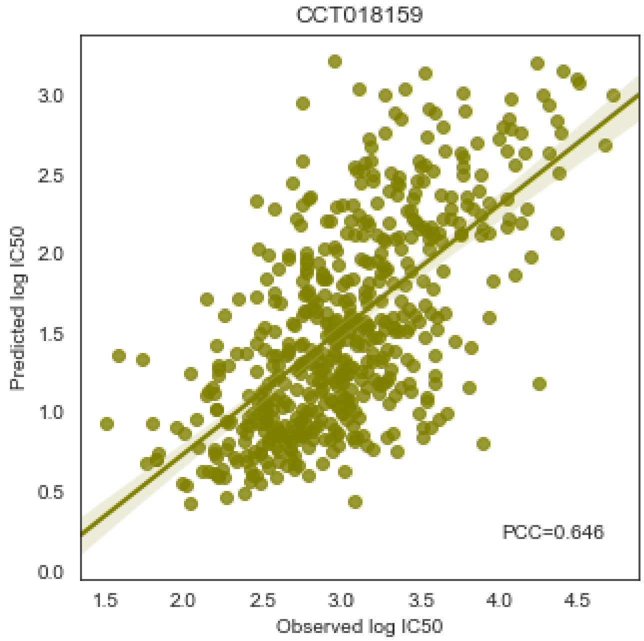

Supp. Fig. S 27: *PCC* for CCT018159 prediction

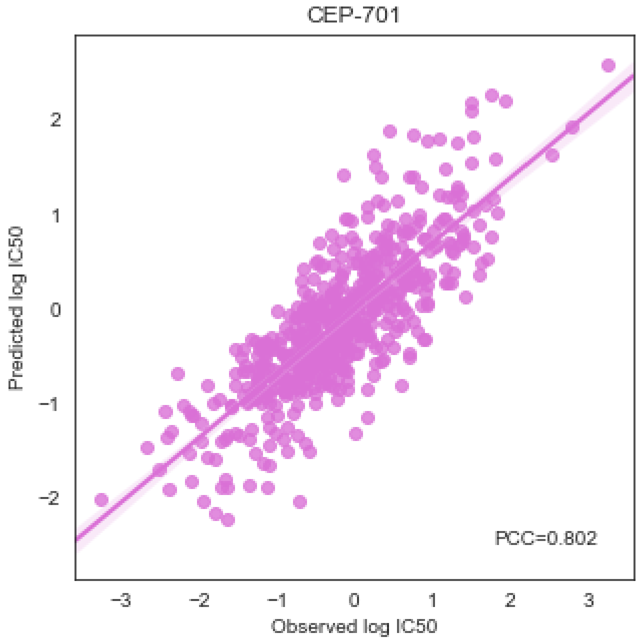

Supp. Fig. S 28: *PCC* for CEP-701 prediction

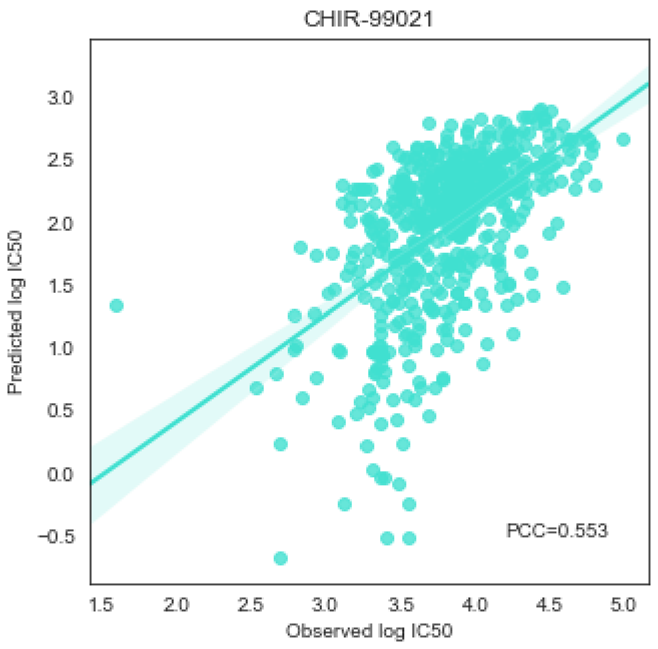

Supp. Fig. S 29: *PCC* for CHIR-99021 prediction

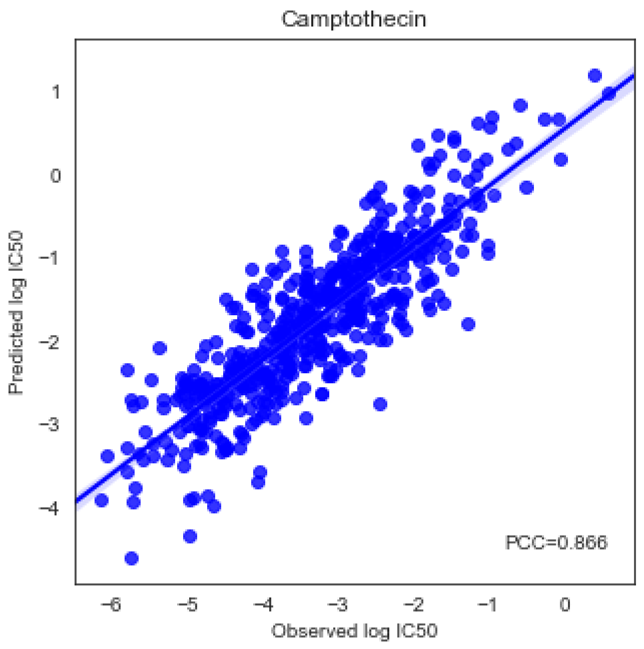

Supp. Fig. S 30: *PCC* for Camptothecin prediction

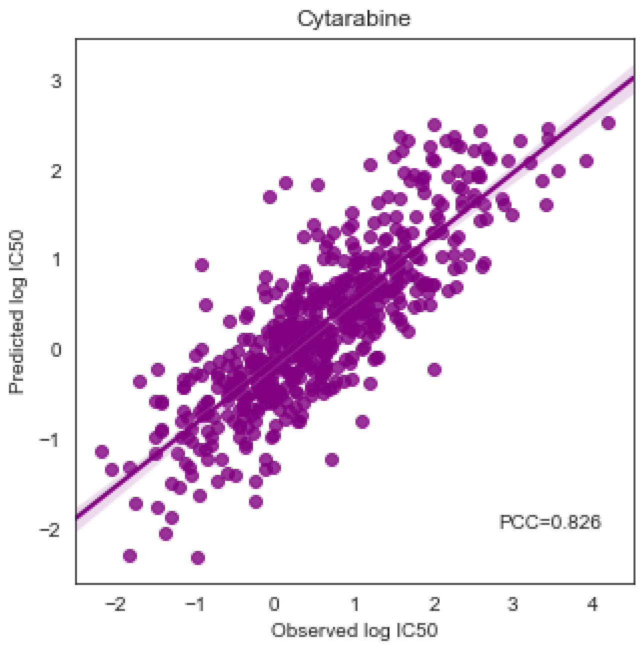

Supp. Fig. S 31: *PCC* for Cytarabine prediction

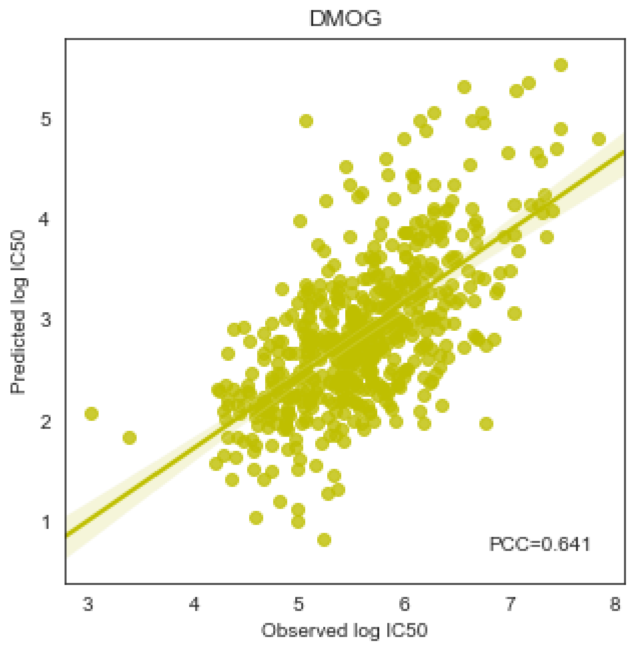

Supp. Fig. S 32: *PCC* for DMOG prediction

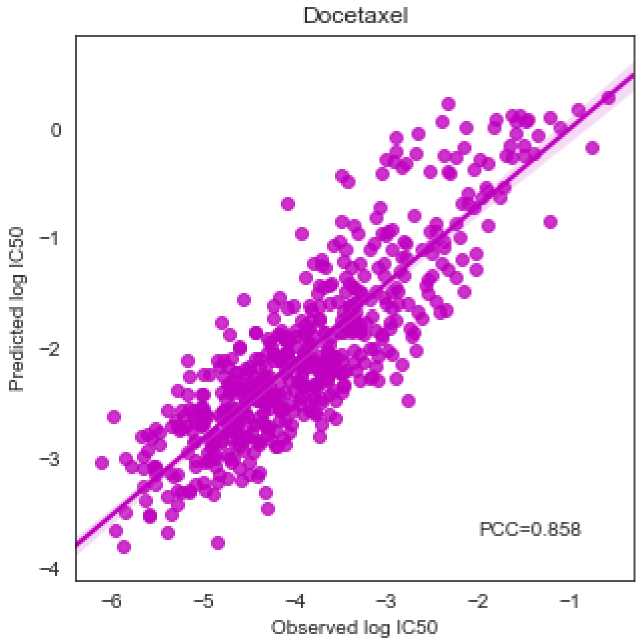

Supp. Fig. S 33: *PCC* for Docetaxel prediction
1864.png 1864.bb

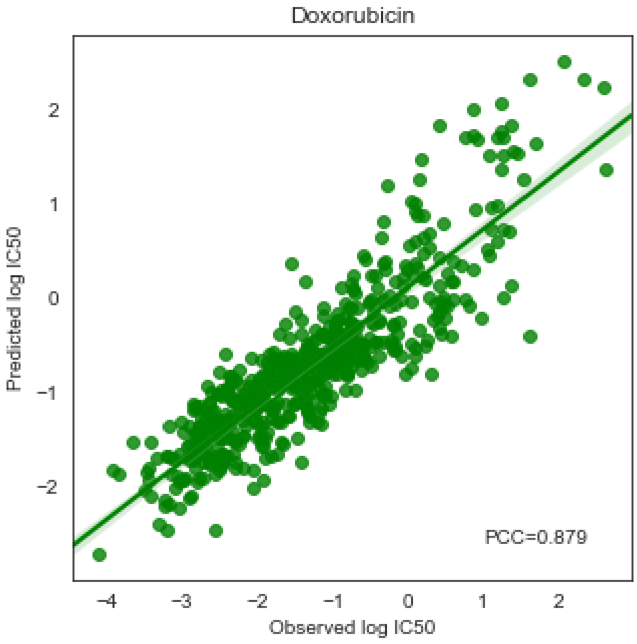

Supp. Fig. S 34: *PCC* for Doxorubicin prediction
B.png B.bb

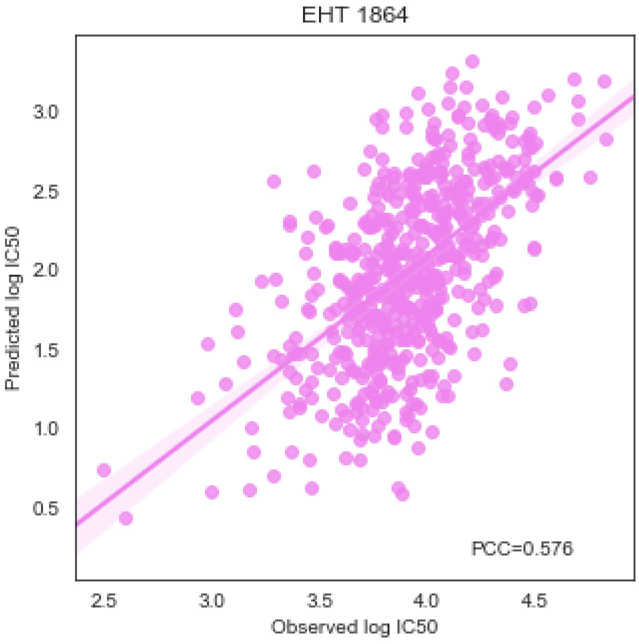

Supp. Fig. S 35: *PCC* for EHT 1864 prediction

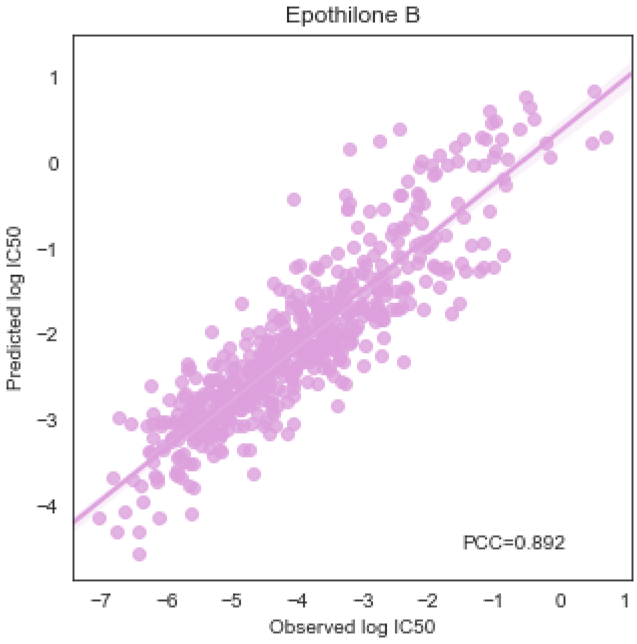

Supp. Fig. S 36: *PCC* for Epothilone B prediction

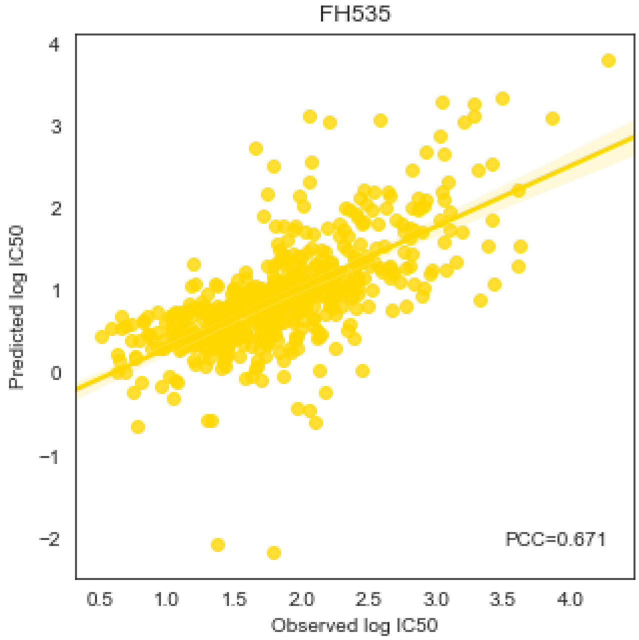

Supp. Fig. S 37: *PCC* for FH535 prediction

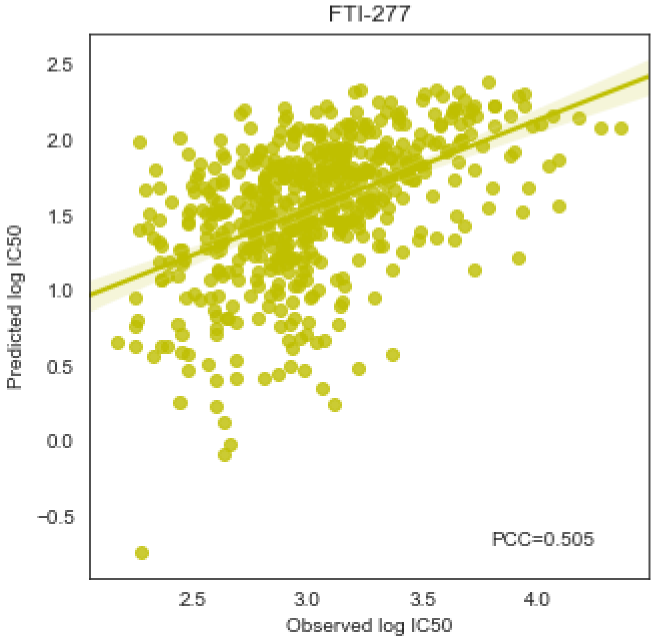

Supp. Fig. S 38: *PCC* for FTI-277 prediction

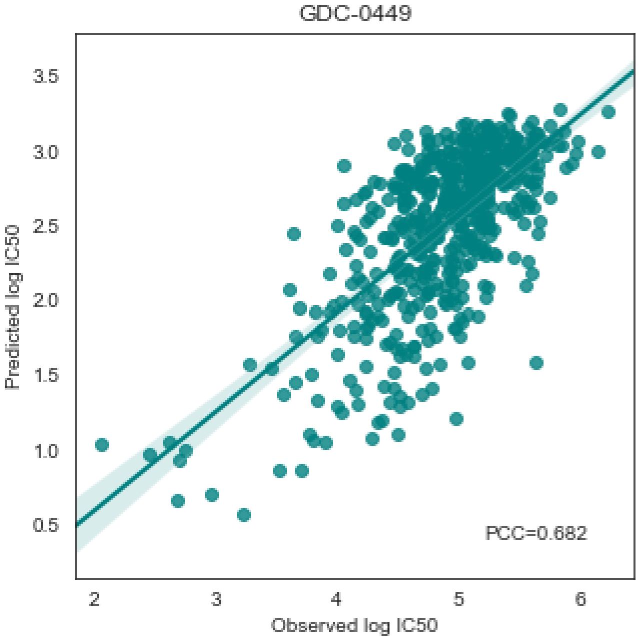

Supp. Fig. S 39: *PCC* for GDC-0449 prediction

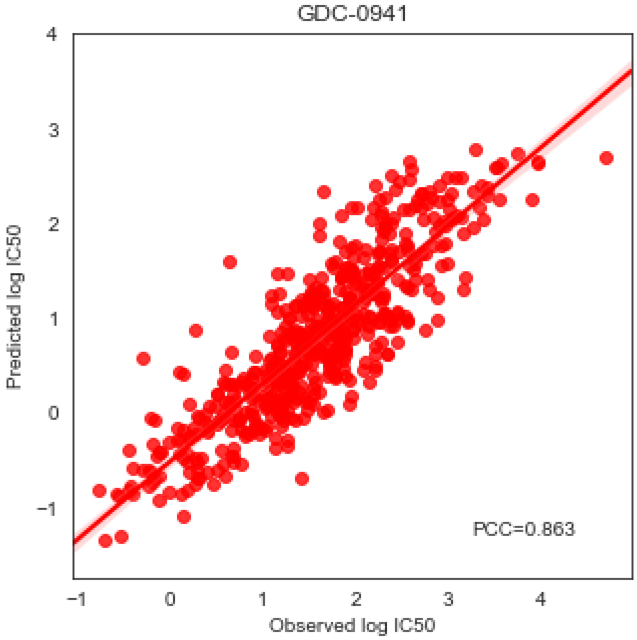

Supp. Fig. S 40: *PCC* for GDC-0449 prediction

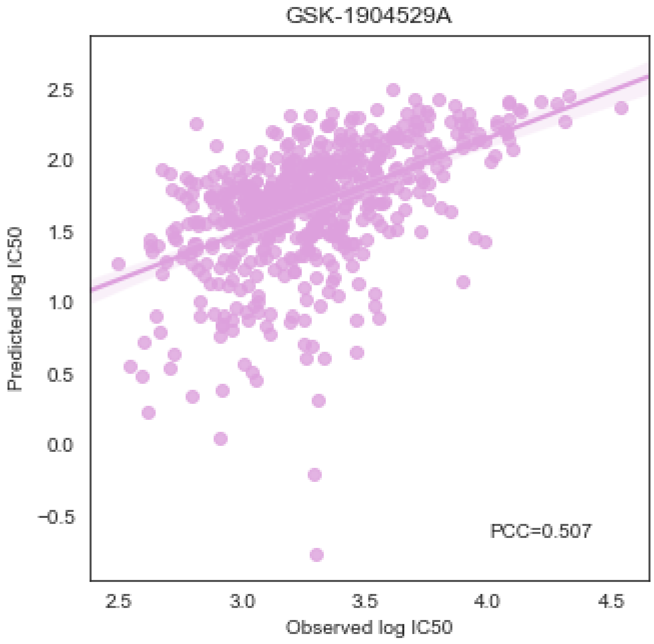

Supp. Fig. S 41: *PCC* for GSK-1904529A prediction
441756.png 441756.bb

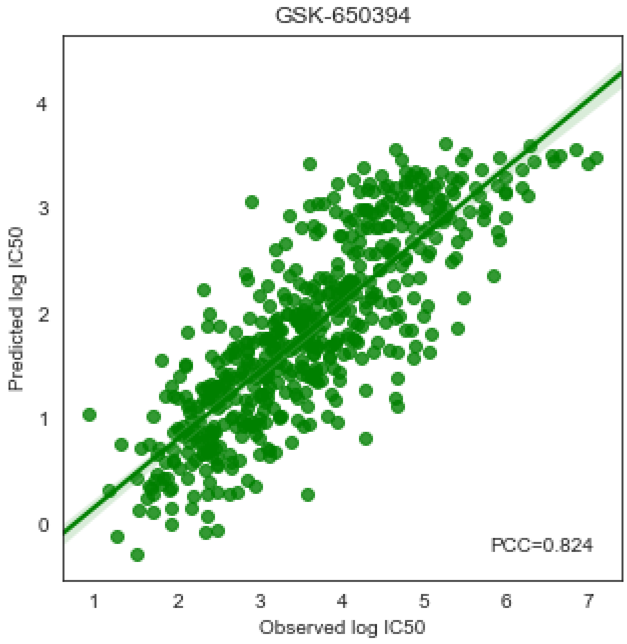

Supp. Fig. S 42: *PCC* for GSK-650394 prediction

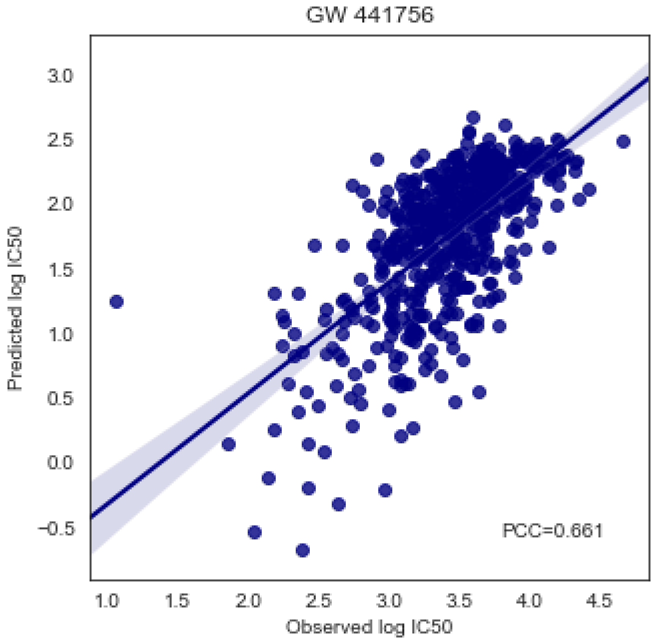

Supp. Fig. S 43: *PCC* for GW 441756 prediction

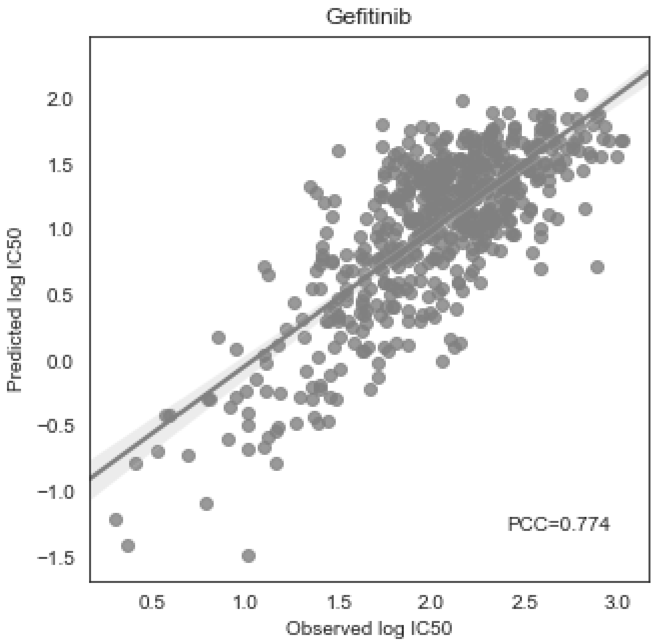

Supp. Fig. S 44: *PCC* for Gefitinib prediction

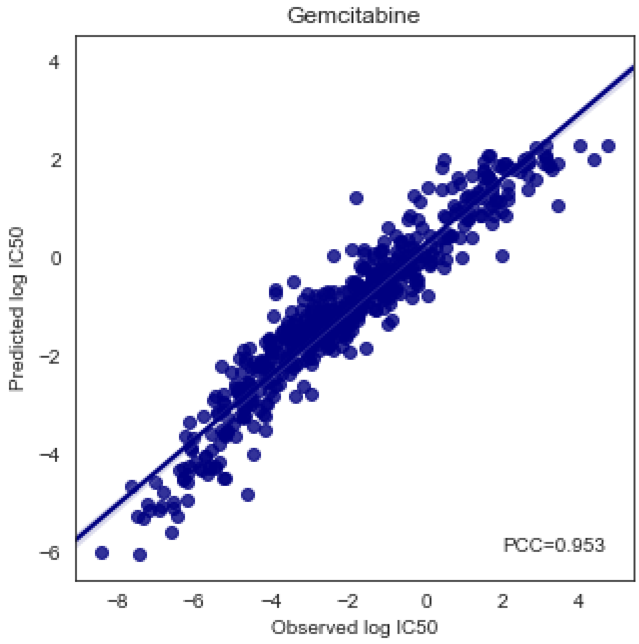

Supp. Fig. S 45: *PCC* for Gemcitabine prediction

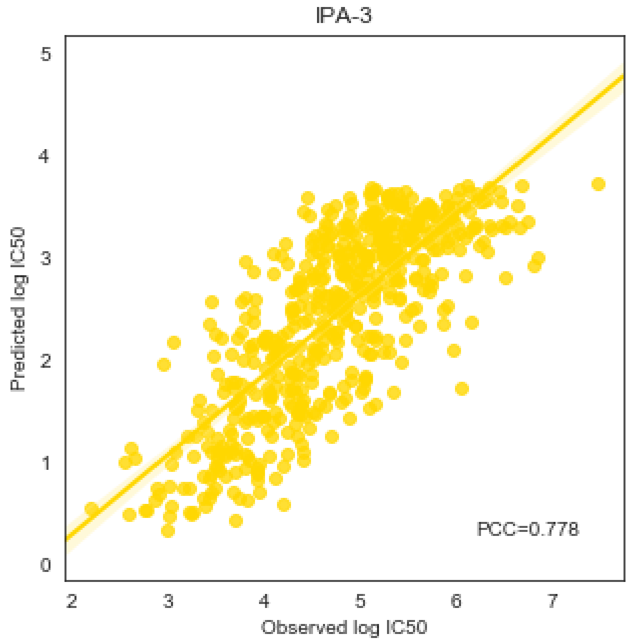

Supp. Fig. S 46: *PCC* for IPA-3 prediction
Inhibitor VIII.png Inhibitor VIII.bb

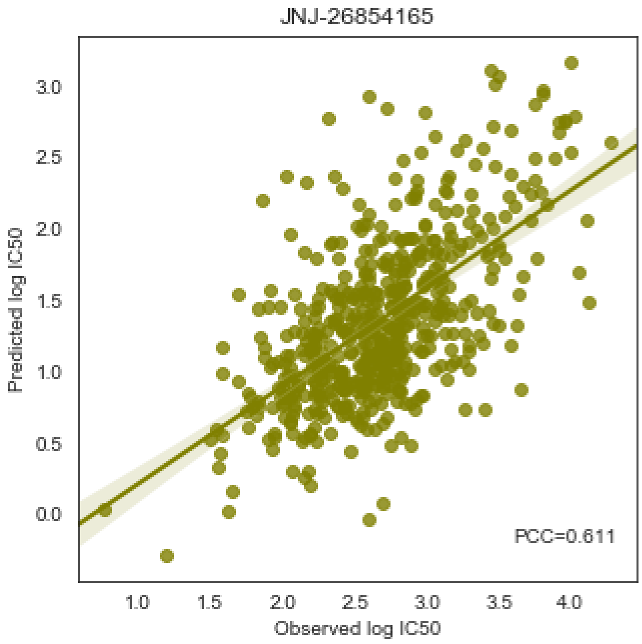

Supp. Fig. S 47: *PCC* for JNJ-26854165 prediction

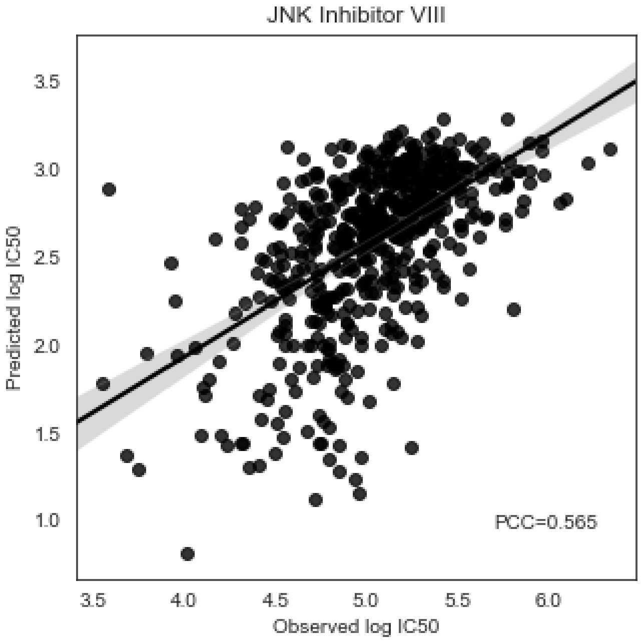

Supp. Fig. S 48: *PCC* for JNK Inhibitor VIII prediction

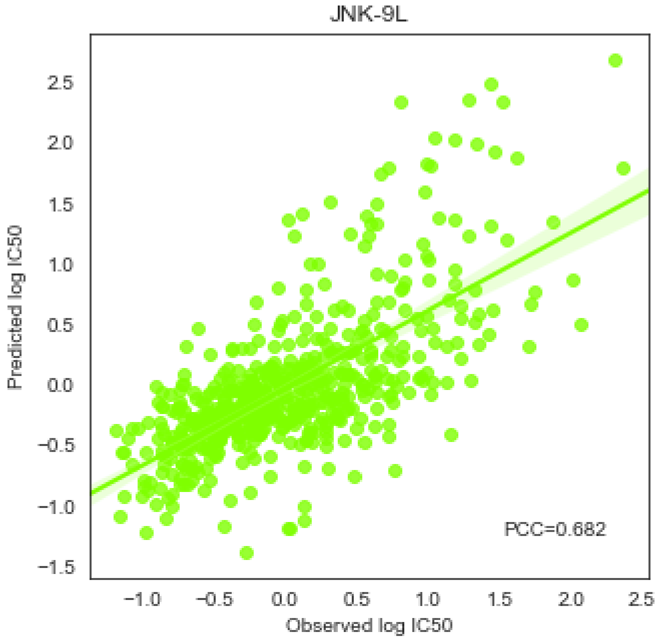

Supp. Fig. S 49: *PCC* for JNK-9L prediction

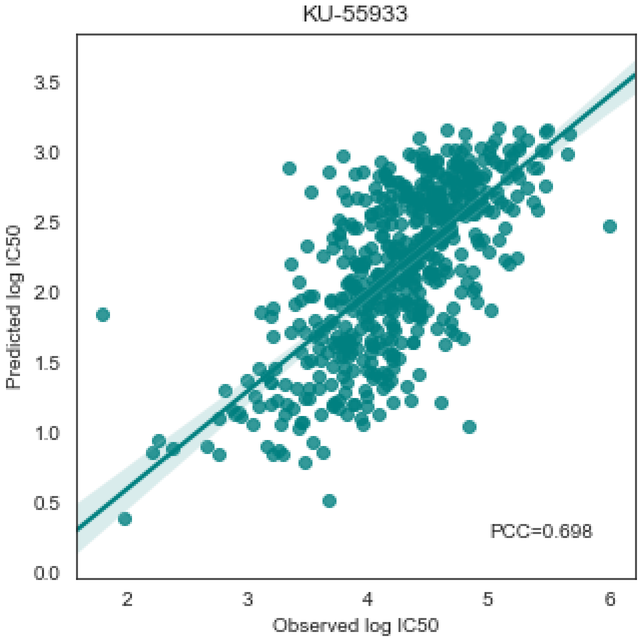

Supp. Fig. S 50: *PCC* for KU-55933 prediction

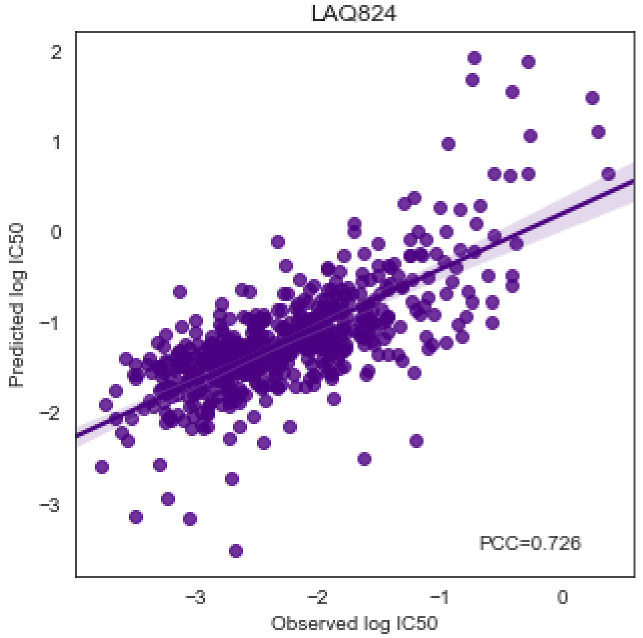

Supp. Fig. S 51: *PCC* for LAQ824 prediction

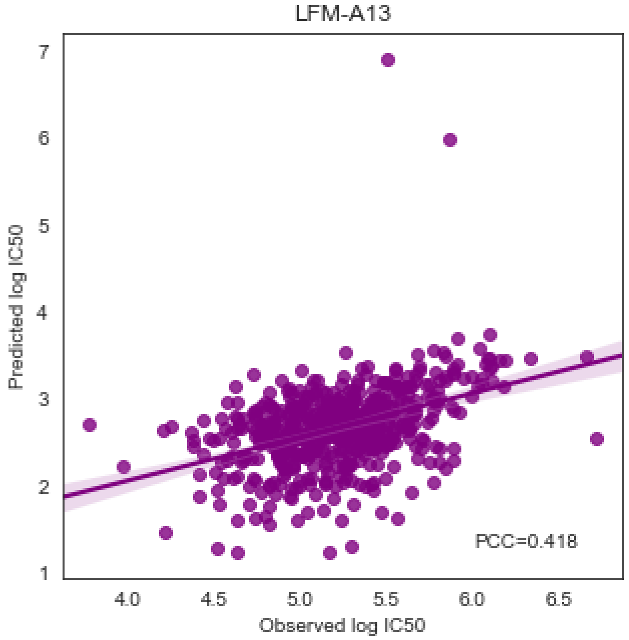

Supp. Fig. S 52: *PCC* for LFM-A13 prediction

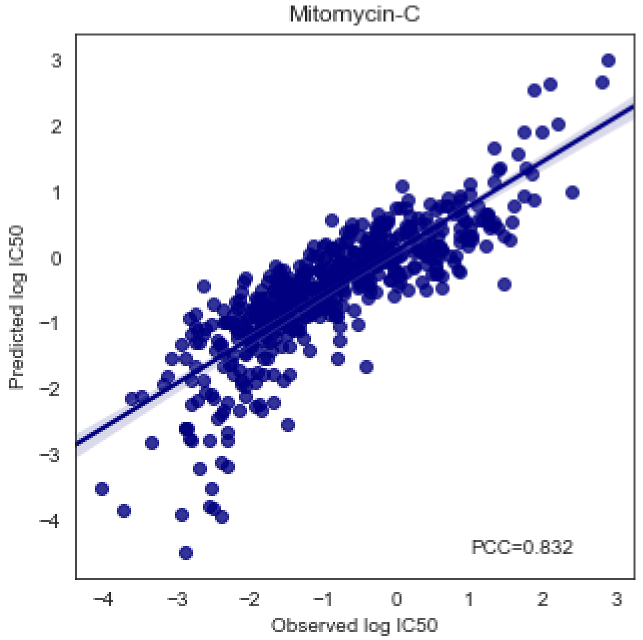

Supp. Fig. S 53: *PCC* for Mitomycin-C prediction

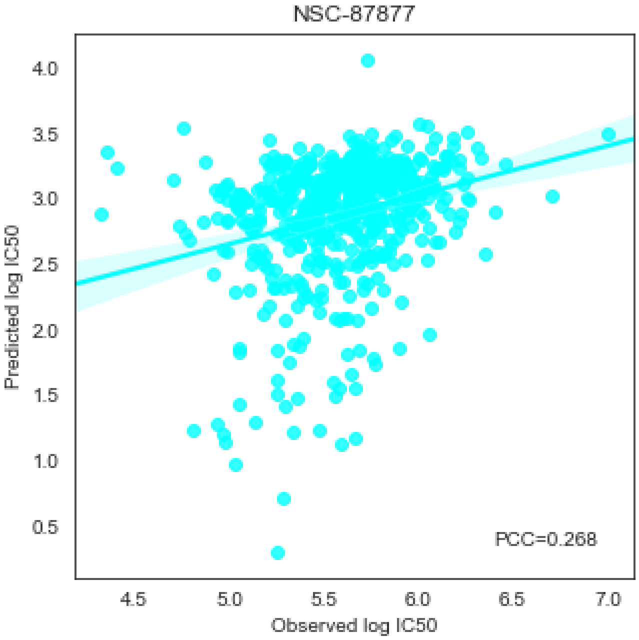

Supp. Fig. S 54: *PCC* for NSC-87877 prediction

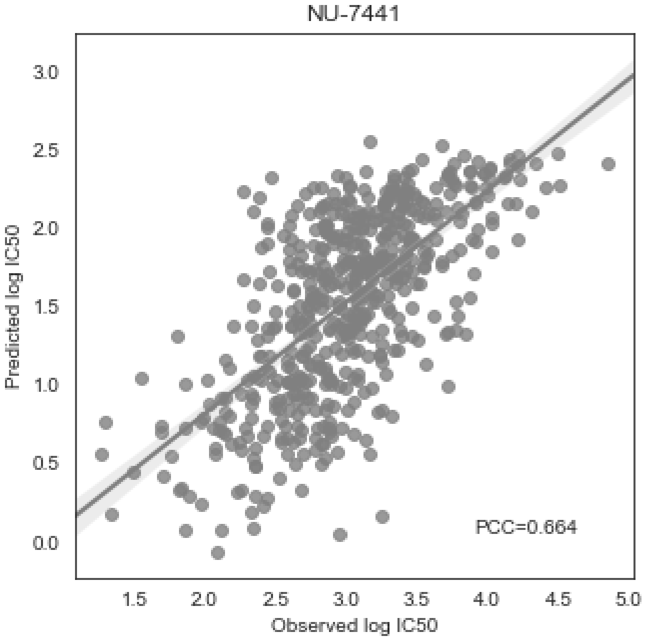

Supp. Fig. S 55: *PCC* for NU-7441 prediction

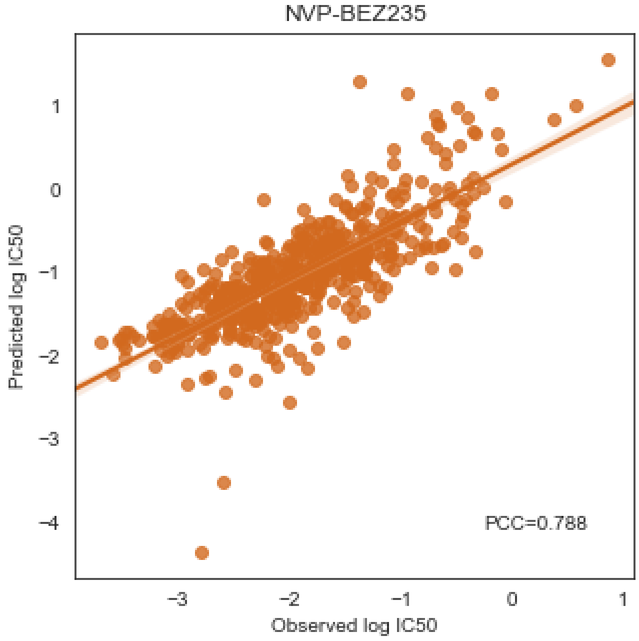

Supp. Fig. S 56: *PCC* for NVP-BEZ235 prediction

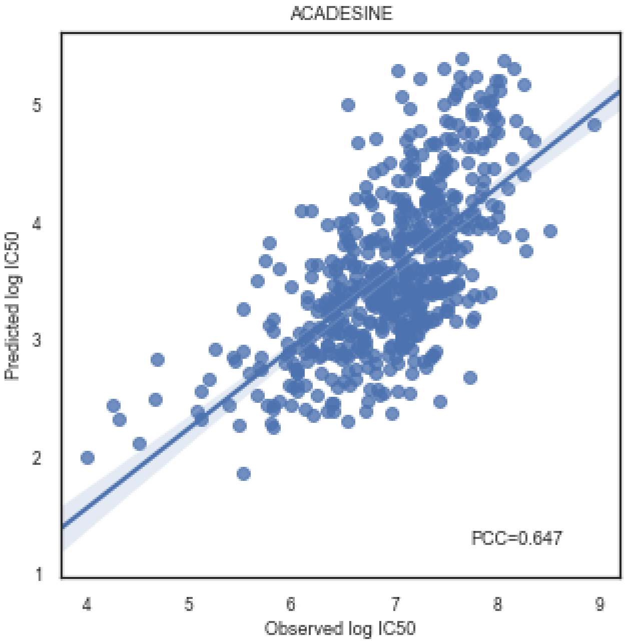

Supp. Fig. S 57: *PCC* for ACADESINE prediction inhibitor VIII.png inhibitor VIII.bb

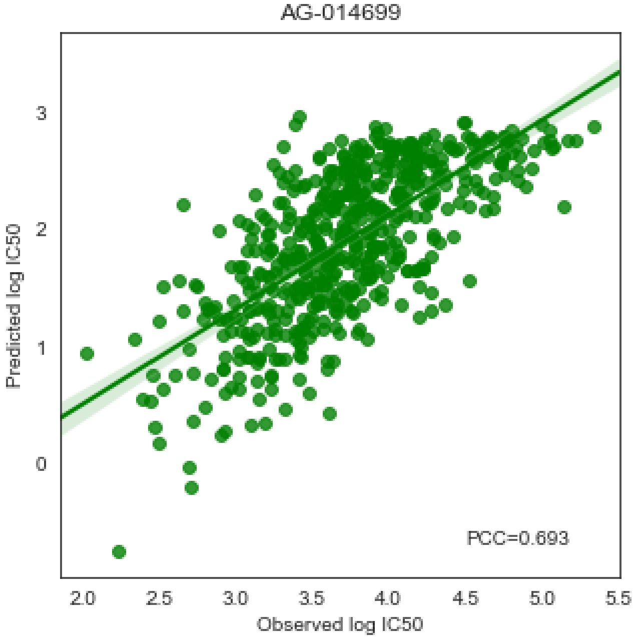

Supp. Fig. S 58: *PCC* for AG-014699 prediction

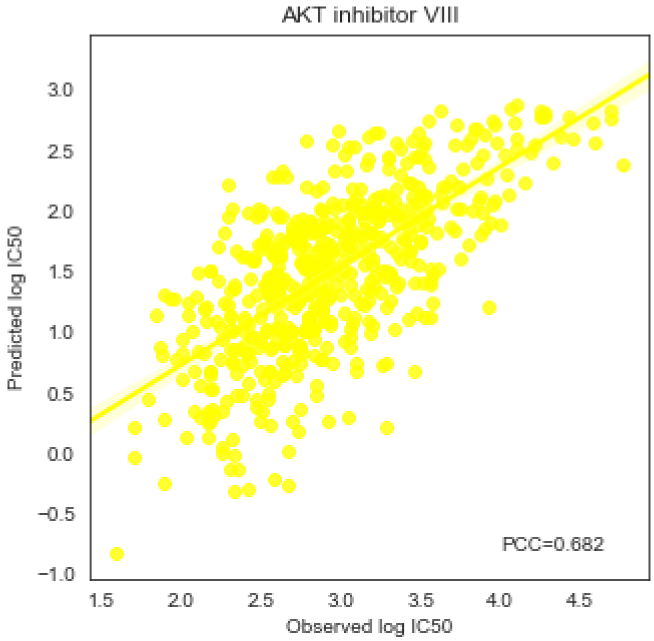

Supp. Fig. S 59: *PCC* for AKT inhibitor VIII prediction

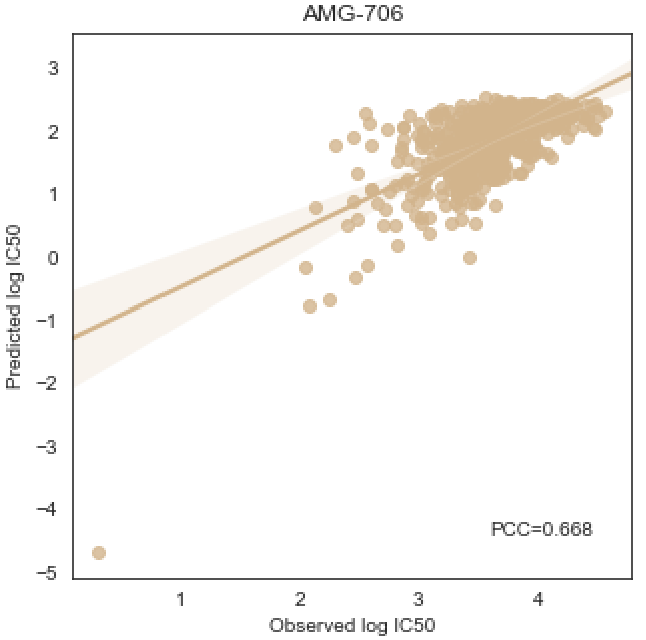

Supp. Fig. S 60: *PCC* for AMG-706 prediction

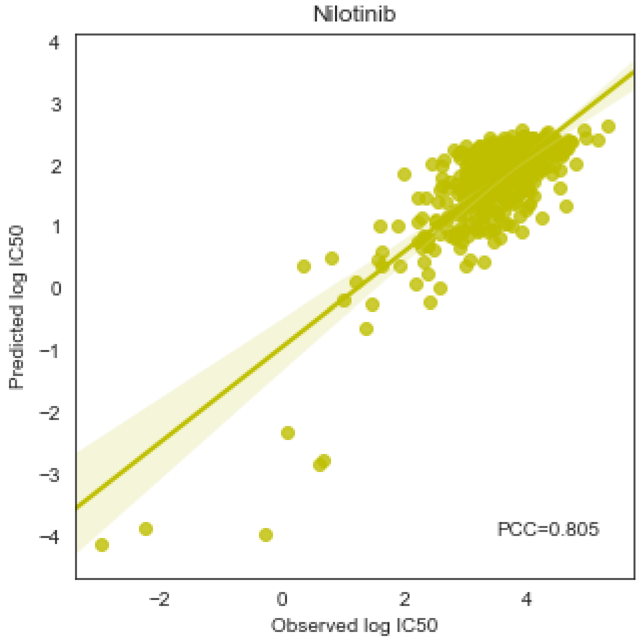

Supp. Fig. S 61: *PCC* for Nilotinib prediction

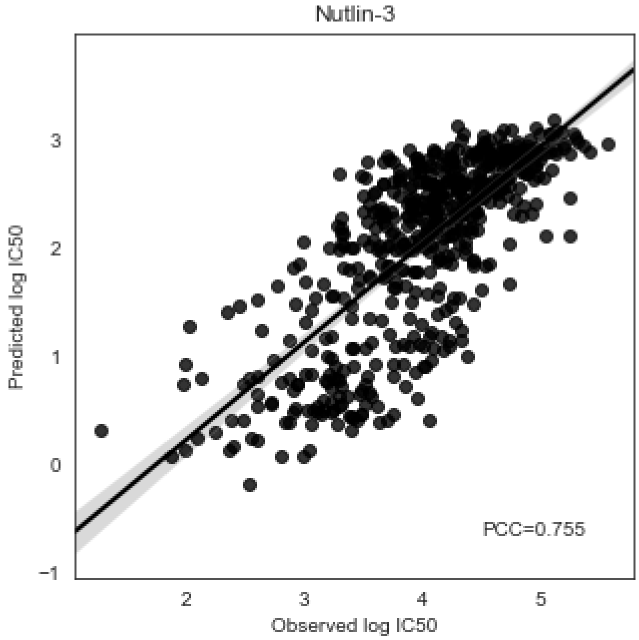

Supp. Fig. S 62: *PCC* for Nutlin-3 prediction

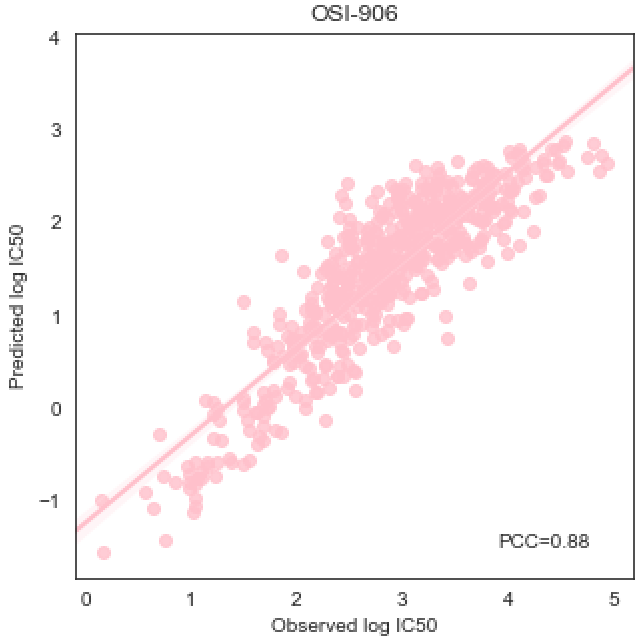

Supp. Fig. S 63: *PCC* for OSI-906 prediction

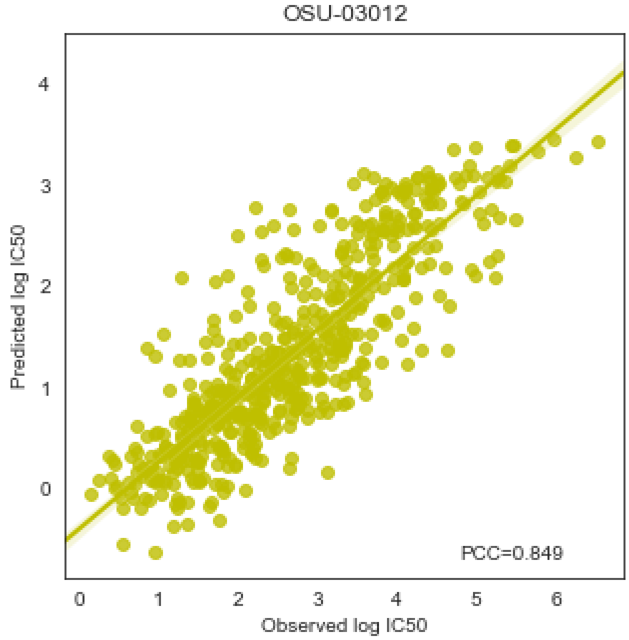

Supp. Fig. S 64: *PCC* for OSU-03012 prediction

Mesylate.png Mesylate.bb

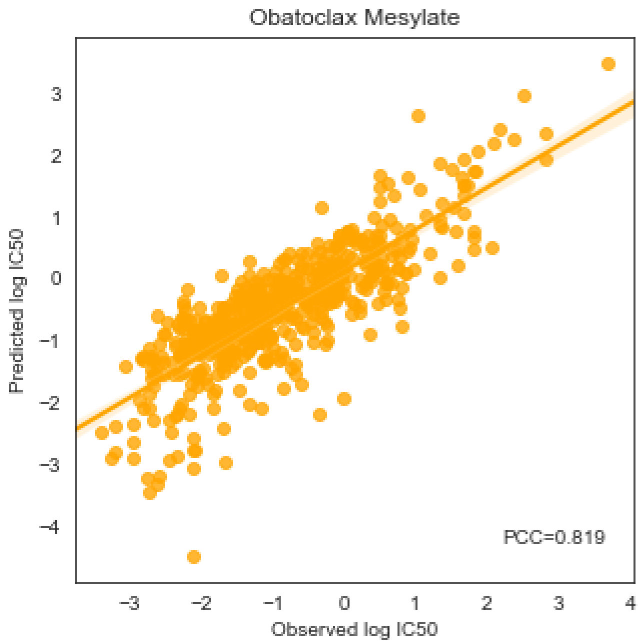

Supp. Fig. S 65: *PCC* for Obatoclax Mesylate prediction

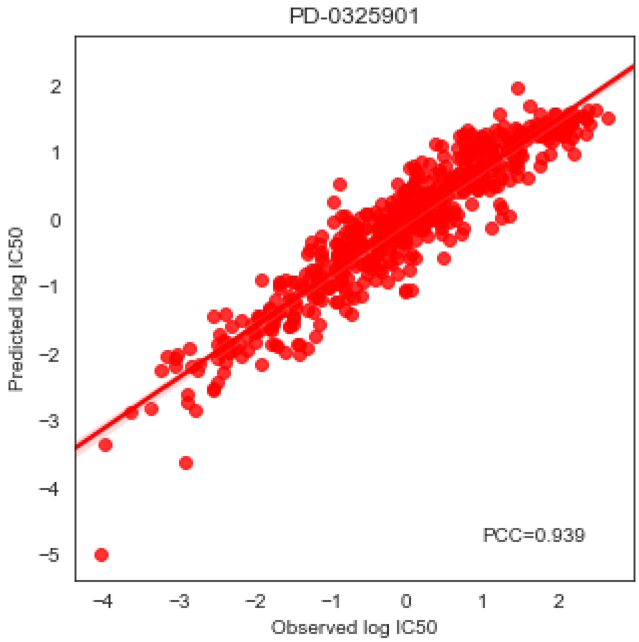

Supp. Fig. S 66: *PCC* for PD-0325901 prediction

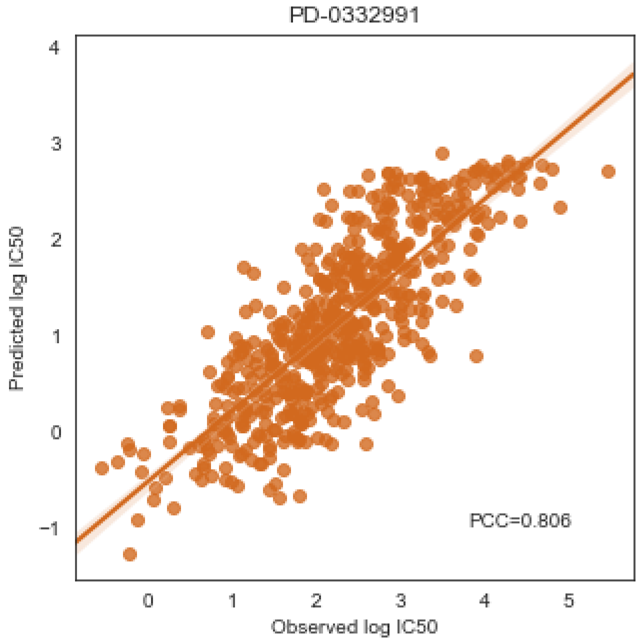

Supp. Fig. S 67: *PCC* for PD-0332991 prediction

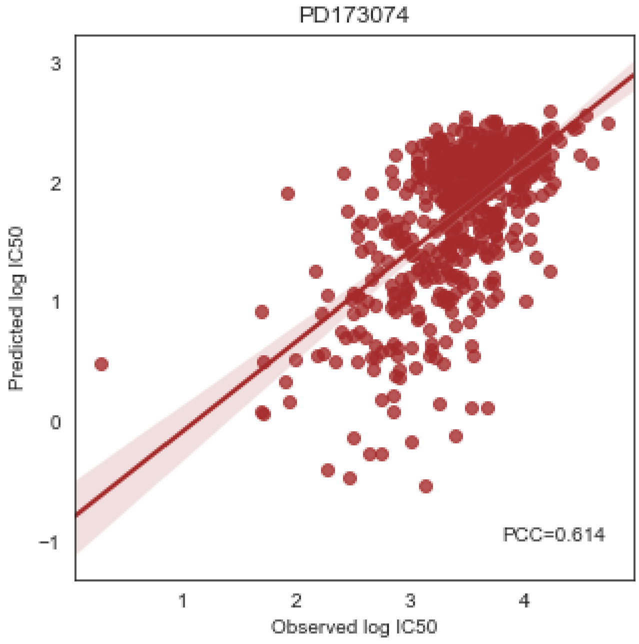

Supp. Fig. S 68: *PCC* for PD173074 prediction

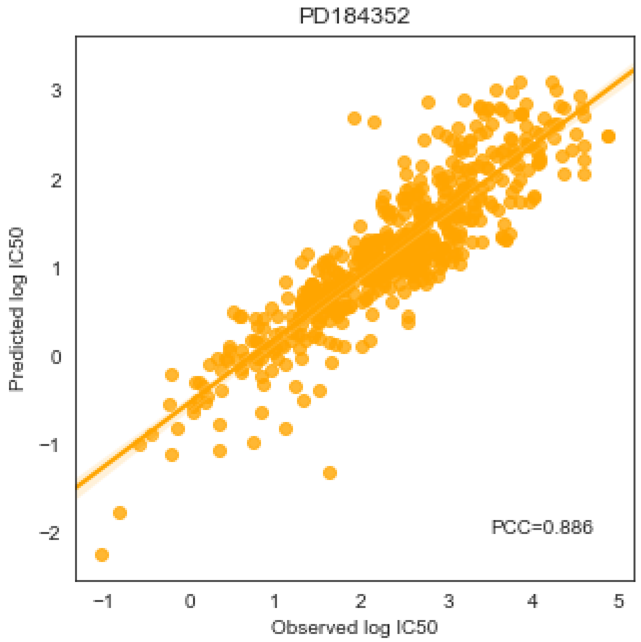

Supp. Fig. S 69: *PCC* for PD184352 prediction

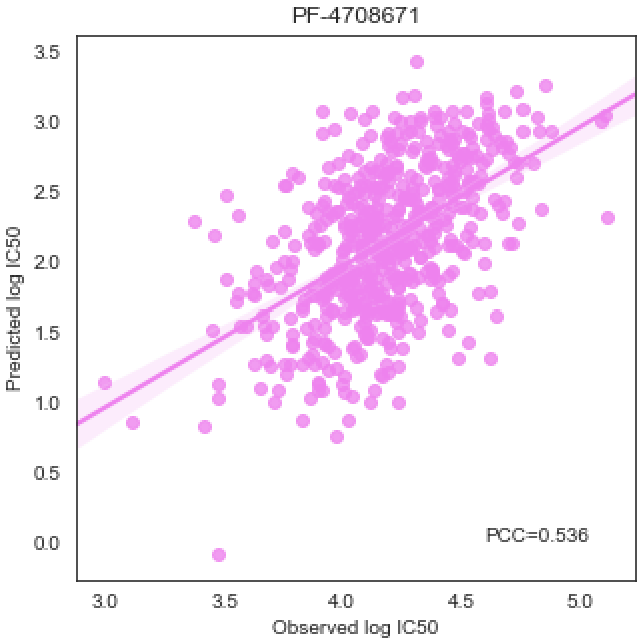

Supp. Fig. S 70: *PCC* for PF-4708671 prediction

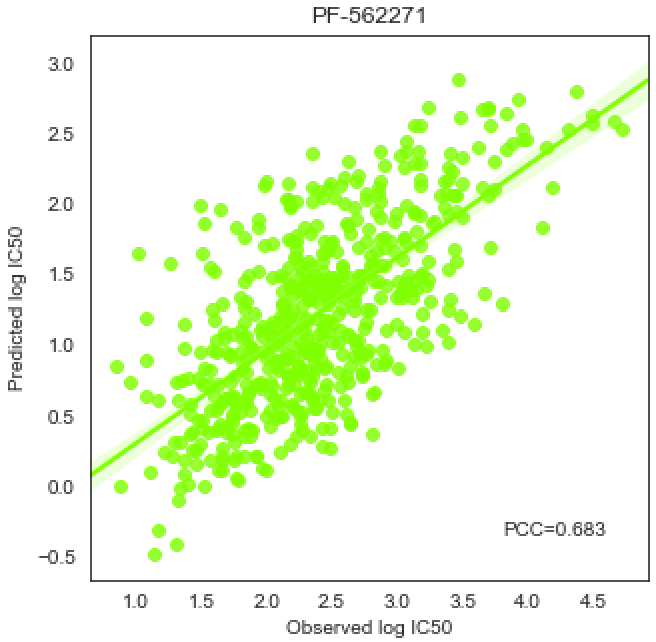

Supp. Fig. S 71: *PCC* for PF-562271 prediction

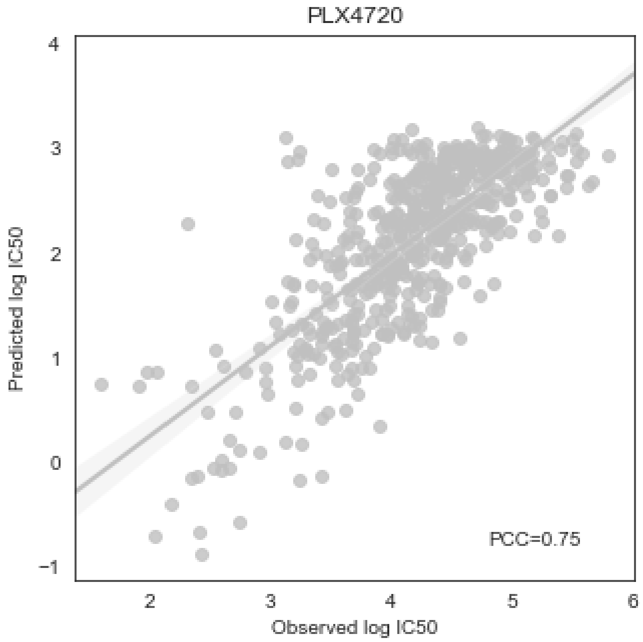

Supp. Fig. S 72: *PCC* for PLX4720 prediction

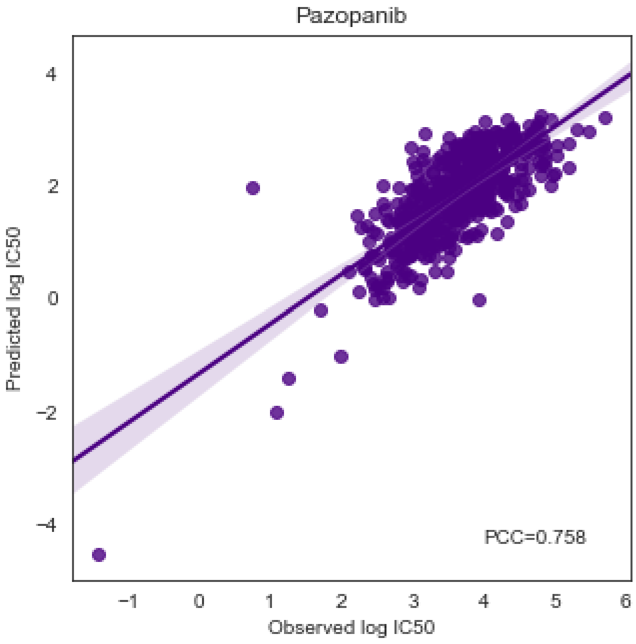

Supp. Fig. S 73: *PCC* for Pazopanib prediction

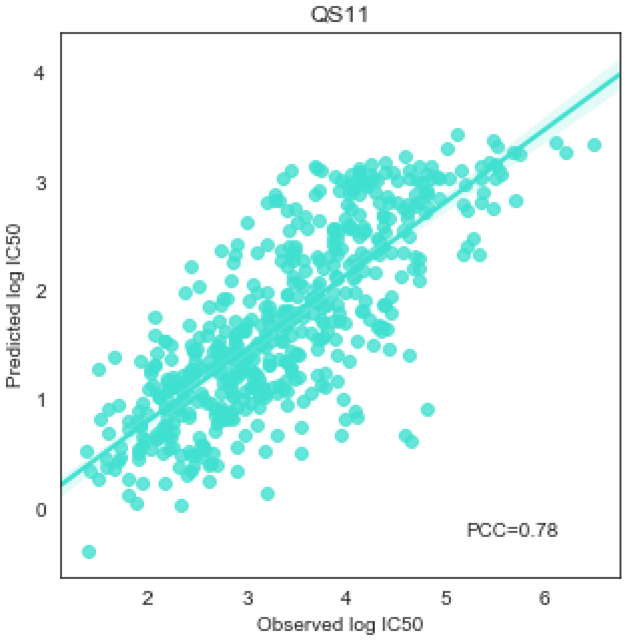

Supp. Fig. S 74: *PCC* for QS11 prediction
216763.png 216763.bb

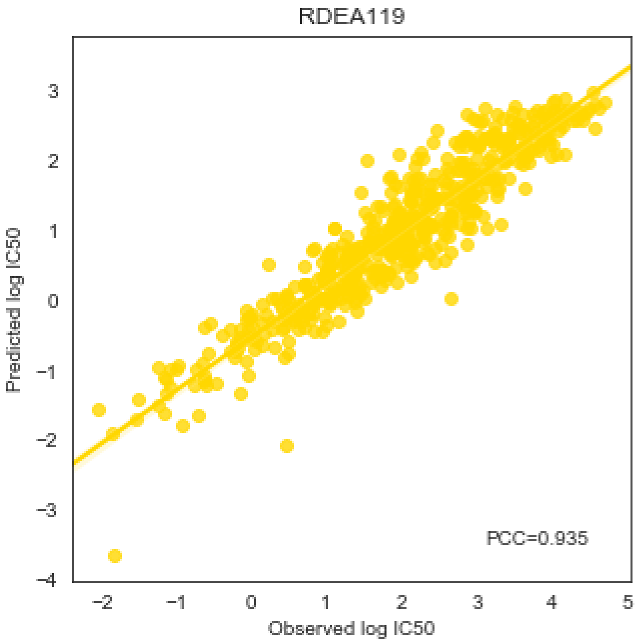

Supp. Fig. S 75: *PCC* for RDEA119 prediction

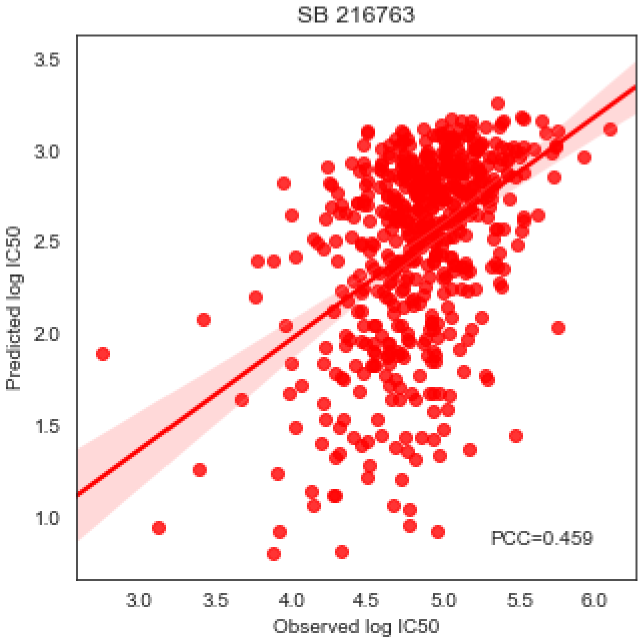

Supp. Fig. S 76: *PCC* for SB 216763 prediction

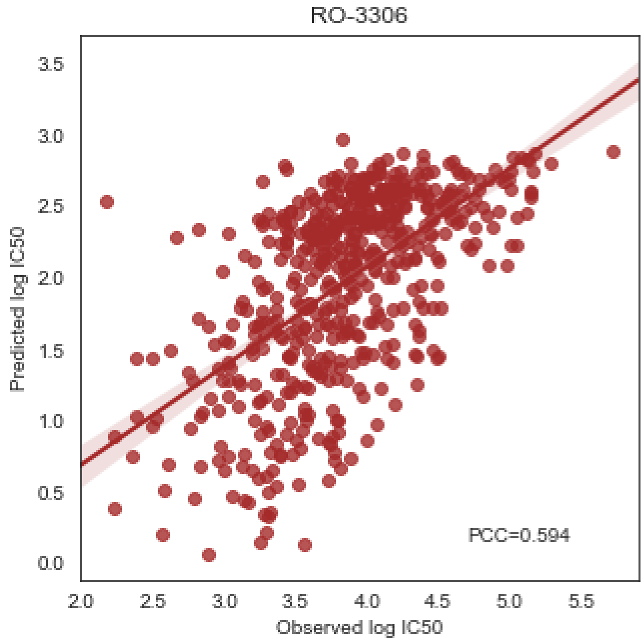

Supp. Fig. S 77: *PCC* for RO-3306 prediction
0101-1.png 0101-1.bb

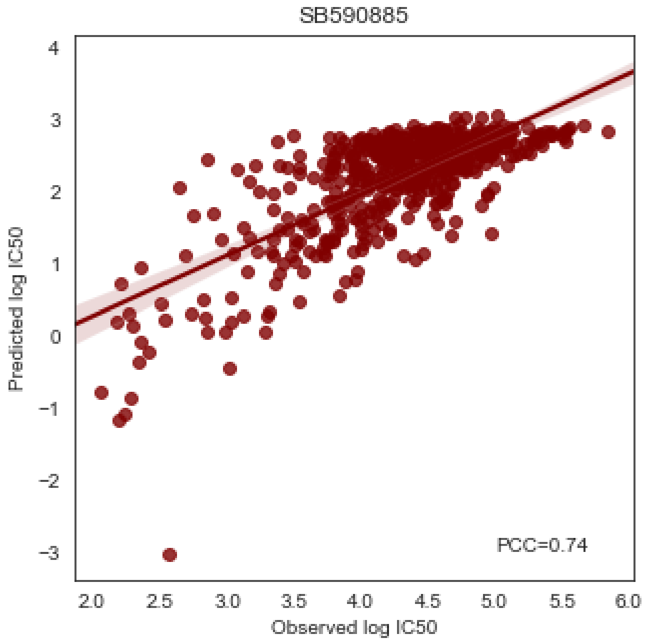

Supp. Fig. S 78: *PCC* for SB590885 prediction
37.png 37.bb

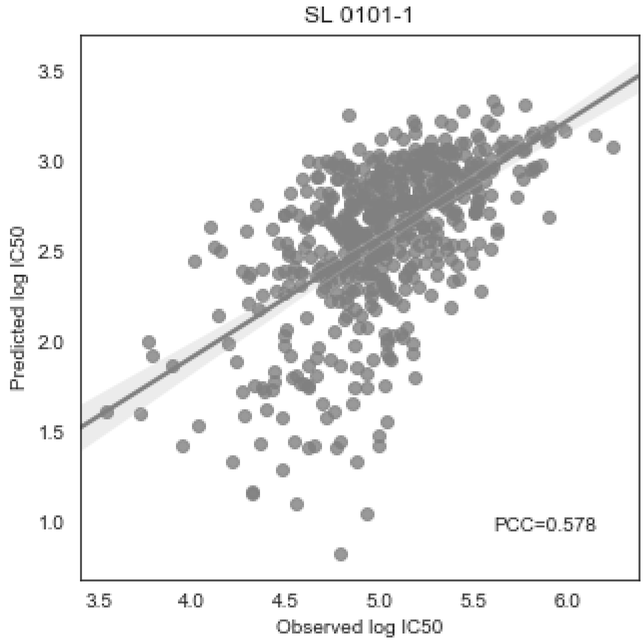

Supp. Fig. S 79: *PCC* for SL 0101-1 prediction

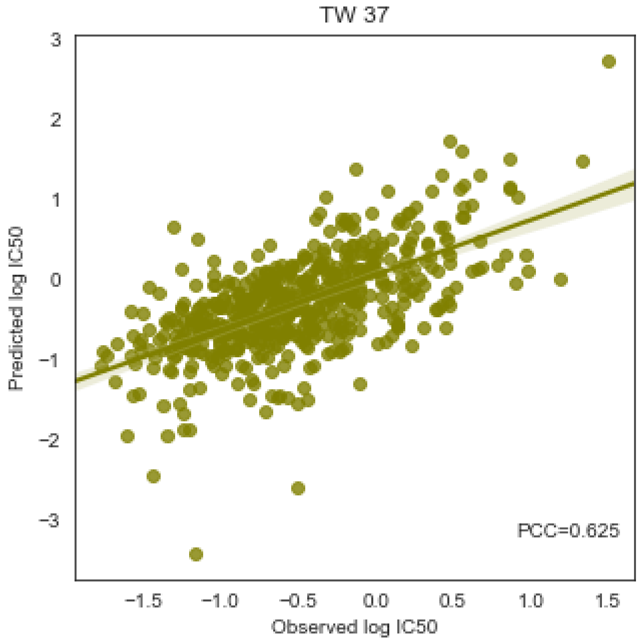

Supp. Fig. S 80: *PCC* for TW 37 prediction

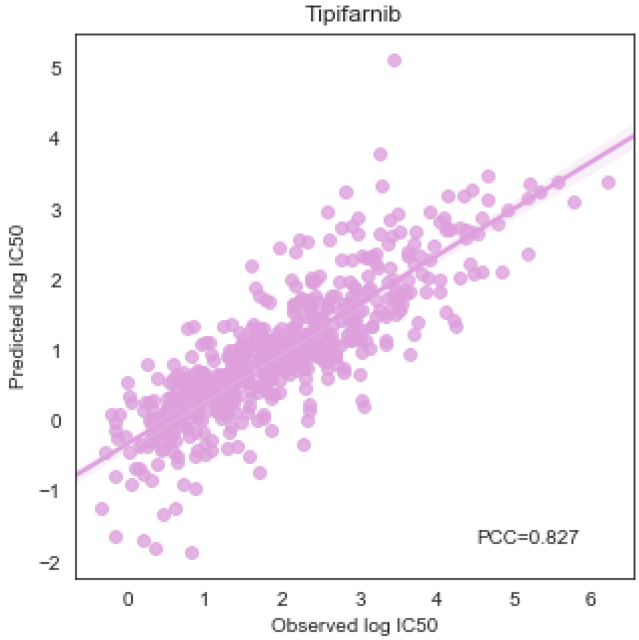

Supp. Fig. S 81: *PCC* for Tipifarnib prediction

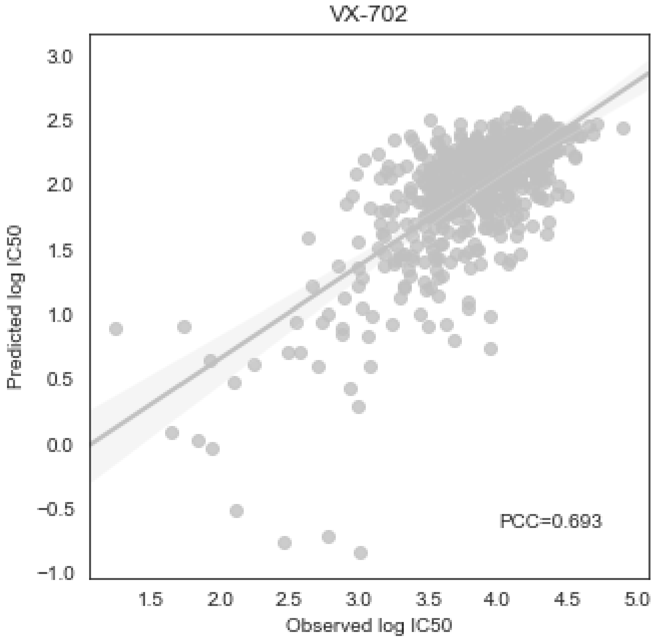

Supp. Fig. S 82: *PCC* for VX-702 prediction

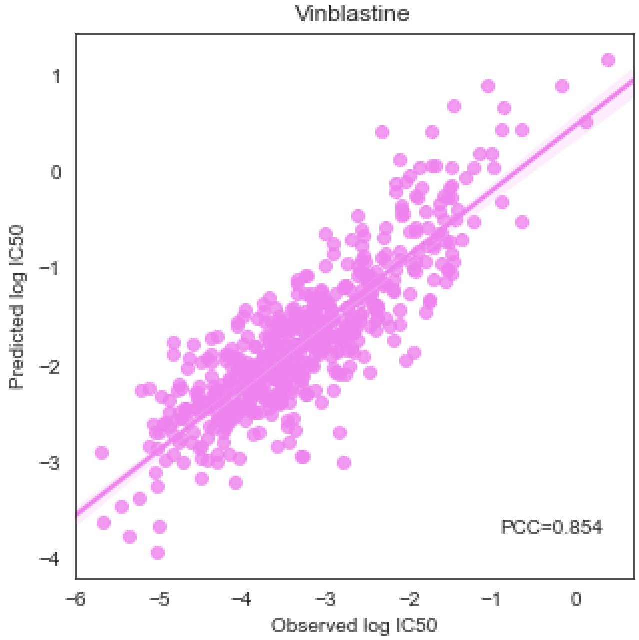

Supp. Fig. S 83: *PCC* for Vinblastine prediction

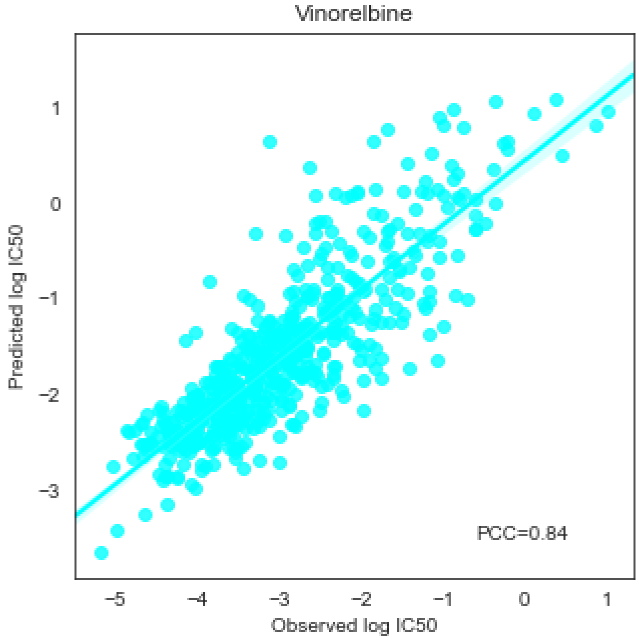

Supp. Fig. S 84: *PCC* for Vinorelbine prediction

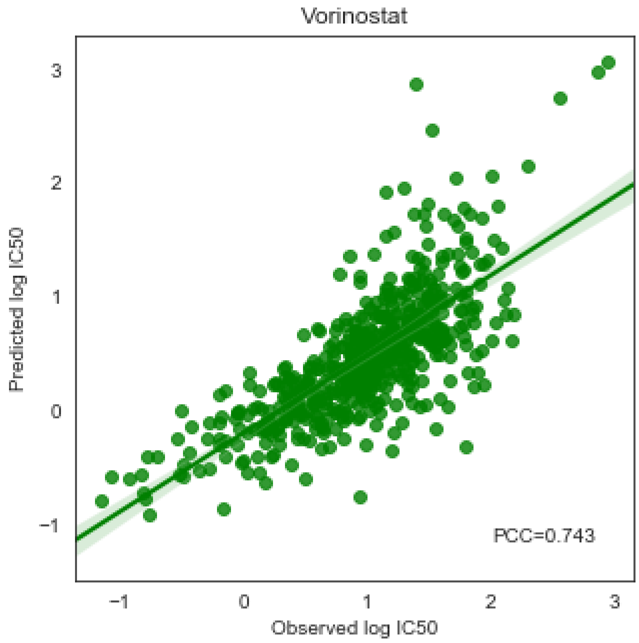

Supp. Fig. S 85: *PCC* for Vorinostat prediction

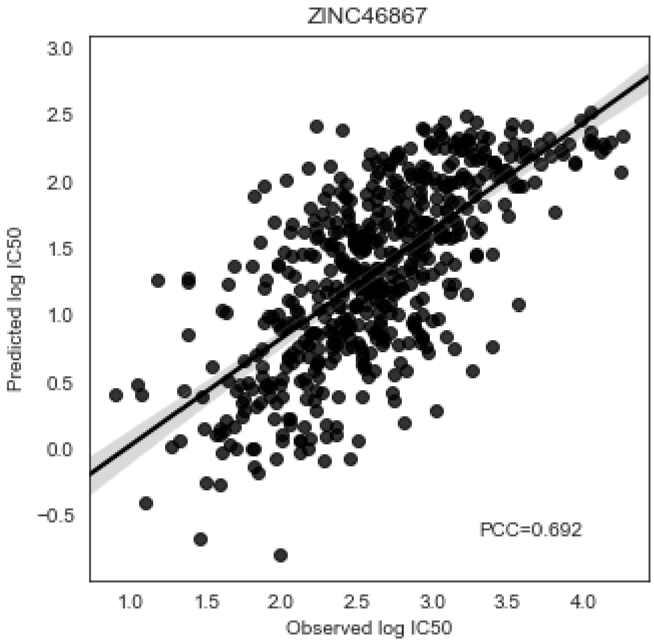

Supp. Fig. S 86: *PCC* for ZINC46867 prediction

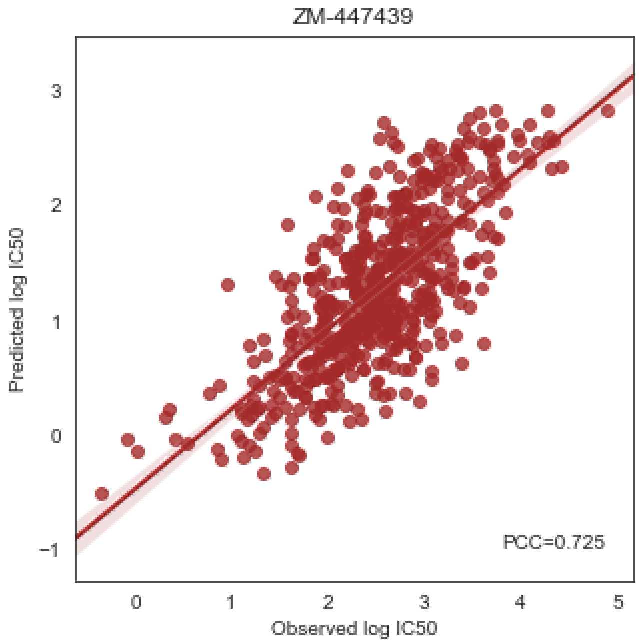

Supp. Fig. S 87: *PCC* for ZM-447439 prediction

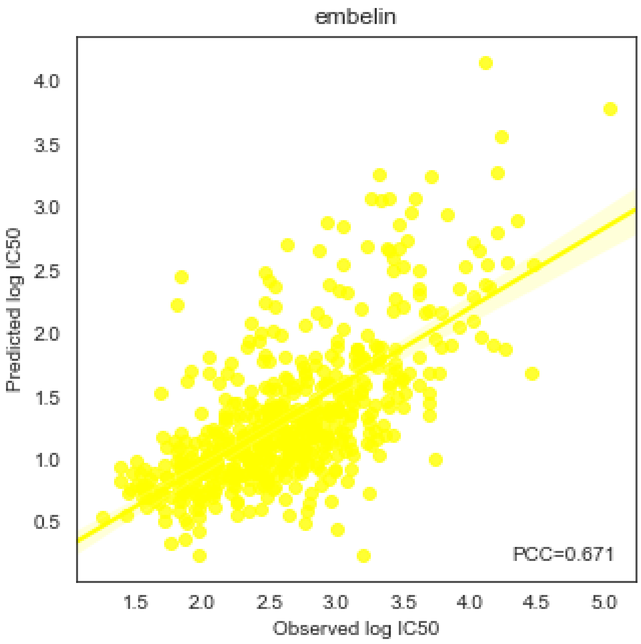

Supp. Fig. S 88: *PCC* for embelin prediction

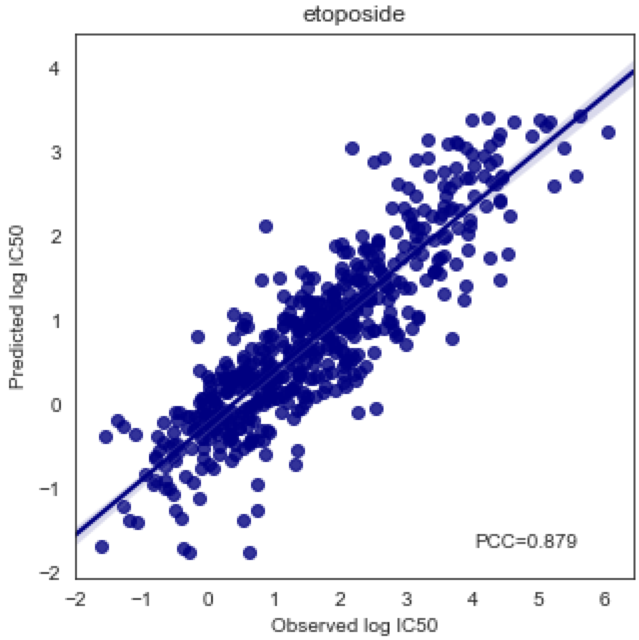

Supp. Fig. S 89: *PCC* for etoposide prediction

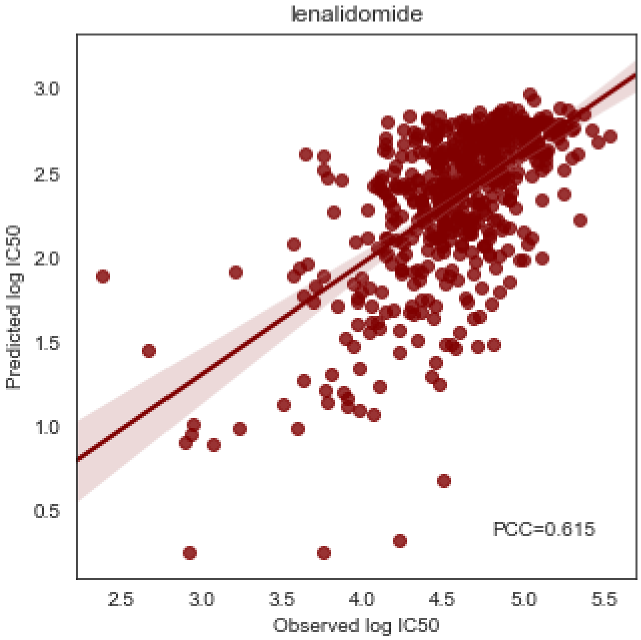

Supp. Fig. S 90: *PCC* for lenalidomide prediction

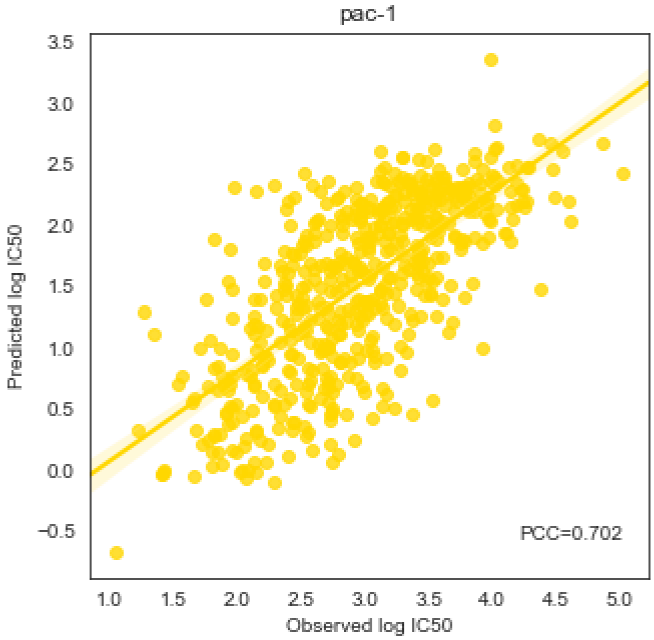

Supp. Fig. S 91: *PCC* for pac-1 prediction

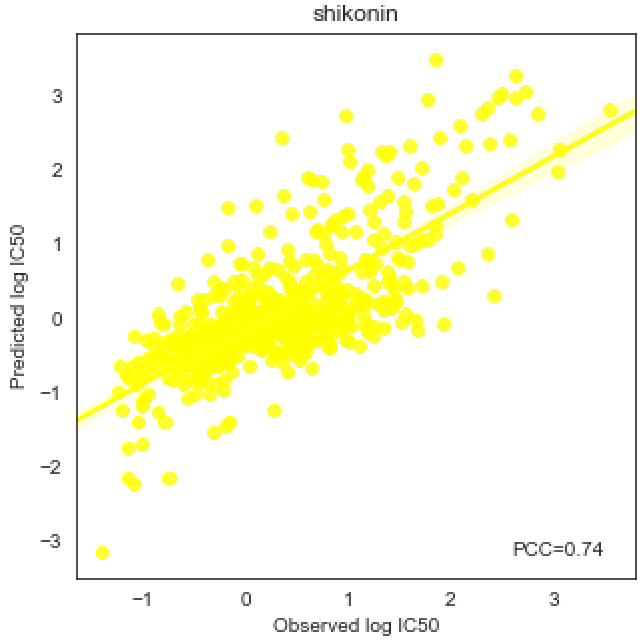

Supp. Fig. S 92: *PCC* for shikonin prediction

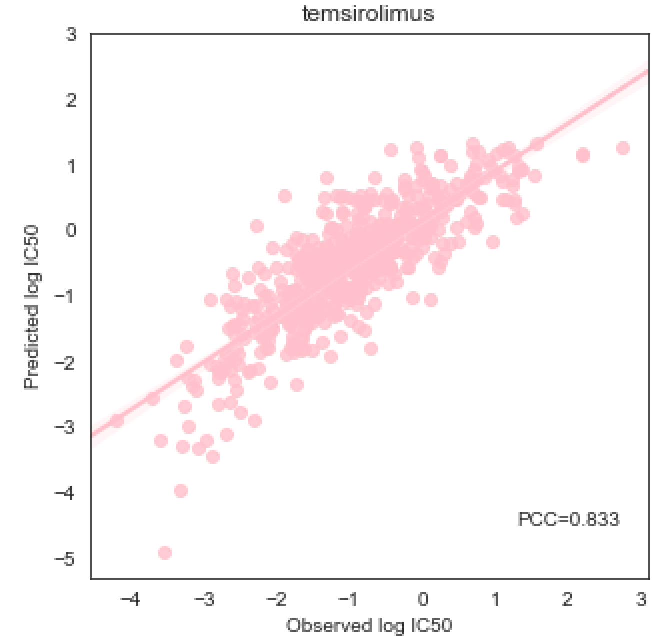

Supp. Fig. S 93: *PCC* for temsirolimus prediction

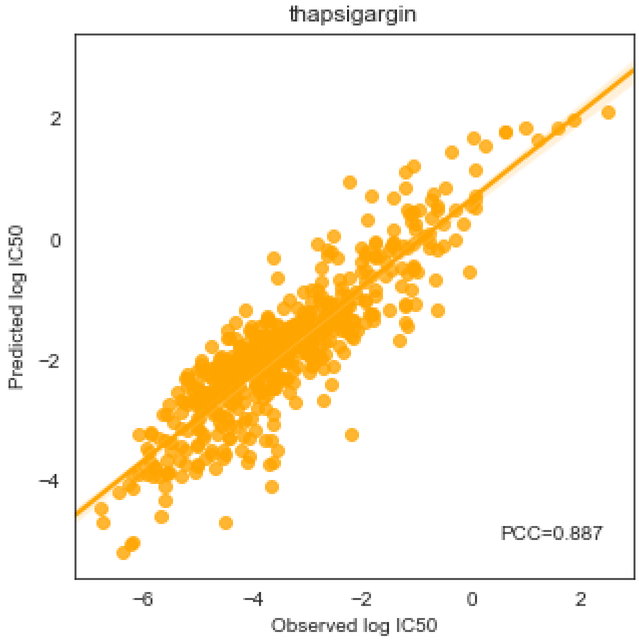

Supp. Fig. S 94: *PCC* for thapsigargin prediction

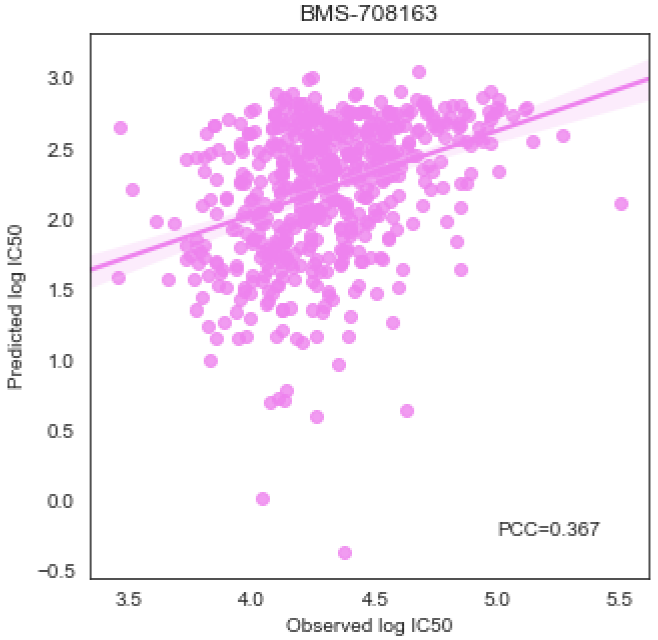

Supp. Fig. S 95: *PCC* for BMS-708163 prediction

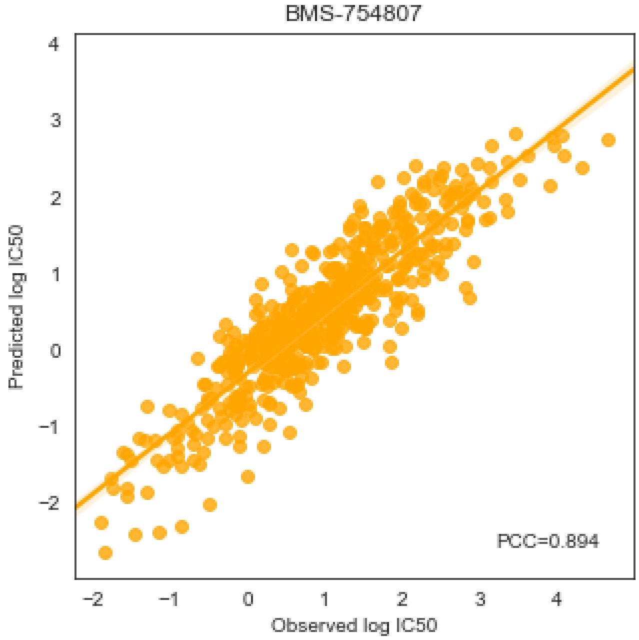

Supp. Fig. S 96: *PCC* for BMS-754807 prediction

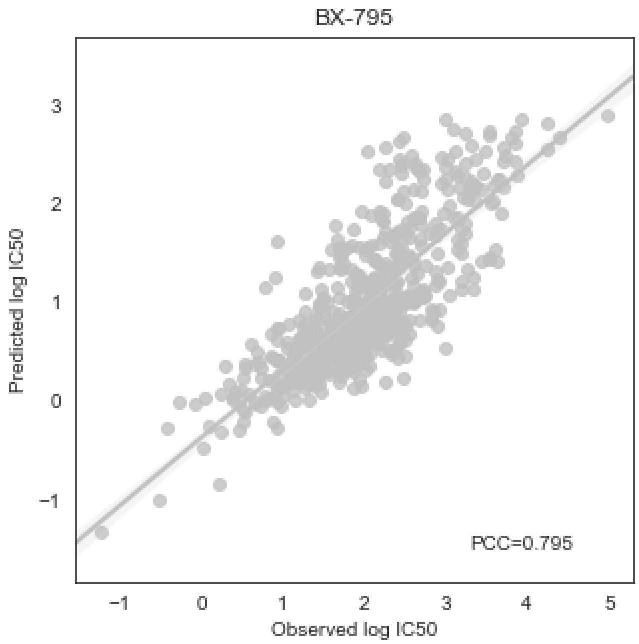

Supp. Fig. S 97: *PCC* for BX-795 prediction

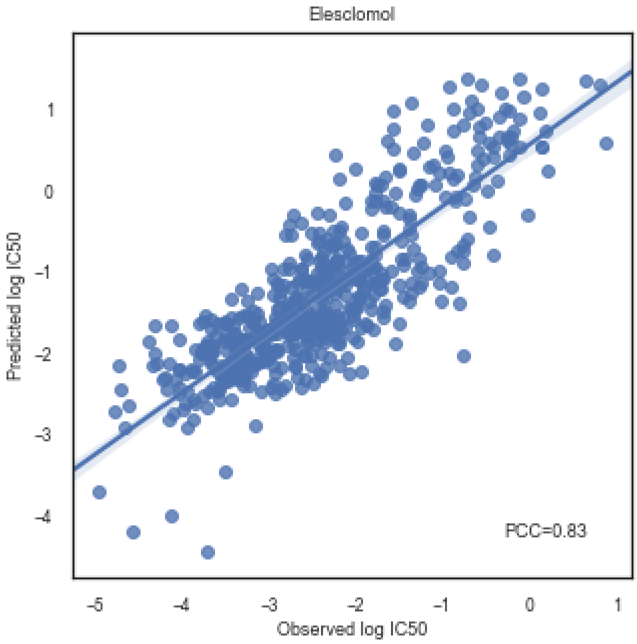

Supp. Fig. S 98: *PCC* for Elesclomol prediction

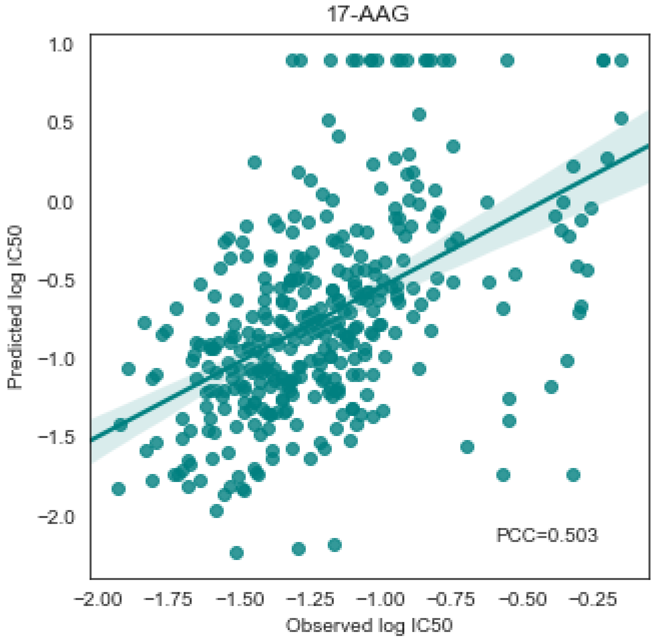

Supp. Fig. S 99: *PCC* for 17-AAG prediction

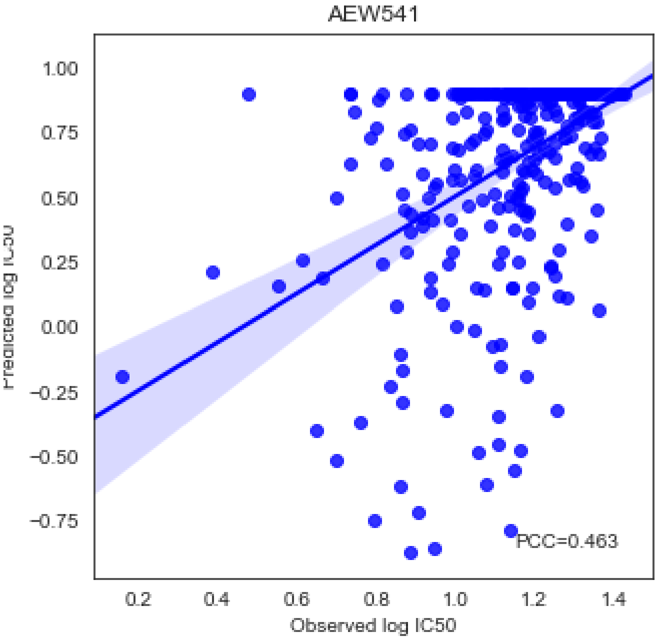

Supp. Fig. S 100: *PCC* for AEW541 prediction

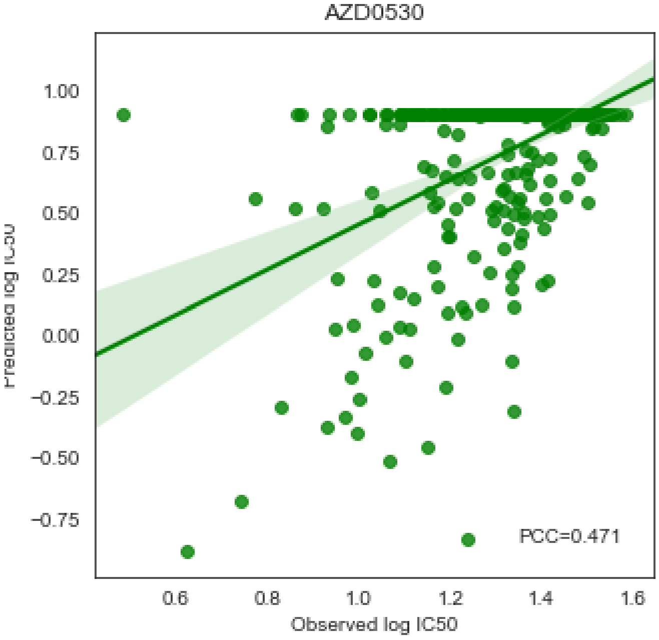

Supp. Fig. S 101: *PCC* for AZD0530 prediction

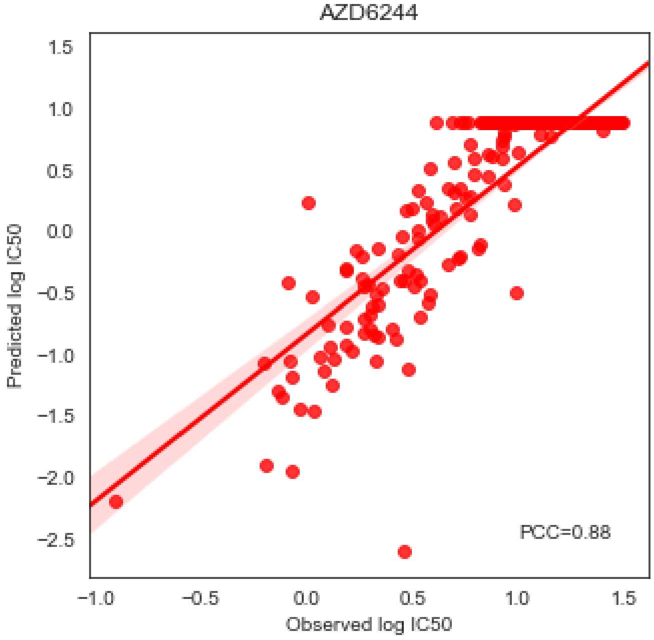

Supp. Fig. S 102: *PCC* for AZD6244 prediction

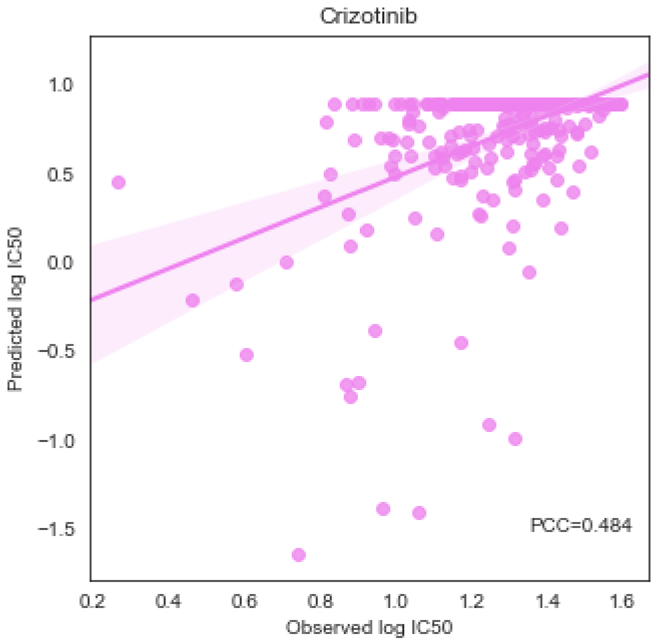

Supp. Fig. S 103: *PCC* for Crizotinib prediction

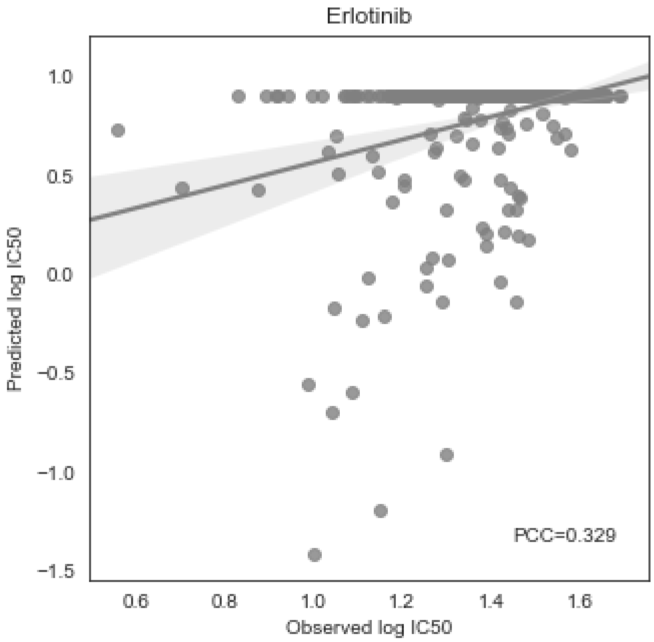

Supp. Fig. S 104: *PCC* for Erlotinib prediction

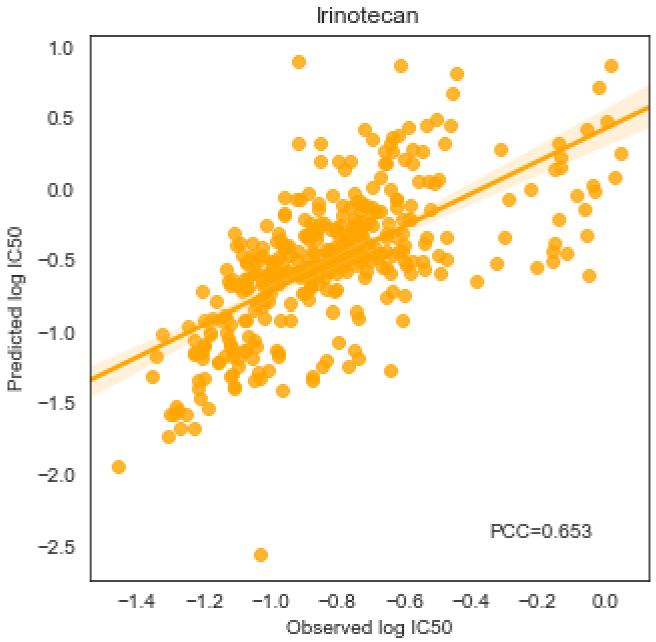

Supp. Fig. S 105: *PCC* for Irinotecan prediction

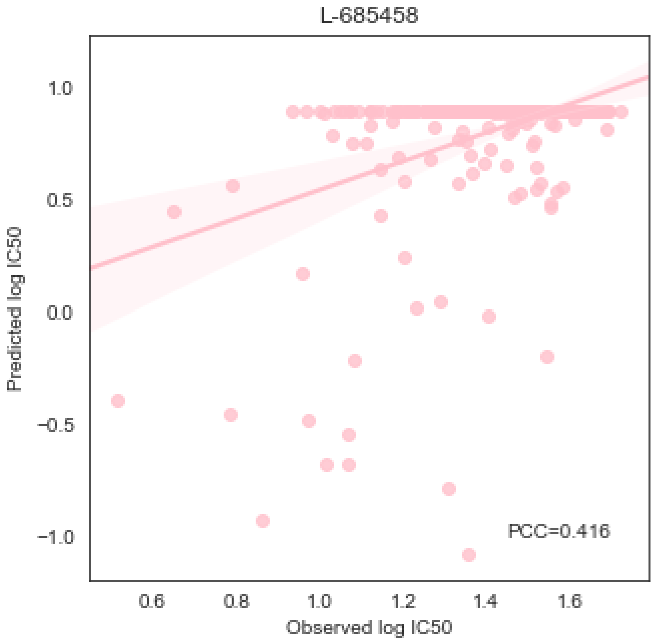

Supp. Fig. S 106: *PCC* for L-685458 prediction

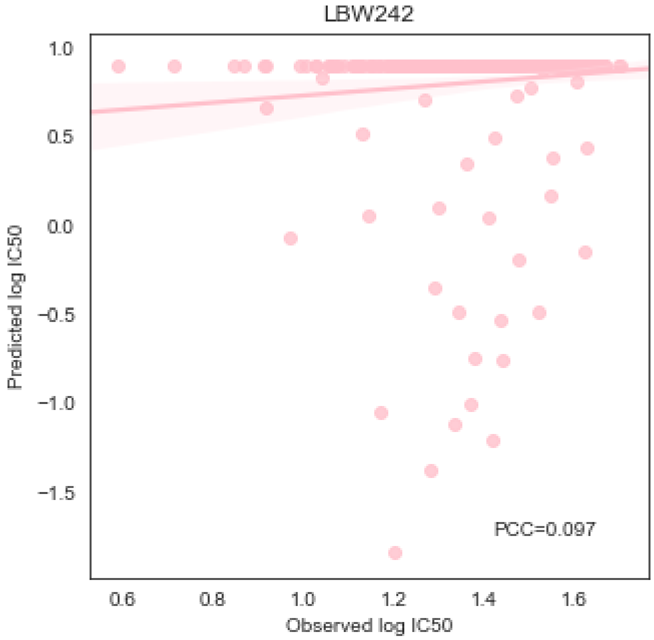

Supp. Fig. S 107: *PCC* for LBW242 prediction

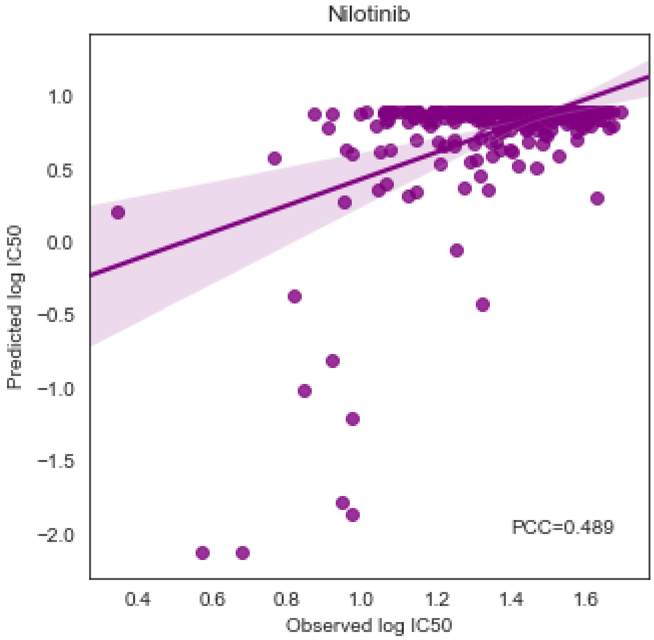

Supp. Fig. S 108: *PCC* for Nilotinib prediction

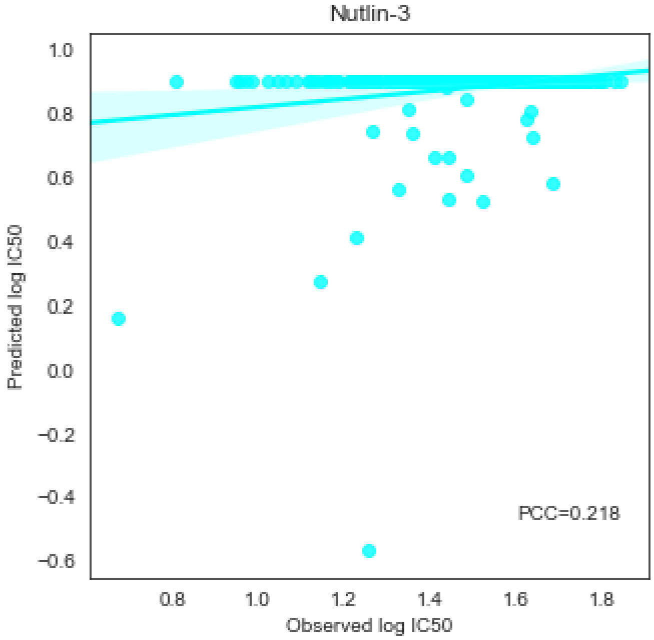

Supp. Fig. S 109: *PCC* for Nutlin-3 prediction

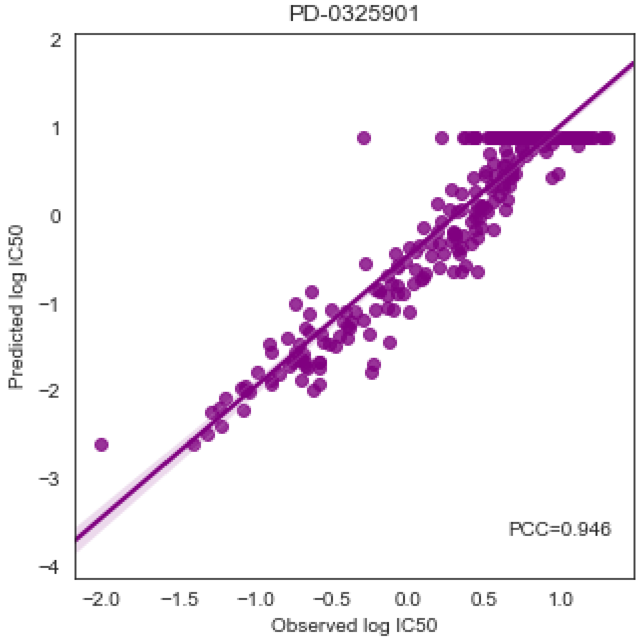

Supp. Fig. S 110: *PCC* for PD-0325901 prediction

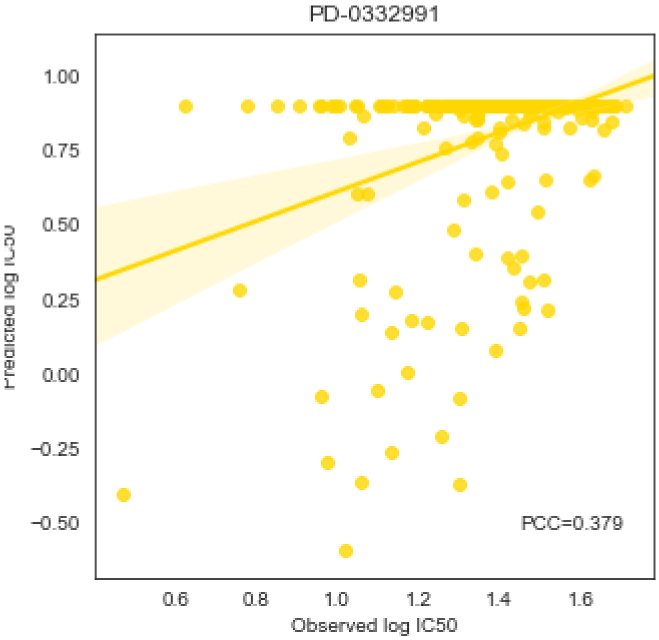

Supp. Fig. S 111: *PCC* for PD-0332991 prediction

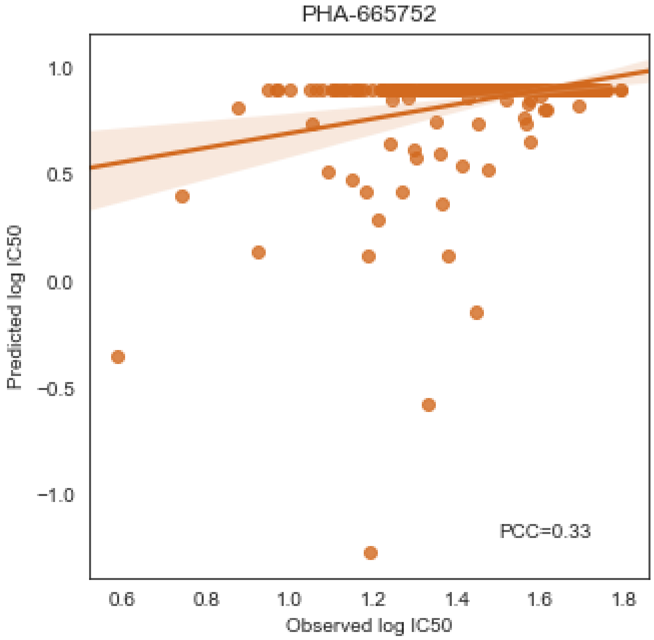

Supp. Fig. S 112: *PCC* for PHA-665752 prediction

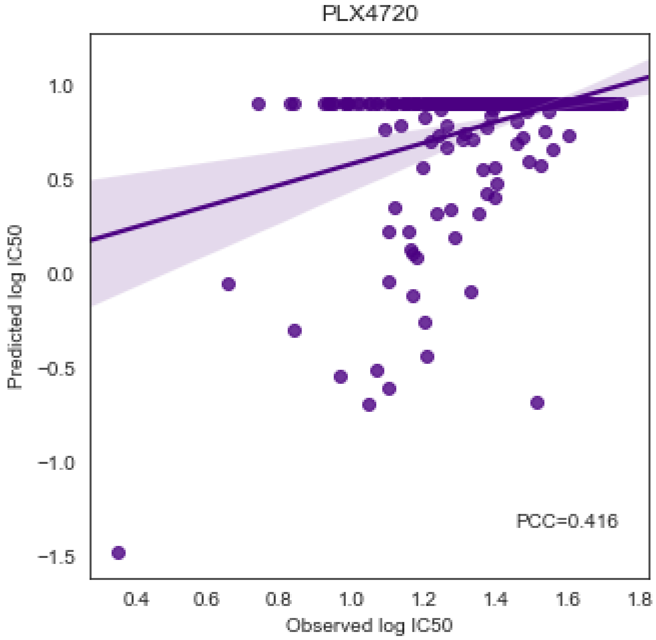

Supp. Fig. S 113: *PCC* for PLX4720 prediction

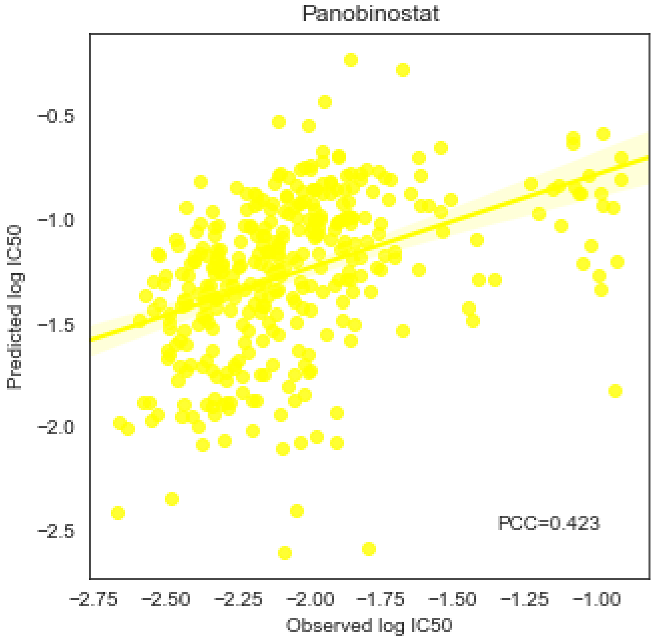

Supp. Fig. S 114: *PCC* for Panobinostat prediction

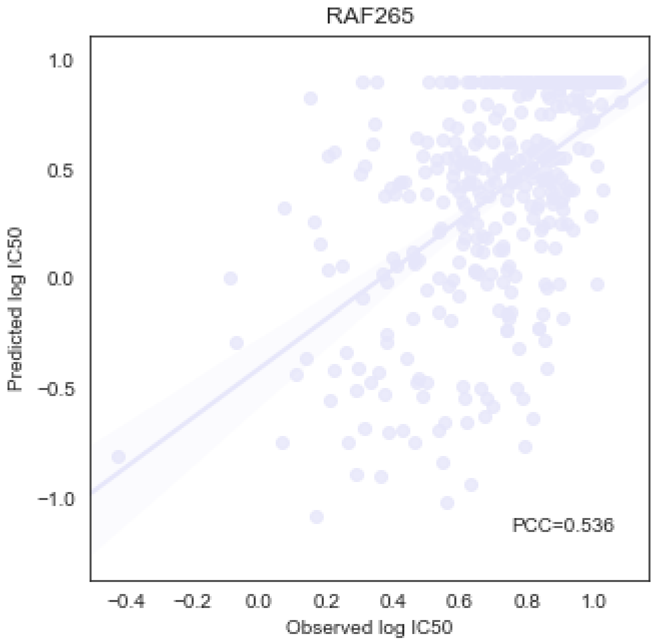

Supp. Fig. S 115: *PCC* for RAF265 prediction

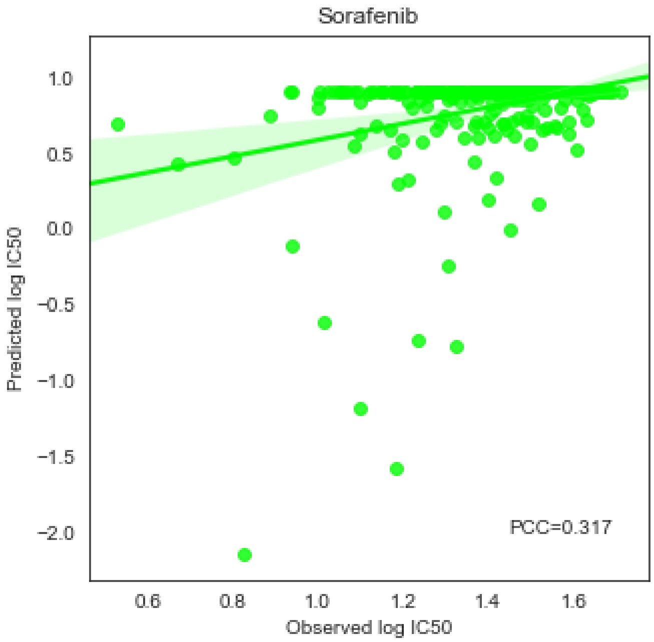

Supp. Fig. S 116: *PCC* for Sorafenib prediction

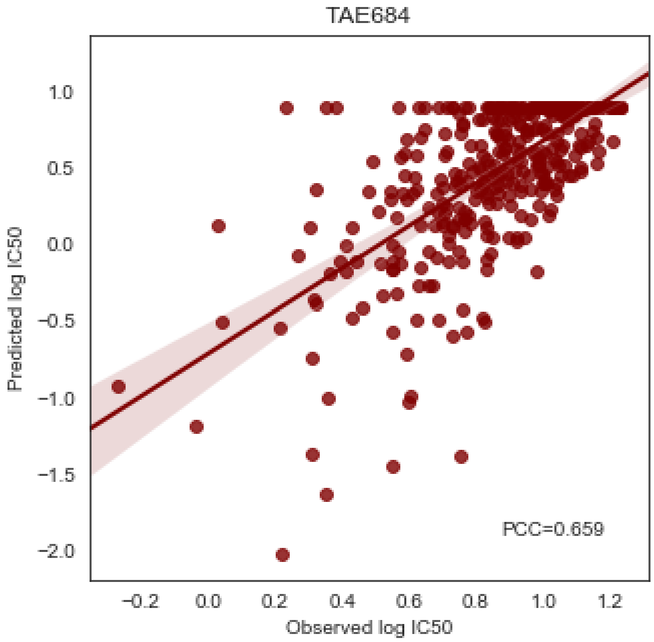

Supp. Fig. S 117: *PCC* for TAE684 prediction

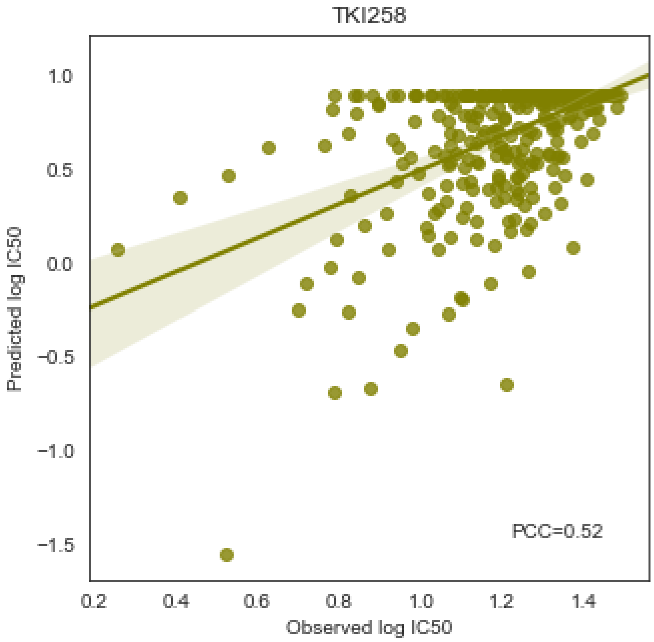

Supp. Fig. S 118: *PCC* for TKI258 prediction

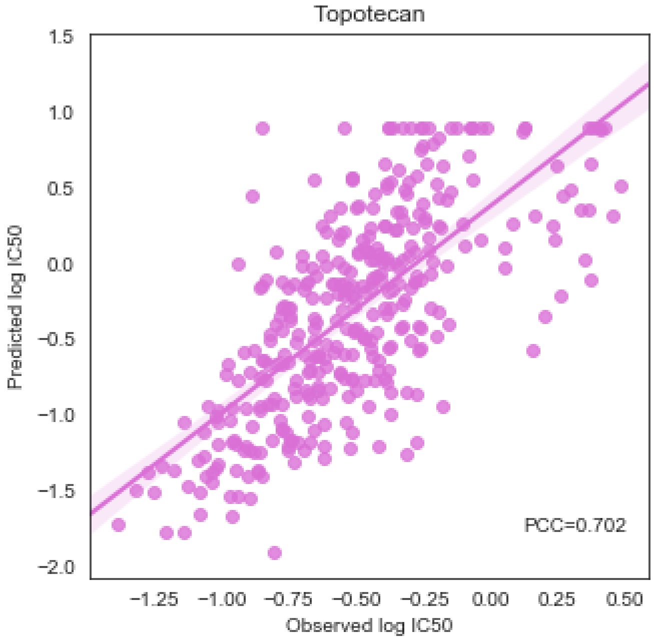

Supp. Fig. S 119: *PCC* for Topotecan prediction

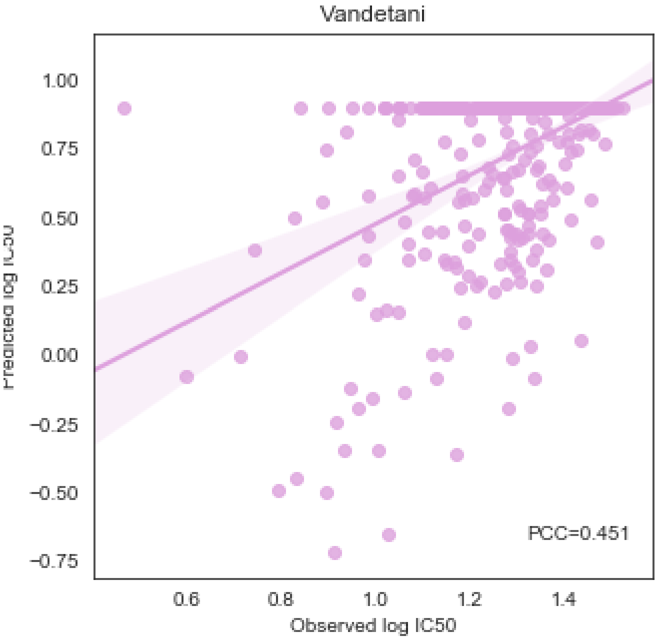

Supp. Fig. S 120: *PCC* for Vandetani prediction

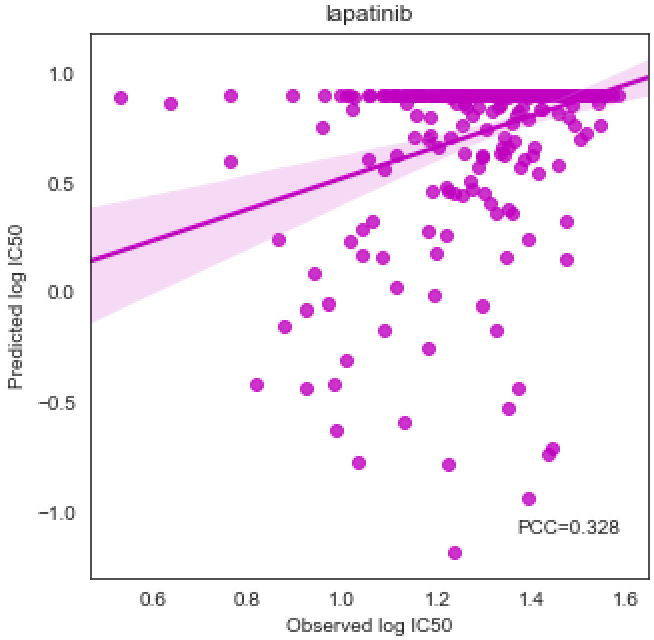

Supp. Fig. S 121: *PCC* for lapatinib prediction

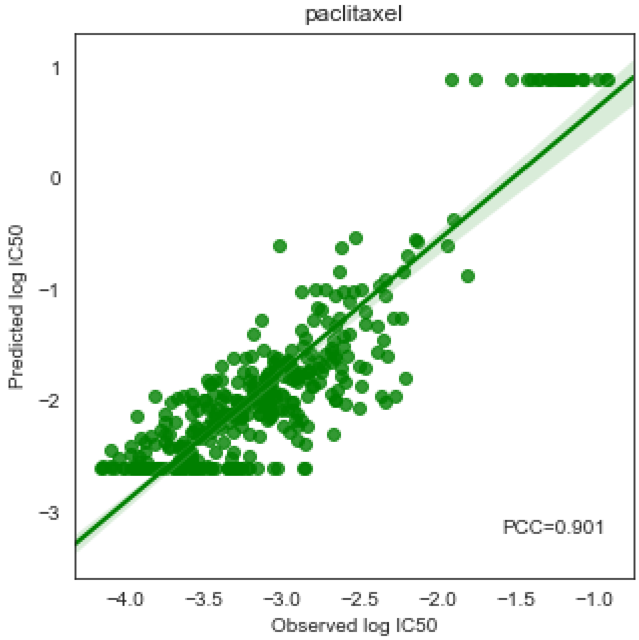

Supp. Fig. S 122: *PCC* for paclitaxel prediction

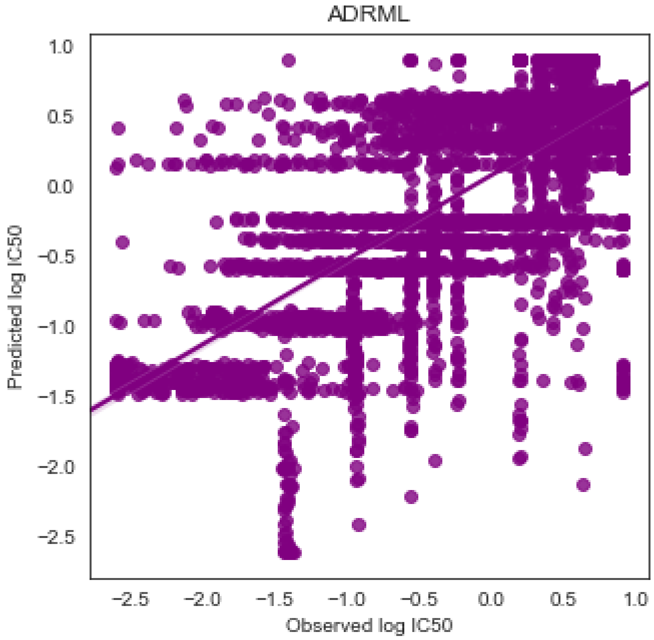

Supp. Fig. S 123: Scatterplot of ADRML's predictions.

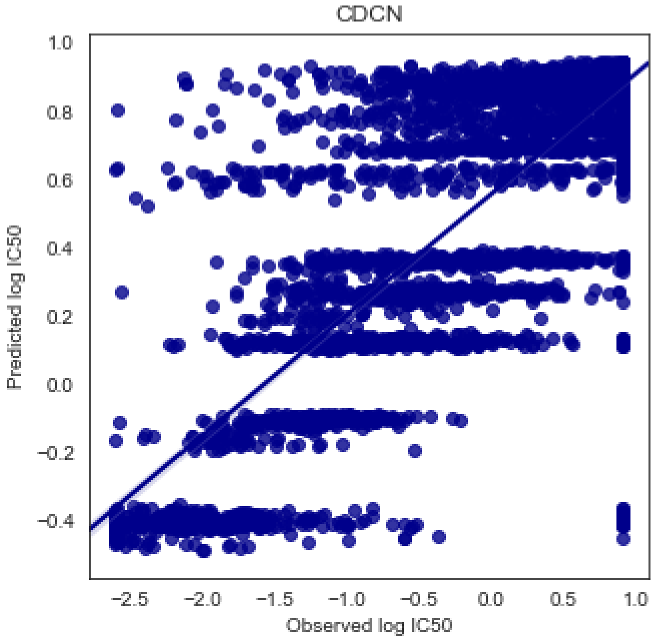

Supp. Fig. S 124: Scatterplot of CDCN's predictions.

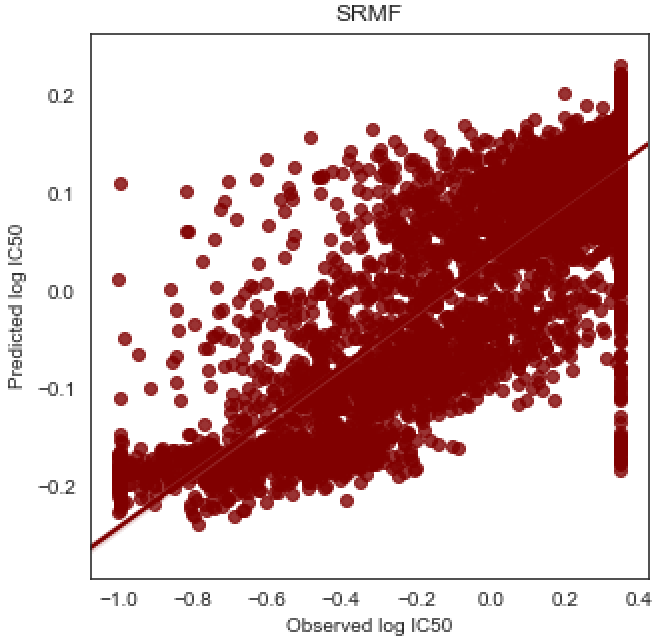

Supp. Fig. S 125: Scatterplot of SRMF's predictions.

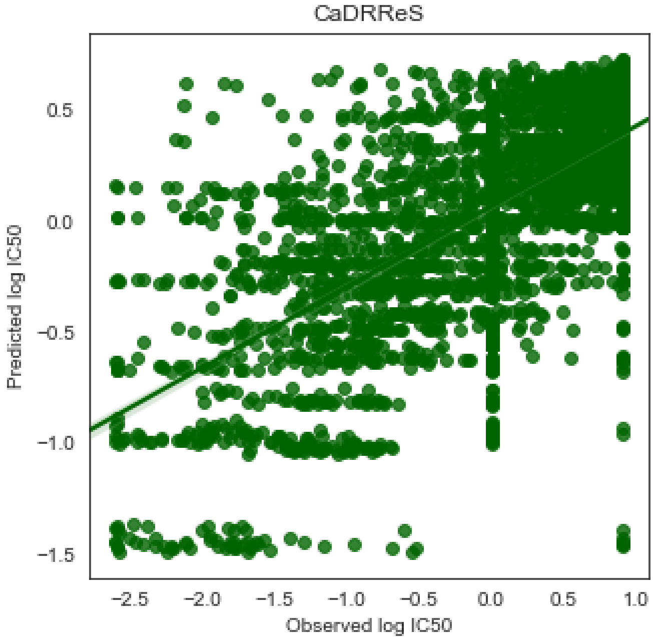

Supp. Fig. S 126: Scatterplot of CaDRReS' predictions.

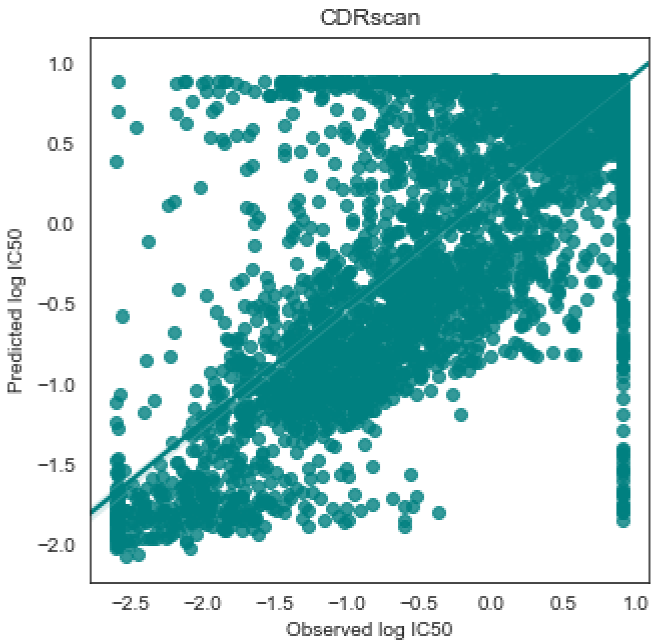

Supp. Fig. S 127: Scatter plot of CDRscan's predictions.

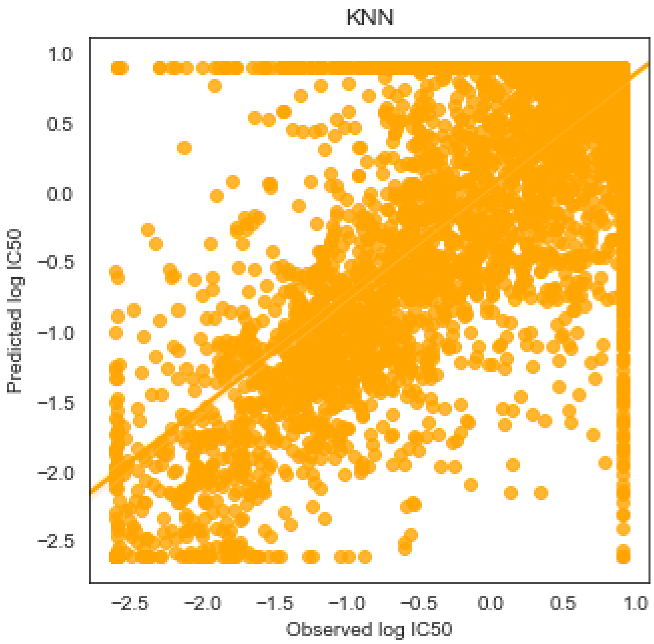

Supp. Fig. S 128: Scatter plot of KNN's predictions.
